# Supplementary figures and images for: A major endogenous glycoside hydrolase mediating quercetin uptake in Bombyx mori
Source: PLoS Genet. 2024 Jan 17;20(1):e1011118. doi: 10.1371/journal.pgen.1011118 (PMC10824415; doi:10.1371/journal.pgen.1011118)

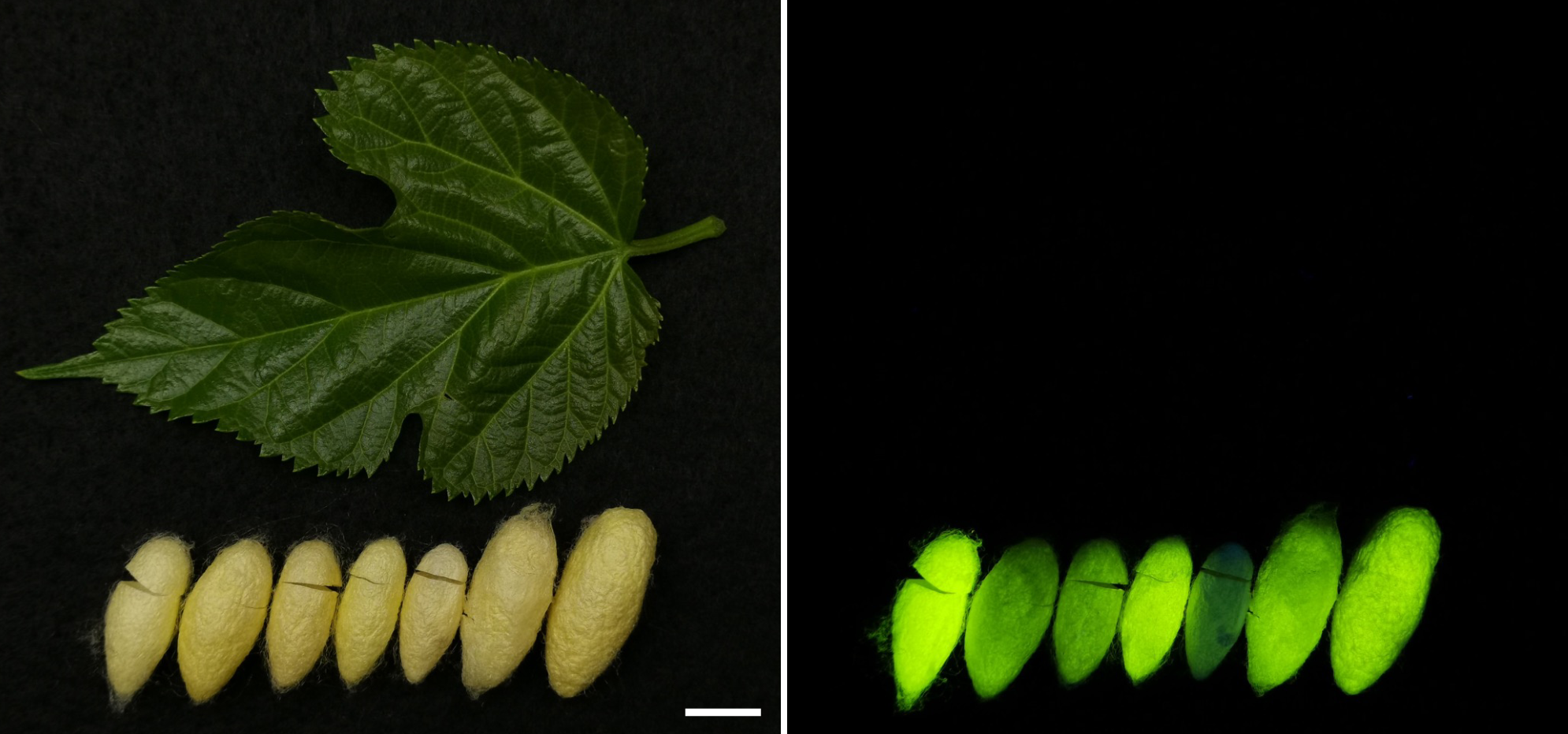

Supplement: S1 Fig — A fresh mulberry leaf and cocoons of the wild silkworm in a bright field (left) and irradiated with ultraviolet A in a dark field (right). The cocoons under ultraviolet irradiation exhibit fluorescence characteristic of quercetin-5-O-glucoside and quercetin-5,4´-di-O-glucoside, the major quercetin metabolites in the silkworm tissues and cocoon [14,15,29]. The cocoons were collected in June 2023 at Tsukuba, Ibaraki, Japan. Bar = 10 mm. (TIF) [file pgen.1011118.s001.tif]

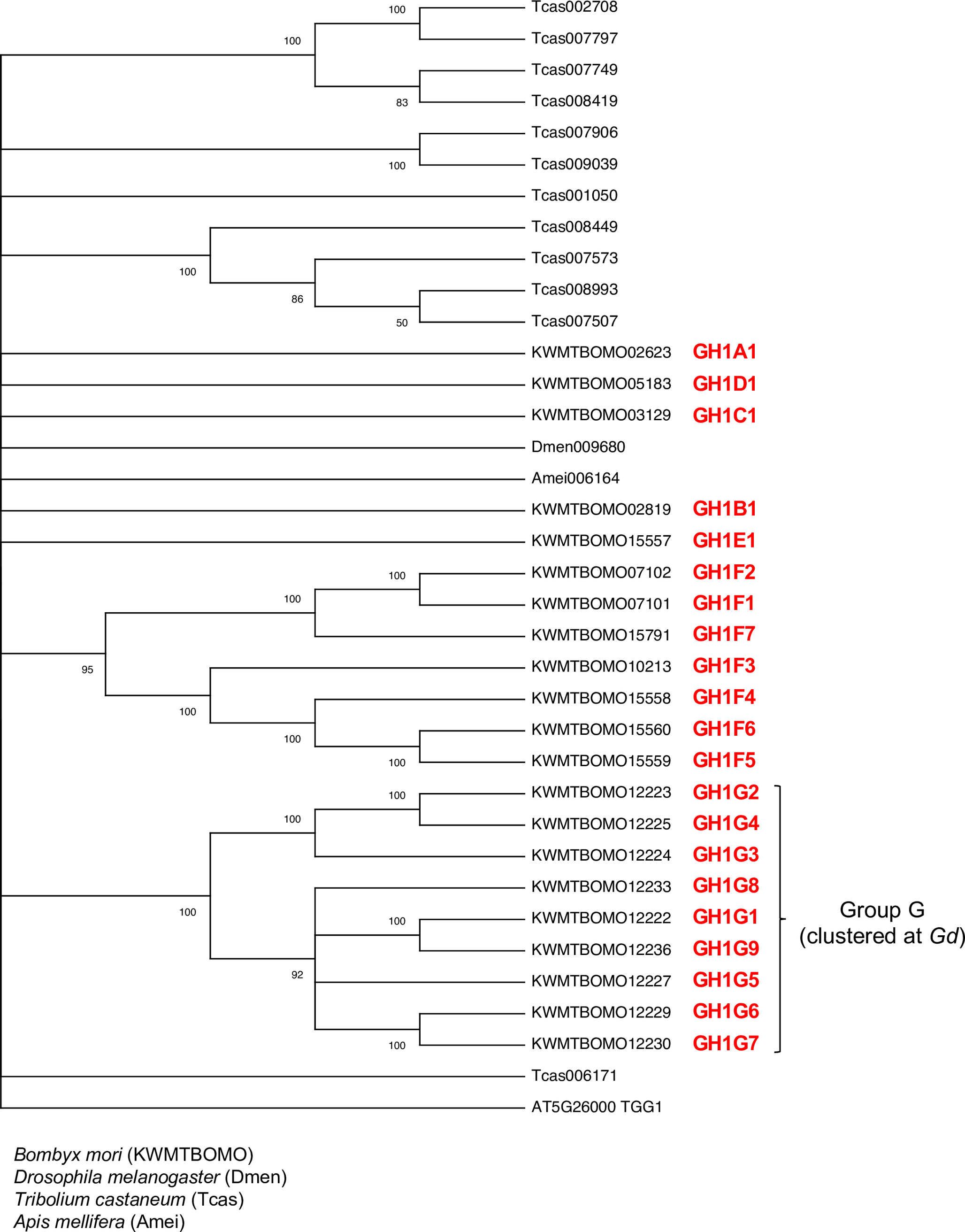

Supplement: S2 Fig — Values on the nodes represent the bootstrap score (trials = 100). Unreliable nodes with bootstrap values under 50 are shown as multi-branching nodes. Arabidopsis thioglucoside glucohydrolase 1 (TGG1) was used as the outgroup. (TIF) [file pgen.1011118.s002.tif]

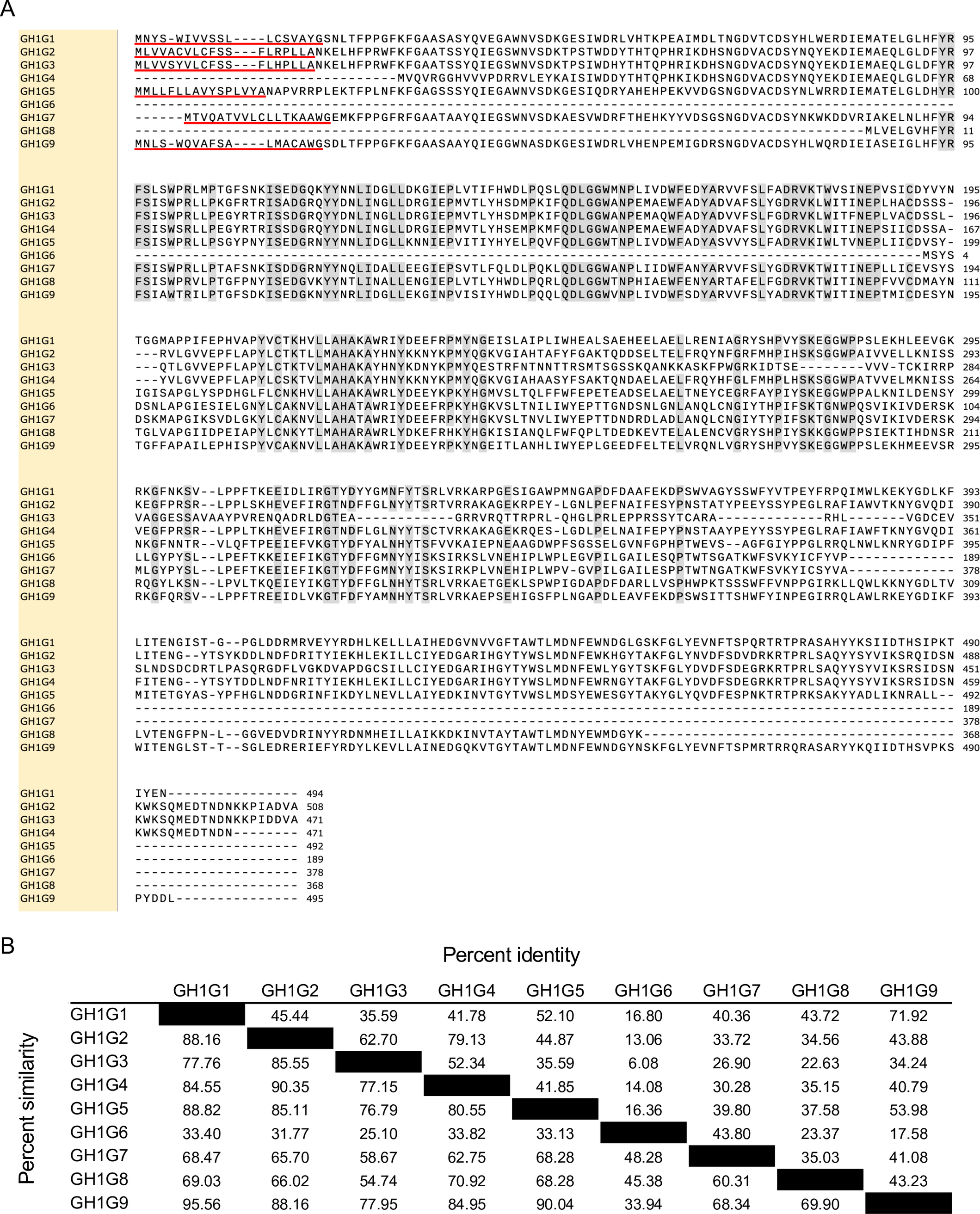

Supplement: S3 Fig — (A) Alignment of the group G glycoside hydrolase proteins in B. mori using Clustal Omega. Predicted signal peptides are highlighted by red lines. The threshold for identity shading was set to 80%. (B) Sequence identity and similarity of the group G glycoside hydrolase proteins in B. mori. Similarity of amino acid residue property is determined according to the groups of strongly similar properties described at Clustal Omega FAQ (https://www.ebi.ac.uk/seqdb/confluence/display/THD/Help+-+Clustal+Omega+FAQ). (TIF) [file pgen.1011118.s003.tif]

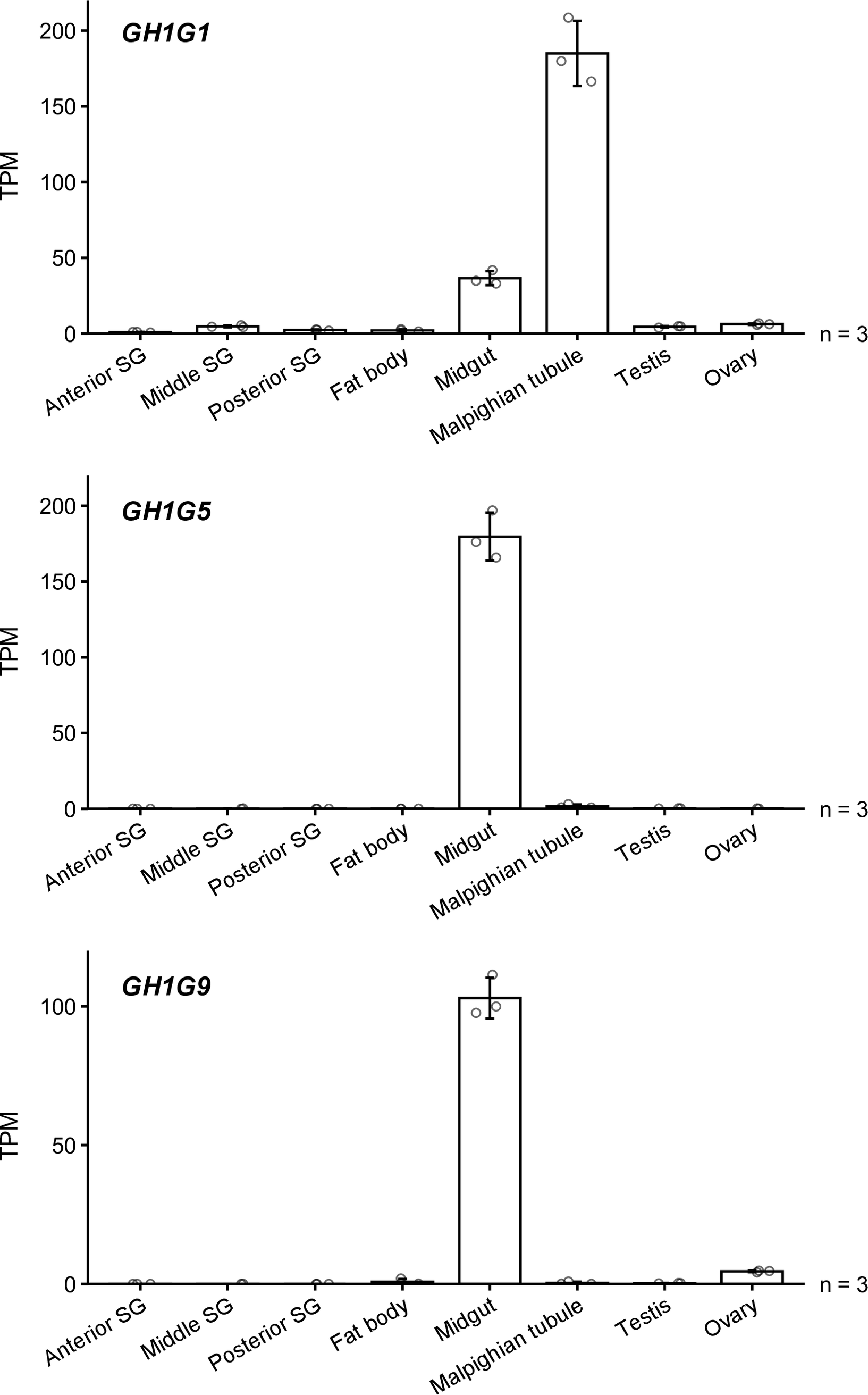

Supplement: S4 Fig — All organs and tissues, except for ovaries, were taken from male larvae. Data are means of n independent biological replicates ± SD. SG, silk gland; TPM, transcripts per million. (TIF) [file pgen.1011118.s004.tif]

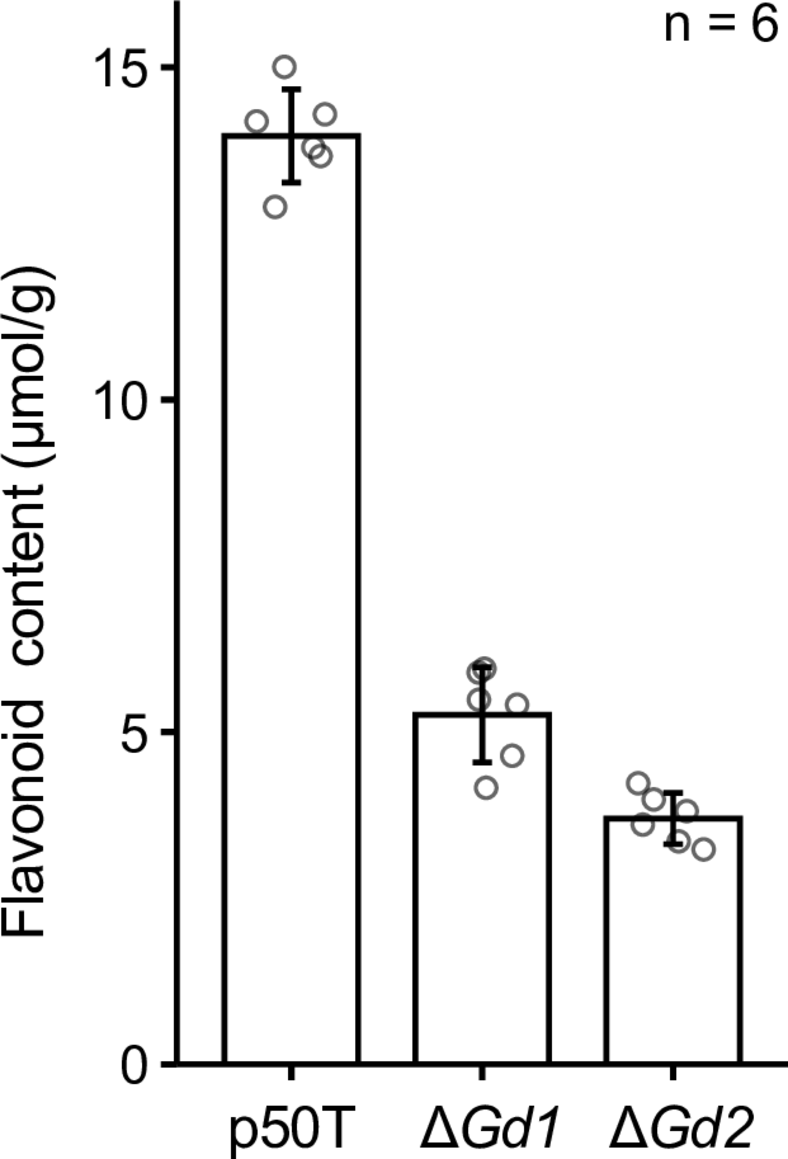

Supplement: S5 Fig — Data are means of n independent biological replicates ± SD. (TIF) [file pgen.1011118.s005.tif]

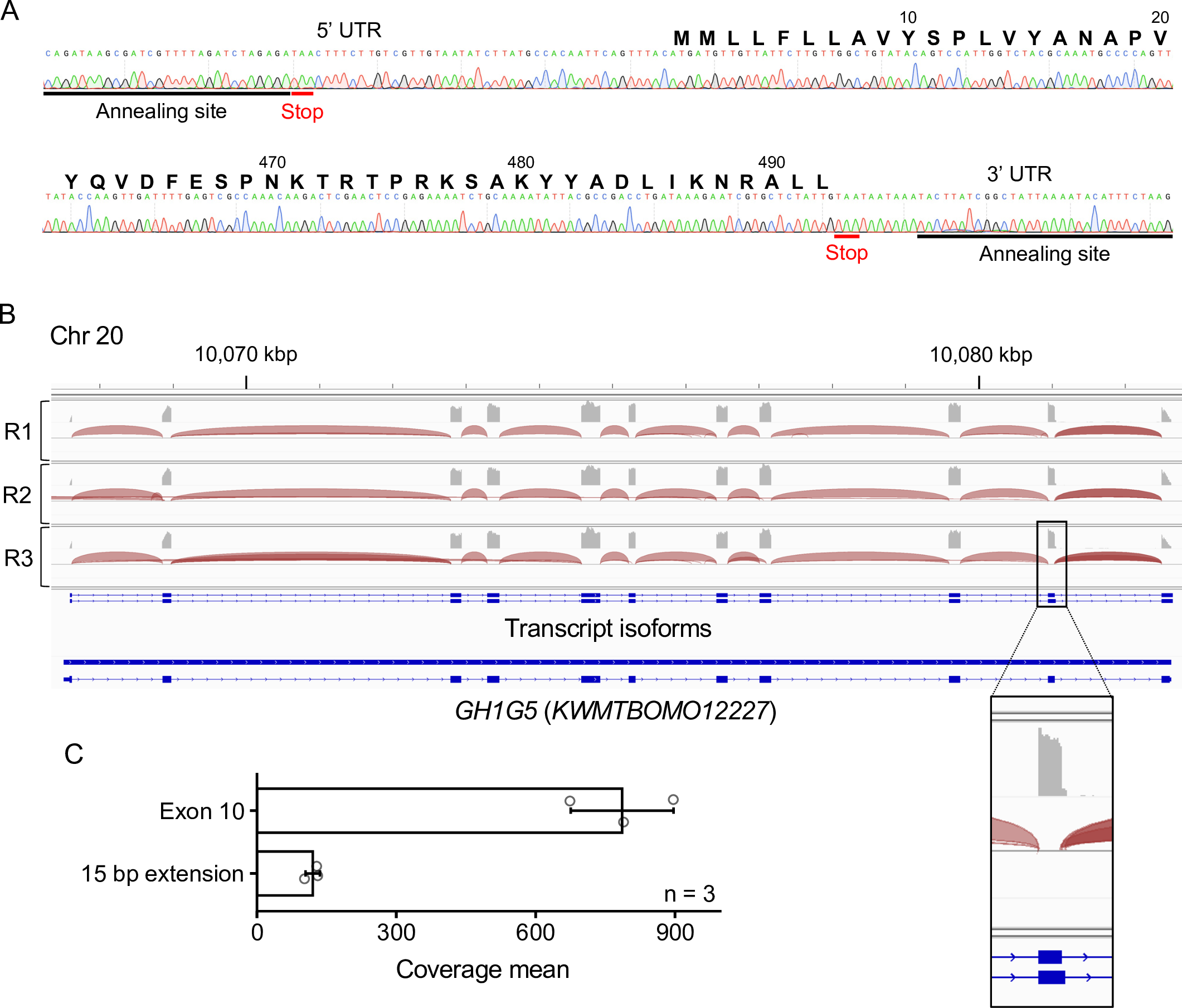

Supplement: S6 Fig — (A) Regions around the translation start site (up) and stop site (down) of the cloned transcript sequence of GH1G5. The annealing sites of primers used for cloning are highlighted by black lines. (B) Mapping results of the midgut-derived RNA-seq reads and GH1G5 transcript isoforms visualized with Integrative Genomics Viewer. Coverage on each exon and splice junction of three independent biological replicates is illustrated on tracks R1–3. (C) Coverage mean of each base of exon 10 and the 15-bp extension region. Data are means of n independent biological replicates ± SD. (TIF) [file pgen.1011118.s006.tif]

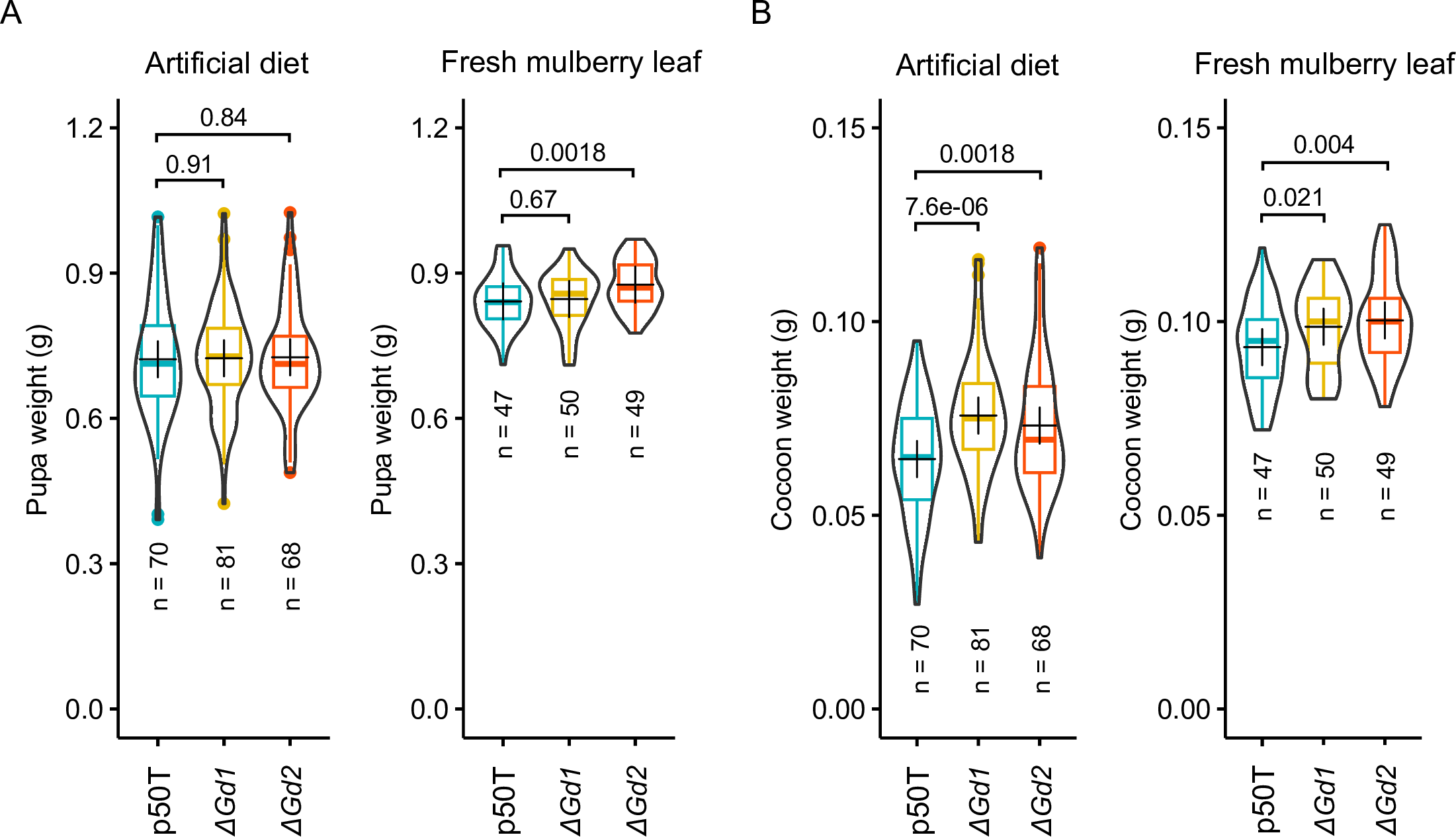

Supplement: S7 Fig — (A) Weight of the pupae. (B) Weight of the cocoons. The values above the graph are p-values calculated by Student’s t-test. Crosses inside the boxes indicate means of n independent biological replicates. (TIF) [file pgen.1011118.s007.tif]

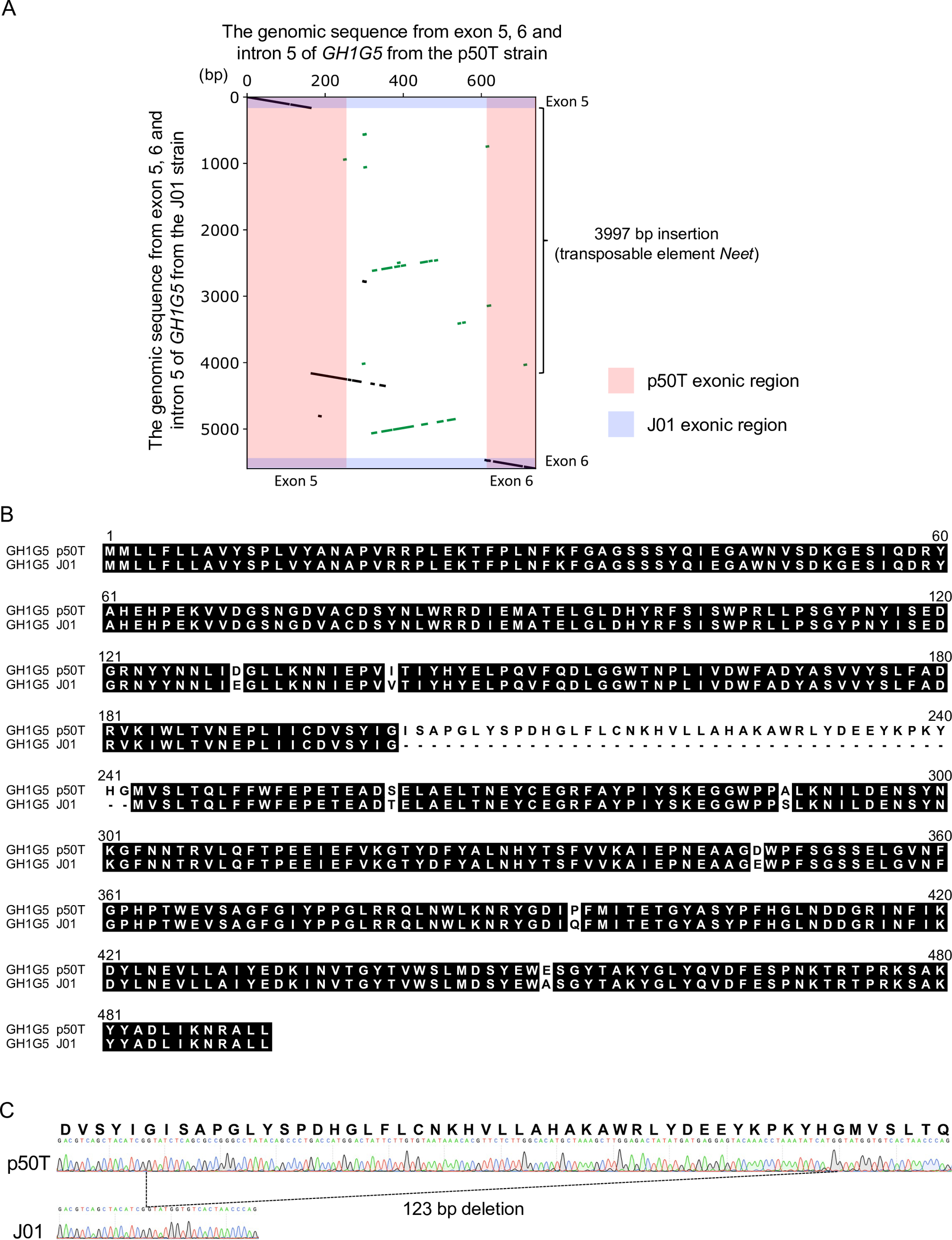

Supplement: S8 Fig — (A) Dot-plot analysis of the genomic region of exons 5, 6 and the intronic region between them (intron 5) of GH1G5 from the p50T and J01 strains. Green dots indicate inverted regions. (B) Alignment of predicted protein sequences of GH1G5 from the p50T and J01 strains using Clustal Omega [56]. (C) Deletion in the J01 GH1G5 transcript confirmed using Sanger sequencing. (TIF) [file pgen.1011118.s008.tif]

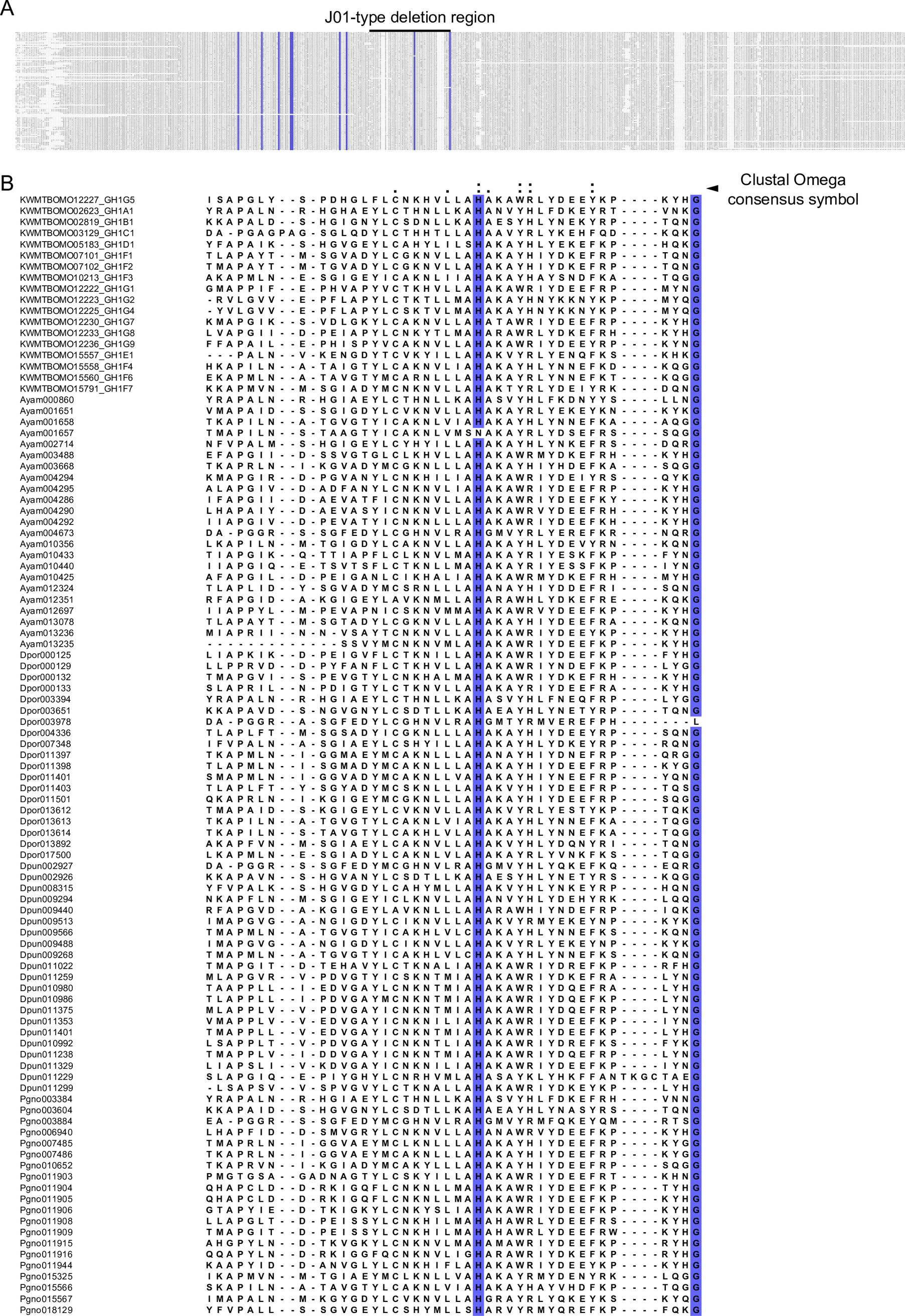

Supplement: S9 Fig — (A) Whole alignment of 101 GH1G5-like proteins in Macroheterocera species. Amino acid residues identical to GH1G5 at positions with >99% conservation are highlighted by blue. The 100 homologous proteins were collected by a BLASTp search using the amino acid sequence of GH1G5 from sequence data of each protein from Bombyx mori (KWMT), Antheraea yamamai (Ayam), Deilephila porcellus (Dpor), Dendrolimus punctatus (Dpun) and Pheosia gnoma (Pgno) with a cutoff criterion e-value of <1E-100. The sequence data of B. mori were obtained from KAIKObase (https://kaikobase.dna.affrc.go.jp/index.html) and those of A. yamamai, D. porcellus and P. gnoma were obtained from InsectBase 2.0 (http://v2.insect-genome.com/). The sequences were aligned using Clustal Omega. (B) Magnified view of the J01-type deletion region of the alignment. Descriptions of the consensus symbols indicating the conservation level of amino acid residues are available at the Clustal Omega FAQ (https://www.ebi.ac.uk/seqdb/confluence/display/THD/Help+-+Clustal+Omega+FAQ). In brief, ‘:’ indicates residues harboring a strongly similar physicochemical property to GH1G5, and ‘.’ indicates residues harboring a weakly similar physicochemical property to GH1G5. (TIF) [file pgen.1011118.s009.tif]

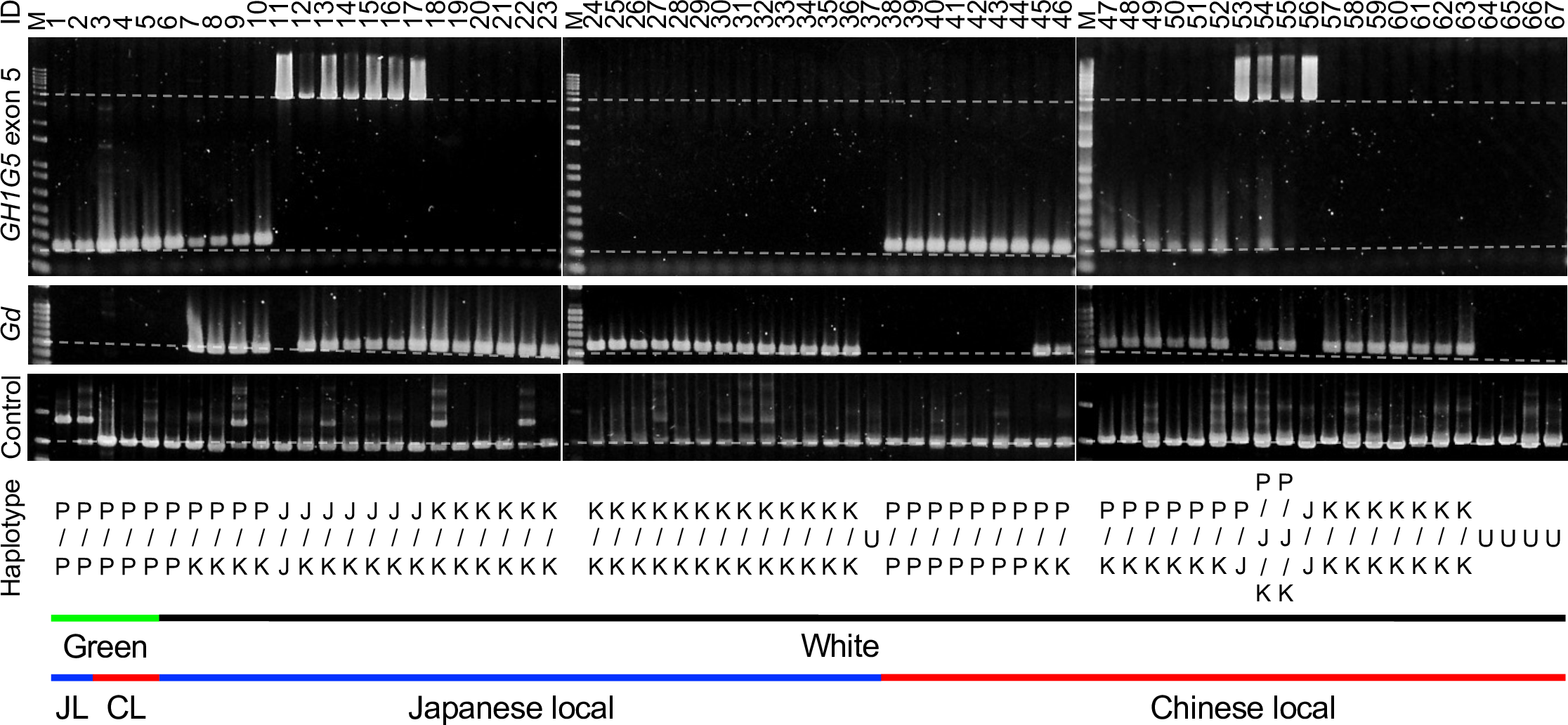

Supplement: S10 Fig — Haplotypes of the strains were determined by PCR genotyping. P/P, only the p50T genotype was detected; P/J/K, all p50T, J01, and Kosetsu genotypes were detected; J/J, only the J01 genotype was detected; J/K, both the J01 and Kosetsu genotypes were detected; K/K, only the Kosetsu genotype was detected; U, unknown, nothing detected. The primer set amplifying the genomic region from exons 1 to 2 of KWMTBOMO14639 (rp49) was used as a control (predicted fragment length = 1548 bp). The straight grey dotted lines connect band markers of known size (M) which were applied at both ends of the samples. These lines represent sizes of 4000, 200, 500, and 1500 bp, from top to bottom. “JL” and “CL” stand for “Japanese local” and “Chinese local”. The colors indicate the color of the cocoons. (TIF) [file pgen.1011118.s010.tif]
