# Supplementary material for: A major endogenous glycoside hydrolase mediating quercetin uptake in Bombyx mori
Source: PLoS Genet. 2024 Jan 17;20(1):e1011118. doi: 10.1371/journal.pgen.1011118 (PMC10824415; doi:10.1371/journal.pgen.1011118)
Supplement: Attachment — Submitted filename: response_to_reviewers.pdf [file pgen.1011118.s020.pdf]

Responses to the reviewers' comments

We would like to thank the three reviewers sincerely for reviewing. We first summarize the primary changes we made to more easily grasp where and how we revised. Unless specifically stated otherwise, line numbers in parentheses are those of the revised manuscript with changes marked. Corrected sections are marked in red in the revised version of our manuscript.

(i) Based on the nomenclature provided by CAZy (http://www.cazy.org/Home.html) and phylogeny, we named all identified 21 glycoside hydrolase family 1 proteins in *B. mori* before describing the knockout analysis (S2 Fig) (lines 232–239). For example, we renamed LQGH1 as glycoside hydrolase family 1 group G 5 (GH1G5).

(ii) As reviewer #1 and #3 pointed out, SignalP v6.0 did not predict any transmembrane domains in GH1G5 but predicted a signal domain at the N-terminus (S3A Fig) (lines 242–256). Considering this prediction and inappropriate methodological points in comparing the homogenate and insoluble fractions in their hydrolytic activity (in Fig 3 in the previous manuscript, as pointed out by reviewer #3), we concluded that GH1G5 is not a membrane-localized protein but rather would be a secretory enzyme. We have deleted the result of transmembrane domain prediction (S4 Fig in the previous manuscript) and the data showing the hydrolytic activity of the midgut insoluble fractions on quercetin glycosides. Following these changes, we have also changed related descriptions in the manuscript (lines 419, 459, 487, 492, 787 and 893).

(iii) According to reviewer #2 suggestion, we moved the figure of the phylogenetic tree of lepidopteran-wide glycoside hydrolases including GH1G5 (S11 Fig in the previous manuscript) to the main text as Fig 4. According to this change, we made the related description in S1 text of the previous manuscript presented in the main text (lines 538–561). Because this change resulted in a major reduction in the content of S1 text, we also moved the description about sequence determination of GH1G5 in S1 text to the main text (lines 400–415). As a result, the supplementary text was deleted.

We believe comments provided by the three reviewers greatly improved our manuscript. Below, we have done a point-by-point reply to reviewer's comments. Our responses are described in blue color.

Reviewer #1: This manuscript presents a comprehensive overview of the discovery of a third locus within the silkworm that plays a crucial role in the uptake of quercetin from mulberry leaves. This specific locus consists of a cluster of genes arranged in tandem, all of which encode glycoside

hydrolase family 1 proteins. Knockout of one of these genes resulted in a noticeable decline in the uptake of quercetin, particularly of rutin. Notably, the researchers demonstrated that mutations occurring in this gene have been selected for in various silkworm strains due to their association with enhanced silk production. In my assessment, I found this paper to be highly captivating and the experiments to be very well designed. Presented below are a few comments aimed at further enhancing the quality of the paper.

Response: Thank you very much for valuing our manuscript and providing meaningful comments.

Reading through the paper, I was not really sure that the glycosidases encoded by the gene cluster on chromosome 20 were actually "glycoside hydrolase family 1 (GH1)" proteins (in line with the CAZy nomenclature). I would recommend that the authors address this point more explicitly in the revised version of their manuscript. For example on page 7, line 132, the term "beta-glycosidase family 1" is misleading and does not follow the CAZy nomenclature naming carbohydrate-active enzymes.

Response: Thank you for your important feedback. In the revised manuscript, we have clearly mentioned the classification of the proteins encoded by the nine clustered glycoside hydrolase genes located in the *Gd* locus, according to the nomenclature provided by CAZy (lines 223–225). To confirm that the clustered glycoside hydrolases belong to GH1, we used InterProScan (Zdobnov et al. 2001, reference No. 49), one of the most popular and authoritative protein classification tools for protein annotation. InterProScan provides cross-references to other databases, which includes CAZy.

On page 15, lines 217-219, I am of the opinion that the entity you identify as "a hydrophobic transmembrane domain in the first N-terminal 20 amino acid residues" could potentially represent a signal peptide. Considering the context that LQGH1 is linked to the apical membrane of midgut cells, oriented towards the exterior of the cell, the requirement for a signal peptide becomes apparent to facilitate such subcellular localization. In light of this, I recommend that the authors explore this possibility using the prediction program SignalP v6.0 (https://services.healthtech.dtu.dk/services/SignalP-6.0/), which has been optimized to more effectively discriminate between signal peptides and hydrophobic transmembrane domains.

Response: We completely agree with your opinion. This was a crucial oversight of ours and we sincerely thank you for pointing this out. We revised it as described above (lines 10–18 in this text).

I am convinced that LQGH1 plays a pivotal role in quercetin uptake through deglycosylation of quercetin glycosides. This conviction stems from the outcomes of the presented knockout experiments coupled with the hydrolysis assays. However, I found it somewhat disappointing that the authors did not attempt to express LQGH1 and the other GH1 proteins from the cluster in a heterologous system. On the other hand, considering the substantial volume of data presented in this study, I think that this absence should not necessarily hinder the acceptance of the paper for publication. I am hopeful that the inclusion of such data could be a valuable addition to a subsequent paper on this subject.

Response: Thank you for your meaningful remark. We totally agree with that analysis using a heterologous expression system would have provided more reliable information to understand the enzymatic function of GH1G5. However, we could not manage to prepare for this. We acknowledged that the results we provided did not clearly elucidate the enzymatic function of GH1G5, we have toned down the conclusions on the GH1G5 enzymatic function (lines 26, 533, 792, 899 and 909). The manuscript does not contain any assertive statements on the enzymatic function of GH1G5 throughout.

In my view, a notable strength of this paper lies in the authors' commitment to investigating the potential existence of naturally occurring mutations at the LQGH1 locus within silkworm populations extensively employed for silk production. The authors successfully identified multiple distinct mutations at the LQGH1 locus in silkworm populations from both Japan and China. Importantly, these mutations were correlated with an increased silk production. Altogether, I strongly believe that a polished version of this paper, taking into account my comments, would fit very nicely in PloS Genetics.

Response: Thank you for valuing our research. We would like to thank you again sincerely for your meaningful comments.

Minor comments:

Page 4, line 56: Replace “high” with “higher”.

Page 5, line 82: replace ”enzymic activity” with “enzymatic activity”.
Page 6, line 106: replace “gradated range” with “graduated range”.

Response: Thank you for pointing them out. We have addressed all (lines 98, 137 and 168).

Reviewer #2: (The summary of the manuscript you provided is skipped here. Thank you for the comprehensive summary you provided, demonstrating your understanding of our work) Overall I found the experimental plan and detailed design of this study to be logical, comprehensive, and complete. The data are clear and presented well in support of the authors’ hypotheses. Similarly, the Materials and Methods section appears to be complete in providing essential information and access to technically important details including critical reagents.

I also found the report to be well organized and well-written. I appreciated the authors’ skill at providing brief summaries of the nature and significance of their findings throughout it, especially given its technical breadth and complexity in terms of the number of different experimental approaches they used, and the proposed significance of their findings. Regarding the writing itself, although I found a few typos (e.g., on line 512 please change “makers” to “markers”) and minor instances where I thought English expression could be improved, at the journal’s recommendation I do not list them in this review because a professional copy editor can easily find them. I would be willing to provide a marked copy if desired.

Response: We would like to express our deep gratitude for valuing our manuscript and your assistance in improving it. The suggested revisions in English expressions you provided have been appropriately presented in the revised manuscript. We believe it has greatly enhanced our manuscripts.

Regarding the balance of ideas presented in the report, after identifying LQGH1 the authors put considerable effort and energy into characterizing mutations of the gene (or "locus” may be a more accurate term) among domesticated regional Japanese and Chinese silkworm strains which form a foundation for sericultural work. This survey turned up some interesting features, including (as already noted) the greater size (weight) of white vs green cocoon strains, suggesting the absence of

flavonoid modification pathways and/or content likely had adaptive significance during domestication and subsequent artificial breeding and selection. These data also pointed to a likelihood that other quercetin or rutin utilization pathways remain to be discovered in silkworms. Nevertheless – and this may be my bias – even though in the Discussion the authors relate these findings to questions of evolutionary conservation of flavonoid-biochemistry-related processes among insect and non-insect taxa (especially mammals), I am not sure how much interest this aspect of the report will generate outside of the silkworm or sericulture community. On the other hand, I found the phylogenetic study of glycosidase orthologs to be relevant for a potentially wide audience involved with insect (especially but not only Lepidoptera) and arthropod genomics, genetics, taxonomy, physiology, and biochemistry. Yet (again as noted) the authors placed the two figures for these data in the supplementary text, and wrote very little about it in the main text, with virtually no mention in the Discussion. Given the importance of flavonoids for insects, it seems to me the impact of this research might be enhanced by moving at least one figure for the phylogenetic study (?S11?) to the main text.

Response: Thank you very much for your critical comments. According to your suggestion, we moved the figure of the phylogenetic tree of lepidopteran-wide glycoside hydrolases including GH1G5 (S11 in the previous manuscript) to the main text as Fig 4. In addition, we showed sequence identity and similarity among the group G glycoside hydrolases in *B. mori* and those among GH1G5-class proteins in Macroheteroceran moths there. According to these changes, we made the content of S1 text presented in the main text (lines 538–561). We expect that this information will be useful for future research on insect glycoside hydrolases.

The authors may also consider adding a brief statement to the Discussion about the significance of this (and other flavonoid-pathway genes yet to be discovered?) for determining the evolution of other lepidopteran clades as they did for silkworm strains. I suggest this primarily to call their findings to the attention of phylogeneticists engaged in deciphering the Noctuidae who may find them useful models for their studies.

Response: Thank you for your suggestion. We noted that the rutin hydrolytic activity of GH1G5 is possibly related to high level in mulberry leaves and the monophagy of the silkworms, and further noted that a characterization of species-wide insect GH1 proteins may provide important insights into dietary habits of herbivorous insects (lines 1033–1047).

Once again, thank you for your invaluable assistance in improving our manuscript.

Reviewer #3: The manuscript describes the discovery of a b-glucosidase that is involved in the sequestration of plant flavonoids in cocoons of the silkworm Bombyx mori. This study combined forward and reverse genetic approaches with chemical and biochemical analyses resulting in an impressive dataset. The results support the hypothesis that deglycosylation of flavonoid glycosides (especially rutin) by LQGH1, is essential step for their uptake probably via passive absorption of the hydrolysis product quercetin. While I really enjoyed reading the study, I have major concerns about the in silico amino acid sequence analysis, enzyme assays, the lack of LQGH1 sequence verification from both strains using Sanger sequencing, and conclusions on the localization and the role of unequal crossing over in generating the different haplotypes described in this study. Once these concerns are addressed, this study will be an excellent contribution to the field of molecular insect-plant interactions and the versatile roles of b-glucosidases in insects. The authors strongly emphasize their findings in the context of silkworm domestication and breeding, which I think could be a bit more balanced in the discussion with regard to implications for insect-plant coevolutionary interactions.

Response: Thank you very much for comprehensive reviewing our manuscript and providing meaningful comments. We believe your comments have greatly improved our manuscript. According to your comments, we added or retracted some results. Furthermore, with attention placed on the limitation of inference provided by our results, we majorly revised the discussion.

My detailed comments and suggestions are listed below:

Abstract l 19: “here we identified and characterized a glycosidase”: this statement implies that the enzyme was heterologously expressed and biochemically characterized. As this was not done, please rephrase. In general, to account for the fact that the enzyme was not heterologously expressed and characterized, please tone down the conclusions on the function.

Response: Thank you for pointing that out. We have corrected it (lines 23–25). We have toned down the conclusions on the GH1G5 enzymatic function (lines 26, 533, 792, 899 and 909). The manuscript does not contain any assertive statements on the enzymatic function of GH1G5 throughout.

Abstract l 24: “unequal crossing over hotspot”: I have difficulties to follow the conclusion that all the different variants result from unequal crossing over. Are there no other possible explanations?

Response: Thank you for pointing that out. We agree with that the conserved defective structural mutations or past gene duplications were not necessarily caused by unequal crossing over. We have changed it (lines 28–29).

Introduction line 84: quercetin glucosidases or quercetin glycosides?

Response: In the manuscript, we used "quercetin glycosides" when it indicates quercetin glycosides including rutin. Because quercetin in the silkworm tissues or cocoon are present as that bound with glucose, here we used "quercetin glucosides".

Results line 132: LPH belong to the glycoside hydrolase (GH) family 1

Response: Thank you for pointing that out. We have corrected it (line 227).

Line 146: correct to: Malpighian tubules

Response: Thank you for pointing that out. We have corrected it (line 262).

Lines 173-175: was it possible to obtain heterozygous knock-out mutants? If yes, what was their phenotype?

Response: We were successful in obtaining heterozygous knock-out mutants of *KWMTBOMO12222* and *KWMTBOMO12236* (named as *GH1G1* and *GH1G9* in the revised manuscript). However, we did not observe any noticeable changes in their phenotypes, including cocoon color.

Results lines 205 ff: I have several major concerns regarding the sequence analysis and enzyme assays:
1) Amino acid sequence similarity between mammalian LPH and LQGH1: both proteins are predicted to belong to the GH1 family based on their amino acid sequence, so it is expected that their sequences are similar. In my opinion, this statement is unnecessary but if the authors have important reasons to keep the statement, they should at least give the percentages of sequence identity/similarity.
2) Similarly, an orthologous relationship of LPH and LQGH1 is not expected and is impossible to deduce with phylogenetic analyses as is demonstrated by the lack of statistical support for most nodes, and the fact that only gene clusters from the same species are supported at all. Similar results have been obtained in other studies on insect GH1s, which should be included in the disucssion. I think a more thorough analysis of the GH1 gene family in Bombyx (e.g., the number of predicted genes, their phylogenetic relationships and sequence similarities, in particular within the cluster that harbors LQGH1) that is then placed in the larger context of Lepidopteran GH1s as shown in Fig. S11 would be more informative and meaningful.

Response: We totally agree with your comments. We concluded that a comparison of GH1G5 and LPH sequences would not be informative and instead add information on comparisons within GH1 in Lepidoptera. According to this, we revised our manuscript as described above (lines 6-9 and 19–24 in this text). In addition, we showed sequence identity and similarity among the group G glycoside hydrolases (indicating the clustered glycoside hydrolases located in the *Gd* locus) in *B. mori* and those among GH1G5-class proteins in Macroheteroceran moths (Fig 5 and lines 551–552). In addition, we added a discussion related the sequence identities in lines 1036–1039.

3) The authors state that LQGH1 contains a hydrophobic transmembrane domain at the N-terminus. I

am unfamiliar with the tool that the authors used to predict this and I also have difficulties to interpret the graph shown in Fig. S4. Many insect GH1 are secreted proteins that contain an N-terminal secretion signal. Considering that at least some GH1s are involved in digestion, it is also expected that they are secreted into the gut lumen. I repeated the sequence analysis using several programs that are widely used to predict the presence of a signal peptide and transmembrane domains in proteins as well as protein localization (SignalP, TMHMM, DeepLoc). All these programs are available online and are free of charge. The results clearly show that the enzyme has an N-terminal secretion signal, no transmembrane domain, and is located extracellularly. It is thus very unlikely that LQGH1 is membrane-anchored and instead is secreted into the gut lumen.

Response: We completely agree with your comment. We revised our manuscript much as described in lines 10–18 in this text. This was a crucial oversight of ours and we sincerely thank you for pointing this out.

4) Based on this result I have additional concerns regarding the enzyme assays with gut homogenates. In general, the description of the methods is not sufficient. Please specify how the dissected guts were homogenized, if samples consisted of pooled guts and if the replicates are biological or technical replicates. Are homogenate and insoluble fraction samples derived from the same initial homogenate? Was the insoluble fraction dissolved again in the same volume?

Response: We apologize for the lack of explanation. We added information about the assay in lines 1328–1434. In addition, we have added a note to all relevant figure legends that the replicates are independent biological replicates. Insoluble fraction samples were derived from the same initial homogenate. However, the insoluble fraction was not dissolved again in the same volume of boiled soluble fraction of the homogenate. As described above (lines 10–18 in this text), we retracted the data showing the hydrolytic activity of the midgut insoluble fractions on quercetin glycosides. Thank you for providing crucial comments.

5) In case of the enzyme assays the authors are missing an important background control, the substrate itself. Instead of using protein without substrate, boiled protein with substrate should be used, or at least controls without protein but including the substrate. This is an important control because very

often you will find some contamination of the substrate. Were the assays carried out with a substrate mixture or individual substrates? Why was the insoluble fraction not tested for the mutant GD2?

Response: The assays were carried out independently with each of the three substrates, rutin, Q3MG and Q3G. We revised the explanation on lines 1334−1335. The enzyme-independent degradation of the quercetin glycosides and quercetin aglycone in the reaction was examined not as experimental controls but as prerequisites for this assay. We added the explanations on lines 1336–1431. The glycosides and aglycone have been confirmed to be stable in the condition. The reason for lacking the test using insoluble fraction of the mutant *Gd2* was a shortage of a substrate Q3MG.

6) As explained above, the comparison of homogenate with the insoluble fraction is not appropriate. Instead, the homogenate should be separated into soluble and insoluble fraction for enzyme assays.

Response: We totally agree with you. As described above and below (lines 10–18 in this text), we retracted the data showing the hydrolytic activity of the midgut insoluble fractions on quercetin glycosides (Fig 3B in the previous manuscript).

Fig. 3: The structures of rutin and Q3G are wrong and have to be corrected. Please carefully check all structures again.

Response: Thank you for pointing that out. We have corrected them (Figure 3A).

Line 300: two sites within exon 5?

Response: We apologize for the confusing explanation. The 3997 bp insertion shifted the splice site of the exon 5 end 123 bp forward in J01 GH1G5 (Fig 5A). For better explanations, we revised the figures and the manuscript (Fig 5A, S8A Fig and Lines 610–656). Additionally, in the revision, we identified the inserted 3997 bp sequence as the reported non-autonomous transposable element in *B. mori*, *Neet* (lines 610–614). The inversion and duplication of the intron 5 sequence of p50T *GH1G5* were presumed to be occurred after the transposable element insertion.

Line 303: It is not really clear how the insertion of intron sequences into an exon leads to a protein sequence that lacks 31 amino acids. Can this be shown graphically?

Response: For better explanations, we revised the figures and the manuscript (Fig 5A and Lines 614–656). Thank you for your suggestion. In addition, the deletion length of amino acids, 31, was a typing error and has been corrected to 41. We apologize again.

The difference in amino acid sequence should also be confirmed by amplifying and Sanger-sequencing the full-length sequence from cDNAs from both strains. If I understand correctly, the sequence was only confirmed in strain p50T based on RNA-Seq data but not in the J01 strain.

Response: We tried to determine full-length sequences of cDNA of p50T and J01 GH1G5 transcripts by 5′- and 3′-RACE PCR, but were unsuccessful in cloning a region including their 5'UTR. As an alternative way, we determined full-length sequences of their ORF by PCR which amplify the putative longest open reading frame of *GH1G5*. It is detailly explained in lines 404–407, 657–659 and 1290–1316, and S6A Fig and S8C Fig.

Line 328: what exactly is meant with “weaker signal”?

Response: It means that the RT-PCR indicated the relative low expression of *GH1G5* in J01. To clarify what it means, we have changed it (line 714).

Line 332: What is the exact evidence for the genomic changes being the result of unequal crossing over?

Response: It is based on presumption and there is no exact evidence. Thank you for pointing this out. We have changed it (line 749).

Fig. 4A: the dot plot analysis is not easy to understand. What is the significance of the different colors (green versus black)?

Response: We apologize for the confusing explanation. For better explanations, we revised the figures and the manuscript (Fig 5A and Lines 610–656) and moved the dot plot in S8A Fig. The green dots indicate inverted regions. We add the explanation to the legend (lines 2044–2045). Thank you for pointing this out.

Line 370: uptake from the midgut?

Response: Thank you for pointing this confusing description out. We have changed it (line 790).

Line 379-380: it would be indeed great if functional studies with recombinant LQGH1 and other enzymes encoded in the gene cluster could be included in this study

Response: Thank you for your suggestion. We totally agree that it would enhance a significance of our study. However, we could not manage to prepare for the experiment. This is an interesting investigation that we would like to address in future analyses. Relevant discussions were added to the revised manuscript (lines 1039–1047).

Line 395-396: I would be more careful with the conclusion that LQGH1 has conserved rutin hydrolytic activity, I don't think that the available data are sufficient to support this conclusion

Response: We totally agree with your comment. We did not investigate the enzymatic activities of putative Macroheteroceran orthologs of GH1G5. What we tried to mean was that GH1G5 of *B.mori* has retained the rutin hydrolytic activity. We understand that what our results lead are the following three; (i) GH1G5 of *B.mori* is suggested to have hydrolytic activity on rutin, Q3MG and Q3G. (ii) The hydrolytic activity is suggested to be involved in quercetin uptake in *B. mori*. (iii) The phylogeny suggests that GH1G5 had evolved through gene duplication after the divergence of Pyraloidea and Macroheterocera. The manuscript has been revised, with attention placed on this limitation of a rational inference (lines 897–1047). Thank you for pointing this out.

Line 401: why is it a peculiar function? Consider to rephrase.

Response: Thank you for pointing this out. It has been deleted in revising the whole paragraph.

Line 417-418: please rephrase, "crisis of loss". Again, I am not convinced that all the variants are explained by unequal crossing over.

Response: We agree with that the conserved defective structural mutations or past gene duplications were not necessarily caused by unequal crossing over. We have changed it (line 982).

Line 418-419: What is meant by “remained conserved”? The enzymatic activity of putative orthologs has not been tested. Please tone down the conclusions.

Response: What we tried to mean was that GH1G5 of the mulberry silkworms has retained the rutin hydrolytic activity. It has been changed (lines 982−983). Thank you for pointing this out.

Line 425: I think it would helpful to have a cartoon showing the proposed steps in quercetin glycoside metabolism, transport, and excretion in Bombyx

Response: Thank you for your suggestion. We add a diagram illustrating a proposed model for quercetin uptake in the silkworm midgut, which is directly related with our presented results (Fig 6).

We are most grateful to you for providing comments which have greatly improved our manuscript and provided us a helpful learning opportunity.
