# Supplementary material for: A major endogenous glycoside hydrolase mediating quercetin uptake in Bombyx mori
Source: PLoS Genet. 2024 Jan 17;20(1):e1011118. doi: 10.1371/journal.pgen.1011118 (PMC10824415; doi:10.1371/journal.pgen.1011118)
Supplement: Attachment — Submitted filename: PGENETICS-D-23-00886_R1-2 workingcopy MRG.pdf [file pgen.1011118.s021.pdf]

# PLOS Genetics

## A major endogenous glycoside hydrolase mediating quercetin uptake in <i>Bombyx mori</i>

--Manuscript Draft--

| | |
|---|---|
| **Manuscript Number:** | PGENETICS-D-23-00886R1 |
| **Full Title:** | A major endogenous glycoside hydrolase mediating quercetin uptake in <i>Bombyx mori</i> |
| **Short Title:** | GH1G5 mediates quercetin uptake in <i>Bombyx mori</i> |
| **Article Type:** | Research Article |
| **Section/Category:** | General |
| **Keywords:** | <i>Bombyx mori</i>; domestic silkworm; flavonoid metabolism; quercetin; glycosidase; breeding; cocoon; rutinosidase; glycoside hydrolase; GH1 |
| **Abstract:** | Quercetin is a common plant flavonoid which is involved in herbivore–plant interactions. Mulberry silkworms (domestic silkworm, <i>Bombyx mori<\i>, and wild silkworm, <i>Bombyx mandarina<\i>) take up quercetin from mulberry leaves and accumulate the metabolites in the cocoon, thereby improving its protective properties. Here we identified a glycoside hydrolase, named glycoside hydrolase family 1 group G 5 (GH1G5), which is expressed in the midgut and is involved in quercetin metabolism in the domestic silkworm. Our results suggest that this enzyme mediates quercetin uptake by deglycosylating the three primary quercetin glycosides present in mulberry leaf: rutin, quercetin-3-<i>O<\i>-malonyl-glucoside, quercetin-3-<i>O<\i>-glucoside. Despite being located in an instable genomic region that has undergone frequent structural changes in the evolution of Lepidoptera, GH1G5 has retained its hydrolytic activity, suggesting quercetin uptake has adaptive significance for the mulberry silkworms. <i>GH1G5<\i> is also important in breeding: defective mutations which result in discoloration of the cocoon and increased silk yield are homozygously conserved in 27 of the 32 Japanese white-cocoon domestic silkworm strains and 12 of the 30 Chinese ones we investigated. |
| **Additional Information:** | |
| **Question** | **Response** |
| **Financial Disclosure**<br><br>Enter a financial disclosure statement that describes the sources of funding for the work included in this submission and the role the funder(s) played. This includes grants and any commercial funding of the work or authors.<br><br>This statement will be typeset if the manuscript is accepted for publication.<br><br>Review the submission guidelines and the instructions link below for detailed requirements and guidance. | This work was financially supported by grants from the Japanese Ministry of Agriculture, Forestry and Fisheries, and JSPS KAKENHI Grant Numbers 21H03831 to K.Y. and H.S.. The funders had no role in study design, data collection and analysis, decision to publish, or preparation of the manuscript. |
| **Government Employee** | No - No authors are employees of the U.S. government. |

*Powered by Editorial Manager® and ProduXion Manager® from Aries Systems Corporation*

| | |
|---|---|
| Are you or any of the contributing authors an employee of the United States government?<br><br>Manuscripts authored by one or more US Government employees are not copyrighted, but are licensed under a CC0 Public Domain Dedication, which allows unlimited distribution and reuse of the article for any lawful purpose. This is a legal requirement for US Government employees.<br><br>This will be typeset if the manuscript is accepted for publication. | |
| **Competing Interests**<br><br>On behalf of all authors, disclose any competing interests that could be perceived to bias this work.<br><br>This statement will be typeset if the manuscript is accepted for publication.<br><br>Review the instructions link below and PLOS Genetics' competing interests policy to determine what information must be disclosed at submission. | The authors declare no competing interests. |
| **Data Availability**<br><br>Provide a **Data Availability Statement** in the box below. This statement should detail where the data used in this submission can be accessed. This statement will be typeset if the manuscript is accepted for publication.<br><br>Before publication, authors are required to make all data underlying their findings fully available, without restriction. Review our PLOS Data Policy page for detailed information on this policy. Instructions for writing your Data Availability statement can be accessed via the Instructions link | The raw genomic sequencing data of the Kosetsu strain are available in the Sequence Read Archive under the BioProject accession number PRJDB15366. |

| | |
|---|---|
| below. | |
| **Financial Disclosure**<br><br>Enter a financial disclosure statement that describes the sources of funding for the work included in this submission. Review the submission guidelines for detailed requirements. View published research articles from *PLOS Genetics* for specific examples.<br><br>This statement is required for submission and **will appear in the published article** if the submission is accepted. Please make sure it is accurate.<br><br>**Funded studies**<br>Enter a statement with the following details:<br>• Initials of the authors who received each award<br>• Grant numbers awarded to each author<br>• The full name of each funder<br>• URL of each funder website<br>• Did the sponsors or funders play any role in the study design, data collection and analysis, decision to publish, or preparation of the manuscript?<br><br>Did you receive funding for this work? | Yes |
| Please add funding details.<br>as follow-up to "**Financial Disclosure**<br><br>Enter a financial disclosure statement that describes the sources of funding for the work included in this submission. Review the submission guidelines for detailed requirements. View published research articles from *PLOS Genetics* for specific examples.<br><br>This statement is required for submission and **will appear in the published article** if the submission is accepted. Please make sure it is accurate. | This work was supported by MAFF Commissioned project study on "Research project for sericultural bio-industry" Grant Number JP22680575 to R.W., T.I. and S.T., and JSPS KAKENHI Grant Numbers 21H03831 to K.Y. and H.S.. The funders had no role in study design, data collection and analysis, decision to publish, or preparation of the manuscript. |

| | |
|---|---|
| **Funded studies**<br>Enter a statement with the following details:<br>• Initials of the authors who received each award<br>• Grant numbers awarded to each author<br>• The full name of each funder<br>• URL of each funder website<br>• Did the sponsors or funders play any role in the study design, data collection and analysis, decision to publish, or preparation of the manuscript?<br><br>Did you receive funding for this work?" | |
| Please select the country of your main research funder (please select carefully as in some cases this is used in fee calculation).<br>as follow-up to "**Financial Disclosure**<br><br>Enter a financial disclosure statement that describes the sources of funding for the work included in this submission. Review the submission guidelines for detailed requirements. View published research articles from *PLOS Genetics* for specific examples.<br><br>This statement is required for submission and **will appear in the published article** if the submission is accepted. Please make sure it is accurate.<br><br>**Funded studies**<br>Enter a statement with the following details:<br>• Initials of the authors who received each award<br>• Grant numbers awarded to each author<br>• The full name of each funder<br>• URL of each funder website<br>• Did the sponsors or funders play any role in the study design, data collection and analysis, decision to publish, or preparation of the manuscript?<br><br>Did you receive funding for this work?" | JAPAN - JP |

**<u>Responses to the reviewers' comments</u>**

We would like to thank the three reviewers sincerely for reviewing. We first summarize the primary changes we made to more easily grasp where and how we revised. Unless specifically stated otherwise, line numbers in parentheses are those of the revised manuscript with changes marked. Corrected sections are marked in red in the revised version of our manuscript.

(i) Based on the nomenclature provided by CAZy (http://www.cazy.org/Home.html) and phylogeny, we named all identified 21 glycoside hydrolase family 1 proteins in *B. mori* before describing the knockout analysis (S2 Fig) (lines 232–239). For example, we renamed LQGH1 as glycoside hydrolase family 1 group G 5 (GH1G5).

(ii) As reviewer #1 and #3 pointed out, SignalP v6.0 did not predict any transmembrane domains in GH1G5 but predicted a signal domain at the N-terminus (S3A Fig) (lines 242–256). Considering this prediction and inappropriate methodological points in comparing the homogenate and insoluble fractions in their hydrolytic activity (in Fig 3 in the previous manuscript, as pointed out by reviewer #3), we concluded that GH1G5 is not a membrane-localized protein but rather would be a secretory enzyme. We have deleted the result of transmembrane domain prediction (S4 Fig in the previous manuscript) and the data showing the hydrolytic activity of the midgut insoluble fractions on quercetin glycosides. Following these changes, we have also changed related descriptions in the manuscript (lines 419, 459, 487, 492, 787 and 893).

(iii) According to reviewer #2 suggestion, we moved the figure of the phylogenetic tree of lepidopteran-wide glycoside hydrolases including GH1G5 (S11 Fig in the previous manuscript) to the main text as Fig 4. According to this change, we made the related description in S1 text of the previous manuscript presented in the main text (lines 538–561). Because this change resulted in a major reduction in the content of S1 text, we also moved the description about sequence determination of GH1G5 in S1 text to the main text (lines 400–415). As a result, the supplementary text was deleted.

We believe comments provided by the three reviewers greatly improved our manuscript. Below, we have done a point-by-point reply to reviewer's comments. Our responses are described in blue color.

**Reviewer #1:** This manuscript presents a comprehensive overview of the discovery of a third locus within the silkworm that plays a crucial role in the uptake of quercetin from mulberry leaves. This specific locus consists of a cluster of genes arranged in tandem, all of which encode glycoside

hydrolase family 1 proteins. Knockout of one of these genes resulted in a noticeable decline in the uptake of quercetin, particularly of rutin. Notably, the researchers demonstrated that mutations occurring in this gene have been selected for in various silkworm strains due to their association with enhanced silk production. In my assessment, I found this paper to be highly captivating and the experiments to be very well designed. Presented below are a few comments aimed at further enhancing the quality of the paper.

Response: Thank you very much for valuing our manuscript and providing meaningful comments.

Reading through the paper, I was not really sure that the glycosidases encoded by the gene cluster on chromosome 20 were actually “glycoside hydrolase family 1 (GH1)” proteins (in line with the CAZy nomenclature). I would recommend that the authors address this point more explicitly in the revised version of their manuscript. For example on page 7, line 132, the term “beta-glycosidase family 1” is misleading and does not follow the CAZy nomenclature naming carbohydrate-active enzymes.

Response: Thank you for your important feedback. In the revised manuscript, we have clearly mentioned the classification of the proteins encoded by the nine clustered glycoside hydrolase genes located in the *Gd* locus, according to the nomenclature provided by CAZy (lines 223–225). To confirm that the clustered glycoside hydrolases belong to GH1, we used InterProScan (Zdobnov et al. 2001, reference No. 49), one of the most popular and authoritative protein classification tools for protein annotation. InterProScan provides cross-references to other databases, which includes CAZy.

On page 15, lines 217-219, I am of the opinion that the entity you identify as "a hydrophobic transmembrane domain in the first N-terminal 20 amino acid residues" could potentially represent a signal peptide. Considering the context that LQGH1 is linked to the apical membrane of midgut cells, oriented towards the exterior of the cell, the requirement for a signal peptide becomes apparent to facilitate such subcellular localization. In light of this, I recommend that the authors explore this possibility using the prediction program SignalP v6.0 (https://services.healthtech.dtu.dk/services/SignalP-6.0/), which has been optimized to more effectively discriminate between signal peptides and hydrophobic transmembrane domains.

Response: We completely agree with your opinion. This was a crucial oversight of ours and we sincerely thank you for pointing this out. We revised it as described above (lines 10–18 in this text).

I am convinced that LQGH1 plays a pivotal role in quercetin uptake through deglycosylation of quercetin glycosides. This conviction stems from the outcomes of the presented knockout experiments coupled with the hydrolysis assays. However, I found it somewhat disappointing that the authors did not attempt to express LQGH1 and the other GH1 proteins from the cluster in a heterologous system. On the other hand, considering the substantial volume of data presented in this study, I think that this absence should not necessarily hinder the acceptance of the paper for publication. I am hopeful that the inclusion of such data could be a valuable addition to a subsequent paper on this subject.

Response: Thank you for your meaningful remark. We totally agree with that analysis using a heterologous expression system would have provided more reliable information to understand the enzymatic function of GH1G5. However, we could not manage to prepare for this. We acknowledged that the results we provided did not clearly elucidate the enzymatic function of GH1G5, we have toned down the conclusions on the GH1G5 enzymatic function (lines 26, 533, 792, 899 and 909). The manuscript does not contain any assertive statements on the enzymatic function of GH1G5 throughout.

In my view, a notable strength of this paper lies in the authors' commitment to investigating the potential existence of naturally occurring mutations at the LQGH1 locus within silkworm populations extensively employed for silk production. The authors successfully identified multiple distinct mutations at the LQGH1 locus in silkworm populations from both Japan and China. Importantly, these mutations were correlated with an increased silk production. Altogether, I strongly believe that a polished version of this paper, taking into account my comments, would fit very nicely in PloS Genetics.

Response: Thank you for valuing our research. We would like to thank you again sincerely for your meaningful comments.

Minor comments:

Page 4, line 56: Replace “high” with “higher”.

Page 5, line 82: replace ”enzymic activity” with “enzymatic activity”.
Page 6, line 106: replace “gradated range” with “graduated range”.

Response: Thank you for pointing them out. We have addressed all (lines 98, 137 and 168).

**Reviewer #2:** (The summary of the manuscript you provided is skipped here. Thank you for the comprehensive summary you provided, demonstrating your understanding of our work) Overall I found the experimental plan and detailed design of this study to be logical, comprehensive, and complete. The data are clear and presented well in support of the authors’ hypotheses. Similarly, the Materials and Methods section appears to be complete in providing essential information and access to technically important details including critical reagents.

I also found the report to be well organized and well-written. I appreciated the authors’ skill at providing brief summaries of the nature and significance of their findings throughout it, especially given its technical breadth and complexity in terms of the number of different experimental approaches they used, and the proposed significance of their findings. Regarding the writing itself, although I found a few typos (e.g., on line 512 please change “makers” to “markers”) and minor instances where I thought English expression could be improved, at the journal’s recommendation I do not list them in this review because a professional copy editor can easily find them. I would be willing to provide a marked copy if desired.

Response: We would like to express our deep gratitude for valuing our manuscript and your assistance in improving it. The suggested revisions in English expressions you provided have been appropriately presented in the revised manuscript. We believe it has greatly enhanced our manuscripts.

Regarding the balance of ideas presented in the report, after identifying LQGH1 the authors put considerable effort and energy into characterizing mutations of the gene (or "locus” may be a more accurate term) among domesticated regional Japanese and Chinese silkworm strains which form a foundation for sericultural work. This survey turned up some interesting features, including (as already noted) the greater size (weight) of white vs green cocoon strains, suggesting the absence of

flavonoid modification pathways and/or content likely had adaptive significance during domestication and subsequent artificial breeding and selection. These data also pointed to a likelihood that other quercetin or rutin utilization pathways remain to be discovered in silkworms. Nevertheless – and this may be my bias – even though in the Discussion the authors relate these findings to questions of evolutionary conservation of flavonoid-biochemistry-related processes among insect and non-insect taxa (especially mammals), I am not sure how much interest this aspect of the report will generate outside of the silkworm or sericulture community. On the other hand, I found the phylogenetic study of glycosidase orthologs to be relevant for a potentially wide audience involved with insect (especially but not only Lepidoptera) and arthropod genomics, genetics, taxonomy, physiology, and biochemistry. Yet (again as noted) the authors placed the two figures for these data in the supplementary text, and wrote very little about it in the main text, with virtually no mention in the Discussion. Given the importance of flavonoids for insects, it seems to me the impact of this research might be enhanced by moving at least one figure for the phylogenetic study (?S11?) to the main text.

Response: Thank you very much for your critical comments. According to your suggestion, we moved the figure of the phylogenetic tree of lepidopteran-wide glycoside hydrolases including GH1G5 (S11 in the previous manuscript) to the main text as Fig 4. In addition, we showed sequence identity and similarity among the group G glycoside hydrolases in *B. mori* and those among GH1G5-class proteins in Macroheteroceran moths there. According to these changes, we made the content of S1 text presented in the main text (lines 538–561). We expect that this information will be useful for future research on insect glycoside hydrolases.

The authors may also consider adding a brief statement to the Discussion about the significance of this (and other flavonoid-pathway genes yet to be discovered?) for determining the evolution of other lepidopteran clades as they did for silkworm strains. I suggest this primarily to call their findings to the attention of phylogeneticists engaged in deciphering the Noctuidae who may find them useful models for their studies.

Response: Thank you for your suggestion. We noted that the rutin hydrolytic activity of GH1G5 is possibly related to high level in mulberry leaves and the monophagy of the silkworms, and further noted that a characterization of species-wide insect GH1 proteins may provide important insights into dietary habits of herbivorous insects (lines 1033–1047).

Once again, thank you for your invaluable assistance in improving our manuscript.

**Reviewer #3:** The manuscript describes the discovery of a b-glucosidase that is involved in the sequestration of plant flavonoids in cocoons of the silkworm Bombyx mori. This study combined forward and reverse genetic approaches with chemical and biochemical analyses resulting in an impressive dataset. The results support the hypothesis that deglycosylation of flavonoid glycosides (especially rutin) by LQGH1, is essential step for their uptake probably via passive absorption of the hydrolysis product quercetin. While I really enjoyed reading the study, I have major concerns about the in silico amino acid sequence analysis, enzyme assays, the lack of LQGH1 sequence verification from both strains using Sanger sequencing, and conclusions on the localization and the role of unequal crossing over in generating the different haplotypes described in this study. Once these concerns are addressed, this study will be an excellent contribution to the field of molecular insect-plant interactions and the versatile roles of b-glucosidases in insects. The authors strongly emphasize their findings in the context of silkworm domestication and breeding, which I think could be a bit more balanced in the discussion with regard to implications for insect-plant coevolutionary interactions.

Response: Thank you very much for comprehensive reviewing our manuscript and providing meaningful comments. We believe your comments have greatly improved our manuscript. According to your comments, we added or retracted some results. Furthermore, with attention placed on the limitation of inference provided by our results, we majorly revised the discussion.

My detailed comments and suggestions are listed below:

Abstract l 19: “here we identified and characterized a glycosidase”: this statement implies that the enzyme was heterologously expressed and biochemically characterized. As this was not done, please rephrase. In general, to account for the fact that the enzyme was not heterologously expressed and characterized, please tone down the conclusions on the function.

Response: Thank you for pointing that out. We have corrected it (lines 23–25). We have toned down the conclusions on the GH1G5 enzymatic function (lines 26, 533, 792, 899 and 909). The manuscript does not contain any assertive statements on the enzymatic function of GH1G5 throughout.

Abstract l 24: "unequal crossing over hotspot": I have difficulties to follow the conclusion that all the different variants result from unequal crossing over. Are there no other possible explanations?

Response: Thank you for pointing that out. We agree with that the conserved defective structural mutations or past gene duplications were not necessarily caused by unequal crossing over. We have changed it (lines 28–29).

Introduction line 84: quercetin glucosidases or quercetin glycosides?

Response: In the manuscript, we used "quercetin glycosides" when it indicates quercetin glycosides including rutin. Because quercetin in the silkworm tissues or cocoon are present as that bound with glucose, here we used "quercetin glucosides".

Results line 132: LPH belong to the glycoside hydrolase (GH) family 1

Response: Thank you for pointing that out. We have corrected it (line 227).

Line 146: correct to: Malpighian tubules

Response: Thank you for pointing that out. We have corrected it (line 262).

Lines 173-175: was it possible to obtain heterozygous knock-out mutants? If yes, what was their phenotype?

Response: We were successful in obtaining heterozygous knock-out mutants of *KWMTBOMO12222* and *KWMTBOMO12236* (named as *GH1G1* and *GH1G9* in the revised manuscript). However, we did not observe any noticeable changes in their phenotypes, including cocoon color.

Results lines 205 ff: I have several major concerns regarding the sequence analysis and enzyme assays:

1) Amino acid sequence similarity between mammalian LPH and LQGH1: both proteins are predicted to belong to the GH1 family based on their amino acid sequence, so it is expected that their sequences are similar. In my opinion, this statement is unnecessary but if the authors have important reasons to keep the statement, they should at least give the percentages of sequence identity/similarity.

2) Similarly, an orthologous relationship of LPH and LQGH1 is not expected and is impossible to deduce with phylogenetic analyses as is demonstrated by the lack of statistical support for most nodes, and the fact that only gene clusters from the same species are supported at all. Similar results have been obtained in other studies on insect GH1s, which should be included in the disucssion. I think a more thorough analysis of the GH1 gene family in Bombyx (e.g., the number of predicted genes, their phylogenetic relationships and sequence similarities, in particular within the cluster that harbors LQGH1) that is then placed in the larger context of Lepidopteran GH1s as shown in Fig. S11 would be more informative and meaningful.

Response: We totally agree with your comments. We concluded that a comparison of GH1G5 and LPH sequences would not be informative and instead add information on comparisons within GH1 in Lepidoptera. According to this, we revised our manuscript as described above (lines 6-9 and 19–24 in this text). In addition, we showed sequence identity and similarity among the group G glycoside hydrolases (indicating the clustered glycoside hydrolases located in the *Gd* locus) in *B. mori* and those among GH1G5-class proteins in Macroheteroceran moths (Fig 5 and lines 551–552). In addition, we added a discussion related the sequence identities in lines 1036–1039.

3) The authors state that LQGH1 contains a hydrophobic transmembrane domain at the N-terminus. I

am unfamiliar with the tool that the authors used to predict this and I also have difficulties to interpret the graph shown in Fig. S4. Many insect GH1 are secreted proteins that contain an N-terminal secretion signal. Considering that at least some GH1s are involved in digestion, it is also expected that they are secreted into the gut lumen. I repeated the sequence analysis using several programs that are widely used to predict the presence of a signal peptide and transmembrane domains in proteins as well as protein localization (SignalP, TMHMM, DeepLoc). All these programs are available online and are free of charge. The results clearly show that the enzyme has an N-terminal secretion signal, no transmembrane domain, and is located extracellularly. It is thus very unlikely that LQGH1 is membrane-anchored and instead is secreted into the gut lumen.

Response: We completely agree with your comment. We revised our manuscript much as described in lines 10–18 in this text. This was a crucial oversight of ours and we sincerely thank you for pointing this out.

4) Based on this result I have additional concerns regarding the enzyme assays with gut homogenates. In general, the description of the methods is not sufficient. Please specify how the dissected guts were homogenized, if samples consisted of pooled guts and if the replicates are biological or technical replicates. Are homogenate and insoluble fraction samples derived from the same initial homogenate? Was the insoluble fraction dissolved again in the same volume?

Response: We apologize for the lack of explanation. We added information about the assay in lines 1328–1434. In addition, we have added a note to all relevant figure legends that the replicates are independent biological replicates. Insoluble fraction samples were derived from the same initial homogenate. However, the insoluble fraction was not dissolved again in the same volume of boiled soluble fraction of the homogenate. As described above (lines 10–18 in this text), we retracted the data showing the hydrolytic activity of the midgut insoluble fractions on quercetin glycosides. Thank you for providing crucial comments.

5) In case of the enzyme assays the authors are missing an important background control, the substrate itself. Instead of using protein without substrate, boiled protein with substrate should be used, or at least controls without protein but including the substrate. This is an important control because very

often you will find some contamination of the substrate. Were the assays carried out with a substrate mixture or individual substrates? Why was the insoluble fraction not tested for the mutant GD2?

Response: The assays were carried out independently with each of the three substrates, rutin, Q3MG and Q3G. We revised the explanation on lines 1334−1335. The enzyme-independent degradation of the quercetin glycosides and quercetin aglycone in the reaction was examined not as experimental controls but as prerequisites for this assay. We added the explanations on lines 1336–1431. The glycosides and aglycone have been confirmed to be stable in the condition. The reason for lacking the test using insoluble fraction of the mutant *Gd2* was a shortage of a substrate Q3MG.

6) As explained above, the comparison of homogenate with the insoluble fraction is not appropriate. Instead, the homogenate should be separated into soluble and insoluble fraction for enzyme assays.

Response: We totally agree with you. As described above and below (lines 10–18 in this text), we retracted the data showing the hydrolytic activity of the midgut insoluble fractions on quercetin glycosides (Fig 3B in the previous manuscript).

Fig. 3: The structures of rutin and Q3G are wrong and have to be corrected. Please carefully check all structures again.

Response: Thank you for pointing that out. We have corrected them (Figure 3A).

Line 300: two sites within exon 5?

Response: We apologize for the confusing explanation. The 3997 bp insertion shifted the splice site of the exon 5 end 123 bp forward in J01 GH1G5 (Fig 5A). For better explanations, we revised the figures and the manuscript (Fig 5A, S8A Fig and Lines 610–656). Additionally, in the revision, we identified the inserted 3997 bp sequence as the reported non-autonomous transposable element in *B. mori*, *Neet* (lines 610–614). The inversion and duplication of the intron 5 sequence of p50T *GH1G5* were presumed to be occurred after the transposable element insertion.

Line 303: It is not really clear how the insertion of intron sequences into an exon leads to a protein sequence that lacks 31 amino acids. Can this be shown graphically?

Response: For better explanations, we revised the figures and the manuscript (Fig 5A and Lines 614–656). Thank you for your suggestion. In addition, the deletion length of amino acids, 31, was a typing error and has been corrected to 41. We apologize again.

The difference in amino acid sequence should also be confirmed by amplifying and Sanger-sequencing the full-length sequence from cDNAs from both strains. If I understand correctly, the sequence was only confirmed in strain p50T based on RNA-Seq data but not in the J01 strain.

Response: We tried to determine full-length sequences of cDNA of p50T and J01 GH1G5 transcripts by 5′- and 3′-RACE PCR, but were unsuccessful in cloning a region including their 5'UTR. As an alternative way, we determined full-length sequences of their ORF by PCR which amplify the putative longest open reading frame of *GH1G5*. It is detailly explained in lines 404–407, 657–659 and 1290–1316, and S6A Fig and S8C Fig.

Line 328: what exactly is meant with "weaker signal"?

Response: It means that the RT-PCR indicated the relative low expression of *GH1G5* in J01. To clarify what it means, we have changed it (line 714).

Line 332: What is the exact evidence for the genomic changes being the result of unequal crossing over?

Response: It is based on presumption and there is no exact evidence. Thank you for pointing this out. We have changed it (line 749).

Fig. 4A: the dot plot analysis is not easy to understand. What is the significance of the different colors (green versus black)?

Response: We apologize for the confusing explanation. For better explanations, we revised the figures and the manuscript (Fig 5A and Lines 610–656) and moved the dot plot in S8A Fig. The green dots indicate inverted regions. We add the explanation to the legend (lines 2044–2045). Thank you for pointing this out.

Line 370: uptake from the midgut?

Response: Thank you for pointing this confusing description out. We have changed it (line 790).

Line 379-380: it would be indeed great if functional studies with recombinant LQGH1 and other enzymes encoded in the gene cluster could be included in this study

Response: Thank you for your suggestion. We totally agree that it would enhance a significance of our study. However, we could not manage to prepare for the experiment. This is an interesting investigation that we would like to address in future analyses. Relevant discussions were added to the revised manuscript (lines 1039–1047).

Line 395-396: I would be more careful with the conclusion that LQGH1 has conserved rutin hydrolytic activity, I don't think that the available data are sufficient to support this conclusion

Response: We totally agree with your comment. We did not investigate the enzymatic activities of putative Macroheteroceran orthologs of GH1G5. What we tried to mean was that GH1G5 of *B.mori* has retained the rutin hydrolytic activity. We understand that what our results lead are the following three; (i) GH1G5 of *B.mori* is suggested to have hydrolytic activity on rutin, Q3MG and Q3G. (ii) The hydrolytic activity is suggested to be involved in quercetin uptake in *B. mori*. (iii) The phylogeny suggests that GH1G5 had evolved through gene duplication after the divergence of Pyraloidea and Macroheterocera. The manuscript has been revised, with attention placed on this limitation of a rational inference (lines 897–1047). Thank you for pointing this out.

Line 401: why is it a peculiar function? Consider to rephrase.

Response: Thank you for pointing this out. It has been deleted in revising the whole paragraph.

Line 417-418: please rephrase, "crisis of loss". Again, I am not convinced that all the variants are explained by unequal crossing over.

Response: We agree with that the conserved defective structural mutations or past gene duplications were not necessarily caused by unequal crossing over. We have changed it (line 982).

Line 418-419: What is meant by “remained conserved”? The enzymatic activity of putative orthologs has not been tested. Please tone down the conclusions.

Response: What we tried to mean was that GH1G5 of the mulberry silkworms has retained the rutin hydrolytic activity. It has been changed (lines 982−983). Thank you for pointing this out.

Line 425: I think it would helpful to have a cartoon showing the proposed steps in quercetin glycoside metabolism, transport, and excretion in Bombyx

Response: Thank you for your suggestion. We add a diagram illustrating a proposed model for quercetin uptake in the silkworm midgut, which is directly related with our presented results (Fig 6).

We are most grateful to you for providing comments which have greatly improved our manuscript and provided us a helpful learning opportunity.

Manuscript (cleaned)
Click here to access/download;Manuscript;manuscript_revised_cleaned.pdf

# A major endogenous glycoside hydrolase mediating quercetin uptake in *Bombyx mori*

Short title: GH1G5 mediates quercetin uptake in *Bombyx mori*

Ryusei Waizumi*, Chikara Hirayama, Shuichiro Tomita, Tetsuya Iizuka, Seigou Kuwazaki, Akiya Jouraku, Takuya Tsubota, Kakeru Yokoi, Kimiko Yamamoto, Hideki Sezutsu

Institute of Agrobiological Sciences, National Agriculture and Food Research Organization (NARO), 1-2 Owashi, Tsukuba, Ibaraki 305-8634, Japan

*Correspondence:
Ryusei Waizumi
waizumir957@affrc.go.jp

**Abstract**

Quercetin is a common plant flavonoid which is involved in herbivore–plant interactions. Mulberry silkworms (domestic silkworm, *Bombyx mori*, and wild silkworm, *Bombyx mandarina*) take up quercetin from mulberry leaves and accumulate the metabolites in the cocoon, thereby improving its protective properties. Here we identified a glycoside hydrolase, named glycoside hydrolase family 1 group G 5 (GH1G5), which is expressed in the midgut and is involved in quercetin metabolism in the domestic silkworm. Our results suggest that this enzyme mediates quercetin uptake by deglycosylating the three primary quercetin glycosides present in mulberry leaf: rutin, quercetin-3-*O*-malonyl-glucoside, quercetin-3-*O*-glucoside. Despite being located in an instable genomic region that has undergone frequent structural changes in the evolution of Lepidoptera, GH1G5 has retained its hydrolytic activity, suggesting quercetin uptake has adaptive significance for the mulberry silkworms. *GH1G5* is also important in breeding: defective mutations which result in discoloration of the cocoon and increased silk yield are homozygously conserved in 27 of the 32 Japanese white-cocoon domestic silkworm strains and 12 of the 30 Chinese ones we investigated.

**Author summary**

Quercetin is one of the most abundant flavonoids present in plants. This flavonoid is involved in herbivorous insect–plant interactions. Insects utilize it for host–plant recognition, coloration, and protection from ultraviolet and oxidative stress. However, the molecular mechanism of quercetin metabolism in insects remains unclear. Mulberry silkworms (domestic silkworm, *Bombyx mori*, and wild silkworm, *Bombyx mandarina*) take up quercetin from mulberry leaves and sequester it into their cocoon to improve its protective properties. In this study, we identified an endogenous glycoside hydrolase in the domestic silkworm, named glycoside hydrolase family 1 group G 5 (GH1G5). This enzyme mediates quercetin uptake into the midgut cells by deglycosylating mulberry leaf-derived quercetin glycosides. This is the first discovery of a rutin glycoside hydrolase in an animal. Furthermore, we found that defective mutations of *GH1G5* have been broadly disseminated within the domestic silkworm population due to the improved cocoon color (i.e., discoloration to white) and increased silk yield. This study illuminates the unique mechanism of quercetin uptake in the domestic silkworm and uncovers an important event in the history of silkworm breeding.

## Introduction

Quercetin (3,3’,4’,5,7-pentahydroxyflavone) is a flavonoid abundantly found in a wide variety of plants [1]. Previous studies have found quercetin glycosides possess oviposition and feeding stimulant activity in lepidopteran, orthopteran and coleopteran insects [2,3,4,5,6]. These observations suggest that quercetin is widely ingested by insects. Quercetin ingestion by insects is likely not merely a consequence of the identification and feeding on host plants; it may have adaptive significance. In the common blue butterfly (*Polyommatus icarus*), which sequesters quercetin-3-*O*-galactoside in its wings, flavonoid content is higher in the female than in the male and positively correlated with female sex attraction [7,8,9]. The yellow pigment in the wings of a grasshopper (*Dissosteira carolina*), which may contribute to their camouflage in plants, results from sequestration of quercetin-3-*O*-glucoside (isoquercitrin, Q3G) [10]. Although these studies strongly emphasize the broad significance of quercetin in herbivore–plant interactions, the underlying molecular mechanisms of quercetin metabolism in insects are poorly understood.

Mulberry silkworms (domestic silkworm, *Bombyx mori*, and wild silkworm, *Bombyx mandarina*) accumulate various compounds derived from plants, including quercetin glucosides, kaempferol glucosides, and carotenoids, in their silk glands and colored cocoons (S1 Fig) [11,12,13]. Quercetin glucosides stored in the body and in the cocoon are reported to have antioxidant, ultraviolet-protective, and antibacterial properties [14,15,16]. Quercetin is found in the leaves of the mulberry tree (*Morus alba*), the sole food source of the mulberry silkworm, as a series of glycosides formed by glycosylation at the 3-*O* position. The three most common quercetin glycosides in mulberry are

quercetin-3-*O*-rutinoside (rutin), quercetin-3-*O*-malonyl-glucoside (Q3MG) and Q3G, which account for 71%–80% of the total flavonol content in the leaves [17].

The color of the cocoon of the domestic silkworm has been diversified through breeding, which indicates that the kinds and amounts of flavonoids that accumulate in the cocoons differ between strains [11,12]. Particularly, cocoons containing high flavonoid concentrations express a yellow-green color and are known as "green cocoons". Because accumulation in the cocoon is the end step of flavonoid metabolism, forward-genetic analysis focused on cocoon flavonoid content can reveal the genes involved in individual steps of flavonoid metabolism. Indeed, several loci associated with flavonoid metabolism have already been identified through this approach: the *Green b* locus, which encodes a uridine 5'-diphospho-glucosyltransferase (UGT) with a rare enzymatic activity glycosylating the 5-*O* position of quercetin [14], and the *New Green Cocoon* (*Gn*) locus, which encodes clustered sugar transporters presumed to import quercetin glucosides from the hemolymph to the silk gland [18]. However, these findings explain only a part of the process from quercetin uptake to the final accumulation of its metabolites in the cocoon. Elucidating the steps of flavonoid metabolism in the silkworm can provide valuable insights into the understanding of herbivore–plant interactions.

Here, we performed a quantitative trait locus (QTL) analysis focused on cocoon flavonoid content in the domestic silkworm and identified a novel locus, *Green d* (*Gd*), which is associated with this trait. Within the locus, we identified a glycoside hydrolase gene, glycoside hydrolase family 1 group G 5 (*GH1G5)*, which mediates quercetin uptake into the midgut cells by deglycosylating mulberry leaf-derived quercetin glycosides. Genetic

dissection of the novel gene revealed the contribution of the gene to improvement of the cocoon in a commercial context through breeding.

## Results

### QTL analysis identified a novel locus associated with flavonoid content in cocoon

To identify genes involved in quercetin metabolism by means of a forward-genetics approach, we prepared a green-cocoon strain (p50; alias: Daizo) and a white-cocoon strain (J01; alias: Nichi01). The two strains exhibited a distinct difference in cocoon color and flavonoid content (Fig 1A and 1B). A gradated range of cocoon colors in their F2 intercross offspring implied that the genetic differences between the two strains associated with flavonoid content were composite (Fig 1C). Therefore, we conducted a QTL analysis, which allows for simultaneous identification of multiple genetic loci involved in a phenotype of interest. The flavonoid content of the cocoons of the F2 population were scored by an absorbance-based method according to a previous report [19]. The QTL analysis was performed by using the phenotypic data of 102 individuals and 1038 genetic markers obtained from double-digest restriction-associated DNA sequencing data [20] (S1 Table). From the composite interval mapping, we identified three significant QTLs for cocoon flavonoid content on chromosomes 15, 20, and 27 (Fig 1C). Previous linkage studies have suggested a locus, named *Green c* (*Gc*) and associated with the yellow-green color of cocoons, at an unknown position on chromosome 15 [21,22]. The QTL on chromosome 27 was presumed to correspond to *Gn* [18]. Since no green cocoon-associated locus has yet been reported on chromosome 20, we named that locus *Green d* (*Gd*). The contribution of the *Gd* locus to cocoon flavonoid content was the second largest of the three QTLs, with a percentage of phenotypic variation explained

by each QTL (PVE) value of 24.57% (Fig 1C, S2 Table). The 95% Bayes credible interval of the *Gd* locus was 7,980,189–10,504,065 bp. The nearest marker to *Gd* was located at 10,265,033 bp, with a logarithm of odds (LOD) score of 19.99. The PVE values of the QTLs on chromosomes 15 and 27 were 7.04% and 56.05%. Significant additive effects were detected between the QTLs on chromosomes 15 and 27 and between those on chromosomes 20 and 27, with corresponding PVEs of 1.93% and 5.47% (S2 Table).

**Fig 1. Quantitive trait loci (QTLs) associated with cocoon flavonoid content.**

(A) Photographs of representative cocoons of the p50 and J01 silkworm strains. Bar = 30 mm. (B) Flavonoid content of p50 and J01 cocoons. Data are means of independent biological replicates ± SD. (C) QTL analysis for cocoon flavonoid content. The horizontal dotted line indicates the threshold of the permutation test (trials = 1000). Phenotype scores on the frequency distribution are relative to a maximum measurement of 10. LOD, logarithm of odds; PVE, percentage of phenotypic variation explained by each QTL. According to a previous study [21], the *Gc* locus is located on chromosome 15, but its detailed genetic or physical position is unknown. Therefore, it cannot be concluded that the QTL peak on chromosome 15 identified here corresponds to the *Gc* locus. (D) Gene models present within the *Gd* locus of the p50T genome assembly. The genes annotated as encoding glycoside hydrolase family 1 proteins are highlighted in black; their IDs are *KWMTBOMO12222*, *KWMTBOMO12223*, *KWMTBOMO12224*, *KWMTBOMO12225*, *KWMTBOMO12227*, *KWMTBOMO12229*, *KWMTBOMO12230*, *KWMTBOMO12233*, and *KWMTBOMO12236* from the upstream side. (E) Expression of the candidate *Gd* genes in the midgut of third-day final instar male larvae. Data are means of independent biological replicates ± SD. TPM, transcripts per million.

## *Gd* locus contains a glycoside hydrolase family 1 gene cluster

Using the genome assembly and gene models of p50T [23], we found a cluster of nine genes encoding glycoside hydrolases (*KWMTBOMO12222–25*, *KWMTBOMO12227*, *KWMTBOMO12229*, *KWMTBOMO12230*, *KWMTBOMO12233*, and *KWMTBOMO12236*) on the *Gd* locus, which were annotated as glycoside hydrolase family 1 (GH1), according to the nomenclature of carbohydrate-active enzymes provided by CAZy [24] (Fig 1D). In mammals, a critical step in quercetin metabolism is deglycosylation of quercetin glucosides by lactase/phlorizin hydrolase (LPH), a member of GH1. LPH is expressed in intestinal epithelial cells and is anchored on the brush border membrane where it hydrolyzes flavonoid glycosides [25,26,27,28]. The resulting free quercetin aglycon is then passively absorbed into the intestinal cells due to its increased lipophilicity. We hypothesized that the *GH1* genes clustered within the *Gd* locus are involved in quercetin metabolism in the midgut lumen of the domestic silkworm, playing a role similar to that of LPH in mammals. In an inferred phylogenetic tree of all 21 GH1 proteins in *B. mori* and their homologous proteins in Holometabola insects (the fruit fly, *Drosophila melanogaster*; the honeybee, *Apis mellifera*; the red flour beetle, *Tribolium castaneum*), the glycoside hydrolases encoded in the *Gd* locus formed a distinct clade (group G), which was supported by a high bootstrap value (100%), suggesting that divergence of the group G-glycoside hydrolases had occurred after insect order divergence (S2 Fig). According to the phylogeny and their genomic positions, we named them as glycoside hydrolase family 1 group G 1–9 (GH1G1–9). The sequence identity among the group G glycoside hydrolase proteins was highest between GH1G2 and GH1G4 at 79.13%, and the sequence similarity was highest between GH1G1 and GH1G9 at 95.56% (S3A and S3B Fig). In addition, signal peptides were predicted in the N-

terminal region of GH1G1, GH1G2, GH1G3, GH1G5, GH1G7 and GH1G9, suggesting they are secretory enzymes (S3A Fig). To identify which of them act in the midgut, we performed RNA-seq-based expression analysis. Three of the candidate genes, *GH1G1*, *GH1G5*, and *GH1G9*, were found to be strongly expressed in the midgut of p50T final instar larvae; the expression levels of these genes were significantly lower in the midgut of strain J01 (Fig 1E). Further examination of the expression profiles of the three genes with high expression in the midgut revealed that *GH1G5* and *GH1G9* were expressed specifically in the midgut, whereas *GH1G1* was expressed most in Malpighian tubules (S4 Fig). Taken together, we identified *GH1G1*, *GH1G5*, and *GH1G9* as candidate *Gd* genes which are involved in quercetin metabolism in the domestic silkworm.

## Functional analysis of the candidate *Gd* genes by CRISPR-Cas9

To investigate the involvement of these candidate genes in quercetin metabolism, we attempted to use a microinjection-mediated CRISPR-Cas9 system to establish p50T lineages in which they had been knocked out. Although we failed to establish knockout lineages for *GH1G1* and *GH1G9* due to the lethality of homozygous frame-shift mutations, we did manage to obtain two knockout lineages of *GH1G5* with different types of frameshift mutations in exon 5. We designated the one with a 5-bp deletion as Δ*Gd1* and the other with a 2-bp deletion as Δ*Gd2* (Fig 2A). The *GH1G5* mutations resulted in a premature stop codon at exon 5 along with shortened amino acid sequence lengths from 492 to 215 in Δ*Gd1*, and from 492 to 216 in Δ*Gd2*. The mutants produced discolored cocoons compared to p50T (Fig 2B). In addition, we observed a reduction of fluorescence under ultraviolet irradiation the midgut, hemolymph, and silk glands of the mutants (Fig 2C–2E). Such fluorescence is characteristic of the accumulation of the two major quercetin metabolites in silkworm, quercetin-5-*O*-glucoside and quercetin-5,4´-di-*O*-glucoside [14,15,29]. Although knockout of *GH1G5* reduced the total flavonoid content in the cocoon by less than half that in the original p50T strain, it was still much larger than the effect predicted for the *Gd* locus in the QTL analysis (Fig 2F and S2 Table). We found similar reductions in the midgut, hemolymph, and middle and posterior silk gland. Because the flavonoid content in the cocoon differed largely between the insects reared with an artificial diet and those reared with fresh mulberry leaves (Fig 1B and Fig 2F), we confirmed the flavonoid content reductions in the mutants in an experiment using fresh mulberry leaves (S5 Fig). Together, these results indicated that knockout of *GH1G5* resulted in malfunction of quercetin uptake into the midgut.

**Fig 2. Reduction of flavonoid content in *GH1G5*-knockout mutants.**

(A) Disrupted target sequences in *GH1G5*. (B)–(E) Cocoons (B), midguts (C), hemolymph (D), and silk glands (E) of the p50T strain and the *GH1G5* mutants. Organs and tissues were taken from sixth-day final instar female larvae. Bars = 10 mm. (F) Total flavonoid content of cocoons, organs, and tissues of the p50T strain and the *KWMTBOMO12227* mutant lineages. Data are means of independent biological replicates ± SD. All insects were reared on a commercial artificial diet. SG, silk gland.

**Sequence and isoform determination for *GH1G5***

The predicted gene model for *GH1G5*, *KWMTBOMO12227*, consists of 11 exons, encoding a total of 492 amino acids. The accuracy of the modeled sequence and its dominance among isoforms were confirmed using Sanger sequencing and the RNA-seq data obtained from final instar larvae of strain p50T. We cloned the putative longest open reading frame in the transcripts of *GH1G5*, and sequenced it by Sanger sequencing (S6A Fig). The determined sequence was absolutely identical to the modeled sequence of *GH1G5*. Previously, our research group reported RNA-seq data for final instar larva tissues of the p50T strain [30]. By mapping the reads of the data derived from the midgut to the genomic sequence of the p50T strain reported by Kawamoto et al. [23], we determined the transcript isoforms of *GH1G5*. The gene model accurately represented the dominant isoform, and another isoform extending 15-bp downstream of the 10th exon was also detected (S6B Fig). The termination codon in the extended region shortened the predicted protein sequence length of this isoform to 456 amino acids. Since the mean coverage of each base of the 15-bp extended region was only about 15% of that of the original 10th exon of *GH1G5* (S6C Fig), we concluded this as a minor isoform.

**GH1G5 mediates quercetin uptake by hydrolysis of quercetin glycosides**

Together, our knockout analysis and sequence characterization suggested that the GH1G5 is secreted into the midgut lumen and mediates the uptake of mulberry-derived quercetin by deglycosylation of quercetin glycosides. However, the knockout mutants still accumulated some flavonoids in the midgut cells (Fig 2F). This might be due to the diversity of quercetin glycosides in mulberry leaves and the substrate specificity of GH1G5. To confirm whether GH1G5 is involved in deglycosylation of quercetin

glycosides, we investigated the hydrolytic activity of midgut tissue of the knockout mutants on the three major quercetin glycosides in mulberry leaf: rutin, Q3MG, and Q3G (Fig 3A).

**Fig 3. Hydrolytic activity of GH1G5 on the three major quercetin glycosides in mulberry leaf.**

(A) Structural formulae of quercetin and the three major quercetin glycosides present in mulberry leaf. (B) Hydrolytic activity of the midgut on the quercetin glycosides. Data are means of independent biological replicates ± SD. The values above the graph are *p*-values calculated by a two-tailed Student's *t*-test. All samples were from fifth-day final instar female larvae reared on a commercial artificial diet. (C) Photograph of representative cocoons of the p50T strain and the mutants reared on semi-synthetic diets containing quercetin or rutin. Bar = 20 mm. (D) Total flavonoid content in cocoons of the p50T strain and the mutants reared on semi-synthetic diets containing quercetin or rutin. Data are means of independent biological replicates ± SD.

The homogenate of the p50T midgut exhibited hydrolytic activity against all three types of quercetin glycoside (Fig 3B). However, the activities were decreased in the knockout mutants, indicating that GH1G5 is involved in the hydrolysis of all three quercetin glycosides. Notably, *GH1G5* knockout completely abolished the hydrolytic activity against rutin, indicating it is the only glycoside hydrolase with hydrolytic activity against this quercetin glycoside. Furthermore, partial reductions were observed in the hydrolytic activity of the other two glycosides, Q3MG and Q3G, was only partial, suggesting that the silkworm has other additional glycosidses with hydrolytic activities against those molecules (Fig 3B). Knocking out *GH1G5* reduced the hydrolytic activity of midgut homogenates against Q3MG and Q3G by 0.6 to 0.8-fold and 0.7 to 0.8-fold, respectively. These results suggested that GH1G5 is an important protein for the hydrolysis of quercetin glycosides in the silkworm lumen.

In 1972, Fujimoto and Hayashiya reported that the domestic silkworm accumulates flavonoids in its cocoon when reared on artificial diets containing isolated quercetin or rutin [31]. To investigate whether the uptake of rutin-derived quercetin is dependent on deglycosylation by GH1G5, we reared insects on semi-synthetic diets supplemented with rutin or quercetin but without mulberry leaf powder and measured the flavonoid content in the cocoons. The p50T strain accumulated flavonoids in its cocoon irrespective of diet (Fig 3C, 3D, and S3 Table). Although the *GH1G5*-knockout mutants accumulated the same amount of flavonoids as did p50T when reared on the quercetin diet, they accumulated only 6% of that accumulated by p50T when reared on the rutin diet (Fig 3C and 3D). These results were consistent with the report by Fujimoto and Hayashiya, and

suggested that the uptake of quercetin from rutin is strongly dependent on deglycosylation by *GH1G5*.

**Evolution of GH1G5**

The phylogeny of the Holometabola GH1 proteins suggested that the divergence of the group G glycoside hydrolases had occurred after insect order divergence (S2 Fig). To estimate when *GH1G5* arose during the evolution of Lepidoptera, we constructed a maximum likelihood-inferred phylogenetic tree of lepidopteran-wide orthologous proteins of the group G glycoside hydrolases in *B. mori* (BmorGH1G1–9) (Fig 4A). The tree classified BmorGH1G1–9 into five clades: one clade including BmorGH1G5, one clade including BmorGH1G1 and BmorGH1G9, one clade including BmorGH1G2, BmorGH1G3 and BmorGH1G4, one clade including BmorGH1G6 and BmorGH1G7, and one clade including BmorGH1G8. The clade including BmorGH1G5 consisted of only proteins from species belonging to Macroheterocera (including Noctuoidea, Lasiocampoidea, and Bombycoidea), suggesting that *GH1G5* had evolved through gene duplication after the divergence of Pyraloidea and Macroheterocera (Fig 4A and 4B). Sequence identities and similarities of the GH1G5-class proteins, excluding the one from *B. mandarina*, to BmorGH1G5 were at a maximum of 57.92% and 89.98% (Fig 4C). OrthoFinder [32], the tool we used for the collection of orthologous proteins of BmorGH1G1–9, detected gene duplication events that had occurred within each orthologous group while simultaneously estimating orthologous relationships. Interestingly, it suggested that the group G glycoside hydrolases had undergone notably frequent duplication events; the estimated number of duplications which occurred within the ortholog group including GH1G5 (also including GH1G2, GH1G4, and GH1G6–9) was 46, which ranked 197th out of 19436 total groups and 1st out of 54 groups including *B. mori* proteins annotated as glycoside hydrolases (InterPro entry: IPR001360 or GO annotation: 0016798) (S4 and S5 Table).

**Fig 4. Phylogenetic tree of lepidopteran-wide orthologous proteins of the group G glycoside hydrolases in *B. mori*.**

(A) The phylogenetic tree was constructed by the maximum likelihood method. Values on the nodes represent the bootstrap scores (trials = 100). Unreliable nodes with bootstrap values under 50 are shown as multi-branching nodes. The tree was rooted using Rat LPH as an outgroup. Red branches represent clades including the group G glycoside hydrolases in *B. mori*. The clade containing BmorGH1G5 is highlighted with a box. (B) Species tree of Lepidoptera drawn/constructed with reference to the report by Kawahara et al. [72]. Red circles indicate species harboring an GH1G5 ortholog. (C) Sequence identities and similarities of the GH1G5-class proteins to BmorGH1G5. Similarity of amino acid residue property is determined according to the groups of strongly similar properties described at Clustal Omega FAQ (https://www.ebi.ac.uk/seqdb/confluence/display/THD/Help+-+Clustal+Omega+FAQ).

**Defective structural mutations of *GH1G5* were broadly disseminated in white-cocoon domestic silkworm strains**

Although accumulation of carotenoids or flavonoids in the cocoon produces a variety of cocoon colors, it is white-cocoon strains that are the most popular in commercial use. Earlier in the present study, we found that defective mutations of *GH1G5* resulted in impaired quercetin absorption, which made cocoon colors closer to white (Fig 2B). Interestingly, the defective mutation of *GH1G5* did not impair the growth of the silkworm, but rather improved silk yield (S7 Fig). The implication here was that this beneficial mutation was selected for in the establishment of white-cocoon domestic silkworm strains.

To examine our hypothesis, we determined conserved *GH1G5* genotypes in a collection of *B. mori* strains, and investigated the frequency and distribution. We first determined the details of the mutation introduced in J01, and then compared them to p50T. Previously, we reported a genome assembly and gene model for J01 [33]. A BLASTp search of our J01 gene model identified predicted gene *BMN13127* as corresponding to *GH1G5* in this strain. Comparing the genomic regions of *GH1G5* in p50T and J01, an insertion of 3997 bp was found in exon 5 of J01 *GH1G5* (Fig 5A). The inserted sequence was identified as the reported non-autonomous transposable element in *B. mori*, *Neet* [34]. The *Neet* sequence and the intronic region between exon 5 and 6 in J01 included the inverted sequence of the intronic region between exon 5 and 6 of *GH1G5* of p50T (Fig S8A), suggesting that it had experienced additional structural changes. A product amplified from the genomic DNA region of exon 5 of J01 including the insertion was approximately 4000-bp longer than that from the p50T genomic DNA (Fig 5B). Probably due to the insertion, the predicted splice site of the exon 5 end shifted 123 bp forward in J01 *GH1G5*,

resulting in 41 amino acids deletion in the predicted protein sequence (Fig 5A and S8B Fig). By cloning and sequencing the J01 transcript using the primer sets that were used to determine the open reading frame sequence of p50T *GH1G5*, the accuracy of the modeled sequence was confirmed (S8C Fig). The deletion included two of nine residues with >99% conservation across 101 homologous proteins from five Macroheterocera species, and all of seven residues which harbored strongly similar physicochemical properties across all of these proteins (S9 Fig). These observations strongly suggested that the deletion was mainly responsible for the observed difference in flavonoid content in the cocoon.

**Fig 5. Conserved structural mutations of *GH1G5* in the domestic silkworm population.**

(A) Schematic illustration comparing exon 5, 6 and the intronic region between them (intron 5) of *GH1G5* from the p50T and J01 strains. (B) PCR identifying the J01-type insertion. The amplified region is indicated by the colored area in (A). The primer set amplifying the genomic region from exons 1 and 2 of *KWMTBOMO14639* (*rp49*) was used as the control (predicted fragment length = 1548 bp). (C) Schematic illustration comparing the *Gd* loci of the p50T and Kosetsu strains. Black boxes indicate the modeled genes. (D) PCR identifying the Kosetsu-type large deletion. The amplified region is indicated by the colored area in (C). (E) RT-PCR of the *GH1G5* full-length open reading frame using cDNA libraries constructed from the midguts of sixth-day final instar larvae. The primer set targeting exons 1 and 2 of *KWMTBOMO14639* (*rp49*) was used as the control (predicted fragment length = 213 bp). (F) Distribution of the haplotypes of *GH1G5* in Japanese and Chinese local silkworm strains. P/P, only the p50T genotype was detected; P/J/K, p50T, J01, and Kosetsu genotypes were detected; J/J, only the J01 genotype was detected; J/K, both J01 and Kosetsu genotypes were detected; K/K, only the Kosetsu genotype was detected; U, unknown, nothing detected. Grayscale colors indicate pairs of loss-of-function haplotypes.

Next, we examined the presence of the J01-type 4-kbp insertion in 67 Japanese and Chinese local strains by PCR using tested primers (S10 Fig and S6 Table). All five green-cocoon strains lacked the insertion. Of the 32 Japanese local white-cocoon strains, the insertion was absent in five and present in seven, whereas the remaining 20 lacked any amplification product. Of the 30 Chinese local white-cocoon strains, the insertion was absent in 15, present in four (p50T-type products were also observed in three strains of them), and 11 again lacked an amplification product. The lack of an amplification product suggested these strains contained another dysfunctional mutation. To determine what it might be, we obtained Illumina whole-genome sequencing data of Kosetsu, one of the strains lacking an observed amplification product, and assembled it. By comparison against the genomic sequence of p50T, a 143.7-kbp deletion including *GH1G5* was found in the Kosetsu *Gd* locus (Fig 5C). When the region including the deletion was amplified by PCR using p50T, J01, and Kosetsu genomic DNA, an observable amplification product was synthesized only from the Kosetsu sample (Fig 5D). In addition, no observable amplification product was synthesized from the Kosetsu sample by using reverse-transcription PCR to amplify the full-length open reading frame of *GH1G5* using cDNA from the midgut of sixth-day final instar larvae of the three strains (Fig 5E). Moreover, the J01-derived product was shorter in size and the band appeared fainter than that from p50T. These results were consistent with the genetic differences of *Gd* among the three strains predicted from the assembled genomic sequences. Using the primer sets for detecting the Kosetsu-type large deletion, we examined the presence of the mutation in all of the prepared 67 silkworm strains. It revealed that 46 of the 67 strains possessed the Kosetsu-type deletion. Genotypes of 26 of the 20 Japanese and 11 Chinese local white-cocoon strains which we failed genotyping were determined as the Kosetsu-type,

whereas the remaining five still lacked any amplification product (S10 Fig). The five strains which we failed genotyping using any of the used primer sets might have undergone further genomic structural changes after acquiring the Kosetsu-type large deletion.

To summarize the genomic PCR analysis, we found three haplotypes of *GH1G5* in the population: p50T-type (P, functional), J01-type (J, harboring a 4-kbp insertion in exon 5), and Kosetsu-type (K, harboring a large deletion eliminating *GH1G5*). The proportions of strains homozygous for the functional haplotype (P), heterozygous for the functional and a dysfunctional (J or K) haplotype, and homozygous for a dysfunctional haplotype were 3%, 13%, and 84% in the Japanese local white-cocoon strains, and 23%, 37%, and 40% in the Chinese local white-cocoon strains (Fig 5F).

## Discussion

### GH1G5 mediates quercetin uptake in *Bombyx mori*

Here, we identified GH1G5 as a glycoside hydrolase that initiates quercetin metabolism in the domestic silkworm (Fig 6). GH1G5, a putative secretory enzyme belonging to GH1, is expressed specifically in the midgut (S4 Fig). Knockout of *GH1G5* reduced total flavonoid content in the midgut, hemolymph, and silk glands, indicating that the gene is involved in quercetin absorption in the midgut, the initial place of entry into the insect (Fig 2C–2F). The hydrolytic activity of the midgut tissue on rutin, Q3MG, and Q3G was reduced in *GH1G5*-knockout mutants (Fig 3B), suggesting that GH1G5 hydrolyses all three major quercetin glycosides present in mulberry leaf. The amount of flavonoids contained in the cocoon of insects reared with a diet supplemented with rutin was greatly reduced in the absence of functional GH1G5 (Fig 3C and 3D), suggesting that deglycosylation of quercetin glycosides by GH1G5 is a critical step in quercetin uptake in the silkworm. The absorbed quercetin may be retainend in midgut cells by decreasing its lipophilicity through 5-*O*-position glucosylation by UGT [14]. The present analyses were limited to studying functional changes in deletion mutants produced by genome editing and spontaneous mutations found by screening diverse domestic silkworm strains. Further biochemical and/or immunological studies will be needed to clarify detailed enzymatic characteristics and biological roles of GH1G5.

**Fig 6. Proposed model of quercetin uptake in the silkworm midgut.**

While revealing a major role for GH1G5 in quercetin uptake in the silkworm, this study also suggests the presence of GH1G5-independent quercetin uptake pathways. The accumulation of flavonoids in the cocoon and tissues was not completely lost in the *GH1G5*-knockout mutants (Fig 2F). In addition, the residual hydrolytic activity of the midgut homogenate on Q3MG and Q3G implies the presence of other glycoside hydrolases mediating quercetin uptake (Fig 3B). Although we were unsuccessful in obtaining knockout lineages for *GH1G1* or *GH1G9*, the proteins they encode may correspond to these other glycoside hydrolases.

**Adaptive significance of quercetin uptake in the mulberry silkworms is supported by the fact that they have retained *GH1G5***

Our results indicate that deglycosylation-dependent flavonoid uptake, which has been well-studied in mammals, is also found in insects. One feature distinguishing the mechanism in the domestic silkworm from that in other animals is the suggested rutin hydrolytic activity of GH1G5 (Fig 3B–3D). Rutin hydrolysis in mammals is dependent on the intestinal microbiota [35, 36]. Rutin glycoside hydrolases have previously been identified in bacteria, fungi, and plants [37,38,39], but to our knowledge, not in animals. Rutin hydrolysis in the honey bee (*Apis mellifera*) also depends on the gut microbiota [40], indicating that endogenous rutin glycoside hydrolases are not common in insects. Rutin is a common plant flavonoid, accounting for 14%–46% of flavonol glycosides in mulberry leaf [17]. The accumulation of flavonoids was severely restricted in the cocoons of *GH1G5*-knockout mutants reared on a rutin-containing diet, suggesting the absence of rutin digestion pathways other than deglycosylation by GH1G5 (Fig 3C and 3D). Therefore, the acquisition of rutin hydrolytic activity should result in a considerable

change in quercetin intake. In addition, the genomic instability of the *Gd* locus emphasizes the significance of quercetin bioavailability brought about by the enzyme. According to the inference of gene duplication events by OrthoFinder [32], the group G glycoside hydrolases are the group that has undergone the most frequent gene duplications among the 54 *B. mori* glycoside hydrolase ortholog groups (S4 and S5 Table). The fact that the large deletion was introduced into the *Gd* locus within a short period of the breeding history of the domestic silkworm also suggests the genomic instability of the region. While the genomic instability of the *Gd* locus had possibly provided genetic material to evolve *GH1G5* by gene duplication events, it can be inferred to have repeatedly exposed *GH1G5* to genomic structural changes which introduce defective mutations into the gene. However, the mulberry silkworms have retained *GH1G5* and its rutin hydrolytic activity until defective mutations were selected in breeding for cocoon improvement. These results support that the suggested positive properties of quercetin glycosides, notably, their antioxidant [15], ultraviolet light-protective [14], and antibacterial functions [16], improve the fitness of the wild silkworm in the natural environment. Although, it should be noted that excessive quercetin uptake is still toxic even for the domestic silkworm [41], meaning that the mulberry silkworms should have a mechanism to maintain appropriate levels of quercetin. The fluorescence in the posterior region of the midgut observed in the present study implies such mechanisms may involve accumulation of quercetin glucosides with glycosylation at the 5-*O* position and reloading of excessive quercetin glucosides from the hemolymph for excretion into the midgut lumen (Fig 2C).

The phylogeny for the lepidopteran orthologous proteins of the group G glycoside hydrolases suggested that *GH1G5* has evolved after the divergence of Pyraloidea and Macroheterocera (Fig 4A and 4B). This indicates that quercetin uptake mediation by GH1G5 is not common in lepidopteran insects. Given the low sequence identities (<58%) between BmorGH1G5 and the GH1G5-class proteins in other Macroheteroceran moths excluding *B. mandarina* (Fig 4C), the rutin hydrolytic activity might be conserved only in the mulberry silkworms. Although we did not investigate rutin hydrolytic activities of the GH1G5-class proteins other than BmorGH1G5, such an investigation could highlight the distinctiveness of the enzymatic property of BmorGH1G5. If so, it would allow for a discussion on the association between the evolution of the rutin hydrolytic activity and the monophagy of the silkworms on mulberry leaves, which contain high levels of rutin [17]. Quercetin uptake is observed in other herbivorous insects [9, 10]; their GH1 proteins may have evolved to adapt to flavonoid compositions in their respective host plants. A species-wide characterization of GH1 proteins in flavonoid glycoside hydrolytic activities would potentially provide insights into dietary habits of herbivorous insects.

### *GH1G5* loss contributed to cocoon improvement

Through breeding, many strains of the domestic silkworm have been established which exhibit a wide variety of cocoon colors depending on their flavonoid and carotenoid content. Nevertheless, white cocoons tend to be preferred commercially in regions such as Japan and China, especially since the 20th century. In a previous study that conducted a worldwide genetic analysis of silkworm strains, 91 of the 121 strains examined were white-cocoon strains [42]. The defective mutation in *GH1G5*, which manifests as reduced cocoon color (Fig 2B), may have contributed to the establishment of these white-cocoon

silkworm strains. Of the white-cocoon strains examined in the present study, only 62% carried homozygous defective haplotypes of *GH1G5* (haplotype J or K; Fig 5F, S10 Fig, and S6 Table). In 24% of the strains, a mixture of defective and functional haplotypes (haplotype P) was present. These are not surprising results, considering that not all white-cocoon strains harbor loss-of-function mutations of the sugar transporters encoded in the *Gn* locus, which is more effective than *Gd* with respect to cocoon discoloration [18]. Even if visually determined as white, the actual color tone and therefore the flavonoid content of the cocoon varies from strain to strain [43]. In addition to *Gd*, *Gb*, and *Gn*, within each of which a gene involved in the quercetin metabolism has been identified, several loci associated with cocoon greenness have been reported, including *Gc* [21,22], *Green cocoon* (*Grc*), *Green egg shell* (*Gre*), and *Yellow fluorescent* (*Yf*) [21]. "White cocoon" is presumed to be expressed by additive cocoon color discoloration by a combination of loss-of-function haplotypes of these loci.

Although loss of *GH1G5* is not necessarily essential for cocoon whiteness, given the high conservation rate of loss-of-function haplotypes and the fact that knockout of the gene reduces the cocoon total flavonoid content by less than half (Fig 2F, Fig 5F), the gene still makes an important contribution to cocoon discoloration in the domestic silkworm. Interestingly, the knockout of *GH1G5* increased the cocoon weight (S7 Fig). Because endoreduplication is an important step in silkworm silk gland development [44], interference with the replication step by quercetin interaction with DNA may explain the relationship between *GH1G5* and silk yield [45]. Although it will be difficult to confirm this hypothesis because cocoon weight is a complex quantitative trait dependent on

silkworm physiology and behavior, elucidation of the relationship between quercetin and silk yield may provide clues to further improve silkworm protein productivity.

## Materials and Methods

### Insect materials

All domestic silkworm strains used in the present study were maintained at the National Agriculture and Food Research Organization (NARO, Japan). p50T, the strain used for the functional analysis of *GH1G5*, is almost genetically identical to p50, because it is a descendant of p50 which had undergone repeated passage using a single pair to improve homozygosity for referential use. The silkworms were reared on a commercial artificial diet (SilkMate PM; Nosan, Kanagawa, Japan) or fresh mulberry leaves under a controlled environment (12-h light/dark photoperiod, 25°C). Insects reared on fresh mulberry leaves were used for measurement of the flavonoid contents of the cocoons of p50 and J01 (Fig 1A and 1B), the scoring for QTL analysis (Fig 1C), and measurement of the flavonoid contents of the cocoons of p50T and *GH1G5*-knockout mutants (S5 Fig). All individuals were female except those from which genomic DNA and RNA-seq data were derived.

### Flavonoid content measurement

After shredding the cocoons into 2–3-mm squares, flavonoids were extracted from the pieces with MeOH–$H_2O$ (7:3, V/V) at 60ºC for 2 h. The extraction was repeated twice for thorough extraction. The flavonoid content in the solution was derived from the absorption at 365 nm, which is highly correlated with flavonoid content ($r$ = 0.95) [19]. To use flavonoid content as an input for QTL analysis, the raw absorbance values were used as relative phenotype scores. The tissues and organs for measuring flavonoid content were sampled from sixth-day final instar larvae reared on a commercial artificial diet, rinsed well with PBS buffer (pH 7.4) (Takara Bio, Shiga, Japan), and stored at −80ºC. To inhibit melanization in hemolymph, immediately after collection 0.5 M sodium

dimethyldithiocarbamate (Fujifilm, Tokyo, Japan) was added to reach 0.5% by volume. Quantification of flavonoid content in the samples was performed according to a previous report [15] with some modifications. In brief, 30 μL of sample was injected into an LC-10ADVP HPLC system equipped with an SPD-10AV photodiode array detector (both Shimadzu Co., Kyoto, Japan) and separated with a Sunfire C18 column (150 × 3.0 mm i.d.; Waters, Massachusetts, USA) at a flow rate of 1.0 mL/min. Spectrum analysis and flavonoid peak assignment were performed with the CLASS-VP HPLC software (Shimadzu Co.). Flavonoids were quantified using quercetin 5, 4´-di-*O*-glucoside as the reference compound.

**QTL analysis**

The intercross F2 population of p50T and J01 used in the present study were the same samples as those sequenced by double-digest restriction-associated DNA sequencing and used to generate linkage maps in our previous study [33]. The sequencing data of 102 female F2 individuals are available in the Sequence Read Archive under the BioProject accession number PRJDB13956. The raw values of absorbance indicating the relative cocoon flavonoid content for each individual are summarized in S1 Table. The method for genotyping, marker selection, and construction of linkage maps is described in detail in our previous report [33]. Briefly, OneMap v2.8.2 [46] was used to construct linkage maps with an LOD score of 3, with the genotype of the F2 population output from the script "ref_map.pl" in STACKS v1.48 [47] as the input. The p50T genome assembly was used as the reference for read mapping [23]. The linkage map consisted of 1038 markers covering a total genetic length of 945.36 cM, with an average marker density of 0.936 cM. QTL analysis was performed using the R/qtl v1.46.2 package [48], with the relative

flavonoid content scores in the cocoons and the genetic position of markers linked to the physical position on the p50T genomic sequence as inputs. Using the "calc.genoprob" function in R/qtl, the probabilities of the true underlying genotypes were calculated with a step size of 1 cM and an assumed genotyping error rate of 0.05. QTL detection was performed by composite interval mapping using the function "cim" with the following parameters: method = "hk", n.marcovar = 3, window = 10. The LOD significance threshold for detecting QTL was calculated by a permutation test of 1000 trials. Approximate Bayesian 95% credible intervals were calculated using the function "bayesint". The positions of the nearest markers outside of the boundaries of a confidence interval were defined as the ends of the interval. PVEs and additive effects of the three significant QTLs were estimated using the function "fitqtl" with the parameter: formula = y ~ Q1 + Q2 + Q3 + Q1 * Q2 * Q3.

**Phylogenetic analysis**

GH1 proteins in *B. mori* were identified using InterPro annotation (InterPro entry: IPR001360, Glycoside hydrolase family 1) [49]. An InterPro annotation result for the p50T predicted protein sequences is available in KAIKObase [50] (https://kaikobase.dna.affrc.go.jp/index.html). The related proteins in Holometabola insects were collected by BLASTp search using the 21 *B. mori* GH1 proteins as the query. Genes with e-values less than 1E-50 were used for the analysis. InsectBase 2.0 (http://v2.insect-genome.com/) [51] was used for picking related proteins in *Drosophila melanogaster*, *Tribolium castaneum*, and *Apis mellifera*. Arabidopsis thioglucoside glucohydrolase 1 (TGG1) was used as an outgroup. The query and these sequences were aligned using Clustal Omega v1.2.4 [52]. Tree construction was performed by the

neighbor-joining method using MEGA 11 [53] with the number of bootstrap trials set to 100. The phylogenetic tree of lepidopteran orthologs of the *Gd* glycoside hydrolases was constructed by the maximum likelihood method using RAxML-NG v1.1 [54] with the following parameters: --bs-trees 100, --model LG+I+G4 (S11 Fig A). The best-fit phylogenetic analysis model was suggested using ModelTest-NG v0.1.7 [55]. Gene models of 12 lepidopteran insects available in InsectBase 2.0 were used (*Papilio Xuthus*, *Megathymus ursus*, *Lycaena phlaeas*, *Limenitis camilla*, *Chilo suppressalis*, *Biston betularia*, *Pheosia gnoma*, *Dendrolimus punctatus*, *Bombyx mori*, *Bombyx mandarina*, *Antheraea yamamai*, *Deilephila porcellus*). Each of the models was predicted based on high-quality genome sequences using long-read sequencing, and their BUSCO [56] scores were all above 94%. The tree included a total of 90 lepidopteran protein sequences, which were inferred belong to the same ortholog groups as the nine group-G glycoside hydrolases by OrthoFinder [32] v2.5.2, and Rat LPH (NCBI Refseq accession: NP_446293) as an outgroup.

## Gene editing

CHOPCHOP v3 was used to identify target sites [57]. Each 20-nucleotide guide sequence was unique on the p50T genomic sequence [23]. The construction of DNA templates for the synthesis of single-guide RNA (sgRNA) was performed according to a previous report [58]. Briefly, the DNA template, which included the T7 promoter, sgRNA target with PAM motif, and tracrRNA-complemental sequence, was synthesized by template-free PCR. A MEGAshortscript T7 Transcription Kit (Thermo Fisher Scientific, Waltham, Massachusetts, U.S.A.) was used for *in vitro* sgRNA transcription and template DNA digestion. A Guide-it IVT RNA Clean-Up Kit (Takara Bio) was used to clean up the

sgRNA solution. Alt-RS.p. HiFi Cas9 Nuclease V3 solution (Integrated DNA Technologies, Coralville, Iowa, U.S.A.) and the sgRNA were mixed with distilled water to final concentrations of 500 ng/μL and 50 ng/μL. The Cas9 nuclease and sgRNA mixture was incubated for 1 h at room temperature to allow the ribonucleoprotein to form. The ribonucleoprotein solution was microinjected into non-diapause eggs of the p50T strain within 8 h after oviposition, in accordance with a previous report [59]. Non-diapause treatment was performed by incubating eggs under 17°C short-day conditions (12-h light/dark photoperiod) until hatching. Adult moths of G0 individuals (injected generation) were crossed with wild-type moths. Individuals harboring identical heterozygous mutant haplotypes were identified by high-resolution melting (HRM) analysis using genomic DNA extracted from G1 generation molt shells harvested from cocoons. PCR for HRM analysis was performed using a KAPA HRM Fast PCR Kit (Roche, Basel, Switzerland) on a LightCycler 96 System (Roche) according to the manufacturer's instruction. Genomic DNA extraction from molt shells was performed by homogenizing the shells in a typical SDS-based lysis buffer followed by isopropanol precipitation. G2 individuals with a homozygous mutant haplotype were identified and crossed by the same method to establish the knockout lines. To confirm the presence of a mutation PCR products which included the target site were sequenced using a BigDye Terminator v3.1 Cycle Sequencing Kit and an Applied Biosystems 3130xl Genetic Analyzer (Thermo Fisher Scientific). The primer sequences used are listed in S7 Table.

**Photographing of dissected samples**

Sixth-day fifth instar larvae reared on the artificial diet were used. Photographs were collected with a DP74 camera (Olympus, Tokyo, Japan) attached to an SZX16

microscope (Olympus). To inhibit melanization of collected hemolymph, 0.5 M sodium dimethyldithiocarbamate (Fujifilm) was added immediately after collection to reach 0.5% by volume. To remove fluorescent hemolymph before imaging, silk glands and whole-body samples from which silk glands had been removed were rinsed well with PBS buffer (pH 7.4) (Takara Bio). A fluorescence light source U-HGLGPS (Olympus) and ultraviolet light filter set (excitation: 330–385 nm, emission: 420 nm longpass) (Olympus) were used for imaging under ultraviolet light irradiation. Because of the loss of resolution and optical noise when the field of view was made large enough to fit whole bodies, these samples were photographed in segments and then the pictures were combined. In the photographs shown in Fig 2, no post-editing of any kind was performed other than cropping.

**Determination of *GH1G5* open reading frame sequence**

Total RNA was extracted from the midgut of sixth-day final instar larvae using ISOGEN (Nippon Gene, Tokyo, Japan) according to the manufacturer's instructions. Contaminating DNA in the RNA solution was digested using RNase-Free DNase Set (Qiagen, Hilden, Germany). The isolated RNA was purified using an RNeasy Kit (Qiagen). Equal volumes of RNA solutions from five individuals adjusted to the same concentration were mixed to obtain a single bulk sample. Reverse transcription was performed using ReverTra Ace qPCR RT Master Mix (Toyobo, Osaka, Japan) according to the manufacturer's instruction, and the final concentration of the RNA was adjusted to 10 ng/μL. KOD One PCR Master Mix -Blue- (Toyobo) was used for PCR according to the manufacturer's instructions. The conditions of preincubation, denaturation, annealing, and extension were set to 30 s at 95°C, 10 s at 98°C, 10 s at 55°C, and 5 s at 68°C, for 42

cycles. Amplification products were purified using QIAquick PCR Purification Kit (Qiagen) and cloned into a vector using Zero Blunt TOPO PCR Cloning Kit (Thermo Fisher Scientific). Sequencing was performed as described in the subsection above, gene editing. The primer sequences are listed in S7 Table.

**Isoform identification and expressional analysis**

RNA-seq data from the midgut and other organs of third-day fifth instar larvae of p50T and J01 were previously published by our research group [30,33]. The sequencing reads were trimmed by fastp v0.20.0 [60] with the following parameters: -q 20 -n 5 -l 100. Read mapping to the p50T genome assembly and transcript isoform identification were conducted using HISAT2 v2.1.0 [61] and SrtingTie2 v2.2.0 [62] with default parameters. Salmon v1.5.2 [63] was used to calculate transcripts per kilobase million scores. The modeled transcript sequence of p50T was used as the reference [23].

**Assay for hydrolytic activity of the midgut**

Each midgut of four independent fifth-day final instar larvae reared on the commercial artificial diet was collected, rinsed well with PBS buffer (pH 7.4) (Takara Bio), and immediately stored at −80ºC. Three times the weight of 10 mM phosphate/1 mM PMSF buffer (pH 7.5) as the sample was added prior to homogenization, which was throughly performed on ice, using a metal homozenizer Physcotron with a microshaft NS-4 (Microtec, Chiba, Japan). Three times the weight of a 20 mM phosphoric acid (pH 5.5) solution as the homogenate was add prior to hydrolysis, which was performed independently with each of the three substrates, rutin, Q3MG and Q3G, at a concentration of 2 mM for 4 h at 37ºC. Precedently, the enzyme-independent degradation of the three

quercetin glycosides and quercetin in the reaction was examined using the homogenate deactivated by methanol (three volumes (V/V) of methanol were added to the reaction liquid). We measured the change in amounts of quercetin from the substrates at the 0 h and 4 h after the reaction. It confirmed that they were stable in the condition. Control incubations in which the substrates were omitted were also run for calibration taking into account a flavonoid content in the tissue. The reaction was stopped by adding three volumes (V/V) of methanol. After centrifugation at 20,000*g* for 10 min, the quercetin content of the supernatant was quantified with the HPLC system as described in the section "Flavonoid content measurement." Quercetin, rutin, and Q3G were obtained from Extrasynthese (Lyon, France). Q3MG was obtained from Merck (Darmstadt, Germany). Hydrolytic activity for the quercetin glycosides is expressed as nanomoles of quercetin produced per min per mg protein. The protein concentration in the enzyme preparations was measured with a commercial assay kit (Coomassie Plus, Thermo Fisher Scientific).

**Dietary administration of quercetin or rutin**

Silkworm larvae were reared on the commercial diet from hatching to the third ecdysis, then the fourth instar larvae were fed with a diet containing 25% mulberry leaf powder [64]. The newly molted fifth instar larvae were reared on semi-synthetic diets supplemented with quercetin or rutin, which did not contain mulberry leaf powder (S3 Table). Diets were supplemented with flavonoids in approximately equimolar (0.1 mmol/100-g dry diet) amounts, and chlorogenic acid was added as a feeding stimulant. Soybean meal (Soya flour FT; Nisshin OilliO Group,. Ltd., Tokyo, Japan) was washed twice with five volumes of 90% (V/V) ethanol to remove deterrent substances and allowed to dry naturally before use in the diets.

## Genomic comparison of the *Gd* locus

Dot-plot analysis was performed using FlexiDot v1.06 [65] with the following parameters: -p 1 -f 1 -A 2 -E 100. The predicted protein sequence of J01 *GH1G5* (*BMN13127*) presented here (S8B Fig) was modified from the original model, notably, the incorrect prediction of exon 1 was modified according to *KWMTBOMO12227*, and the J01 transcript sequence was obtained by using the original RNA-seq data [33]. DNA extraction from the whole body of a male larva of the domestic silkworm strain Kosetsu was performed by a standard phenol-chloroform-based method. Whole genomic sequencing was performed by an outsourcing service (Macrogen Japan Corp., Kyoto, Japan) using an Illumina NovaSeq 6000 Sequencing System (Illumina Inc., San Diego, USA). The sequencing generated 50.4 Gb of paired-end reads with a length of 151 bp. Genome assembly of the Kosetsu strain was performed using MaSuRCA v4.0.9 [66] with pseudomolecule sequences of chromosomes 15, 20, and 27 of p50T [23], which, contain the QTLs, as the assembly reference. The parameter defining 20 times genomic size "JF_SIZE" was set to 9,000,000,000. The input genomic sequencing data of the Kosetsu strain was trimmed by fastp v0.20.0 [60] before assembly with the following parameters: -q 20 -n 5 -l 100.

## PCR-based genotyping

The genomic DNA of each strain was extracted from 10 pairs of silk glands of final instar larvae by a standard phenol-chloroform-based method. The final concentration of genomic DNA was adjusted to 0.5 ng/μL. KOD One PCR Master Mix -Blue- (Toyobo) was used for PCR according to the manufacturer's instructions. The conditions of

preincubation, denaturation, annealing, and extension were set to 30 s at 95°C, 10 s at 98°C, 10 s at 60°C, and 45 s at 68°C, for 32 cycles. The primer sequences are listed in S7 Table.

**Reverse-transcription PCR**

Reverse transcription was performed as described in the subsection above, determination of *GH1G5* open reading frame sequence. RT-PCR (Fig 5E) was performed using KAPA HiFi HotStart ReadyMix (Roche) under conditions that resulted in 1% of the concentration of the original cDNA solution. The conditions of preincubation, denaturation, annealing, and extension were set to 180 s at 95°C, 5 s at 98°C, 10 s at 60°C, and 60 s at 72°C, for 28 cycles. The primer sequences are listed in S7 Table.

**Detecting gene duplication events**

Detecting gene duplication events in the lepidopteran glycoside hydrolases was performed using OrthoFinder v2.5.2 [32] together with the ortholog-group inference for the phylogenetic analysis. Proteins annotated as "Glycoside hydrolase superfamily (IPR017853)" or with the functional annotation "hydrolase activity, acting on glycosyl bonds (GO:0016798)," were considered to be glycoside hydrolases.

**Other informatic tools**

Handling sequences: Seqkit v2.2.0 [67]; statistical calculations: R v4.0.3 (R Foundation for Statistical Computing, Vienna, Austria); illustrating graphs: ggplot2 v3.4.1 [68] and ggpubr v0.6.0 (https://CRAN.R-project.org/package=ggpubr); alignment visualization: SnapGene Viewer (https://www.snapgene.com/); prediction of signal peptide domains:

SignalP v6.0 [69]; illustrating structural formulae: Ketcher v2.8.0 (https://github.com/epam/ketcher); genomic browsing: Integrative Genomics Viewer v2.12.3 [70]; phylogenetic tree editing: Interactive Tree Of Life v5 [71].

## Supplementary information

Data underlying the reported results is summarized in S1 Data. Legends of supplementary figures (S1–S10 Fig) and tables (S1–S7 Table) are available at the end of the manuscript.

## Data availability

The raw genomic sequencing data of the Kosetsu strain are available in the Sequence Read Archive under the BioProject accession number PRJDB15366.

## Acknowledgements

We thank Ms. Mayuko Sakatoh for her technical assistance; Sae Furuhashi, Toshihiko Misawa, Kaoru Nakamura, Keita Hiruma, Koji Hashimoto, and Eiji Okada for their support in the silkworm rearing; Hiroki Sakai for giving instruction on the CRISPR-Cas9 system construction; Nobuto Yamada for giving instruction on the microinjection; anonymous reviewers for constructive suggestions; Dr. Goldsmith for improving English expressions. The computation was partly performed using the supercomputer of the Agriculture, Forestry and Fisheries Research Information Technology Center of the Japanese Ministry of Agriculture, Forestry and Fisheries.

## Author contributions

RW: Conceptualization, Data Curation, Formal Analysis, Investigation, Visualization, Writing – original draft preparation, Writing – review & editing; CH: Conceptualization, Investigation, Writing – review & editing; ST: Resources, Writing – review & editing, Supervision; TI: Conceptualization, Funding Acquisition, Investigation, Project Administration, Resources; SK: Investigation; AJ: Investigation, Writing – review & editing; TT: Resources, Writing – review & editing; KYo: Resources; KYa: Supervision; HS: Funding Acquisition, Supervision

**Funding**

This work was supported by MAFF Commissioned project study on "Research project for sericultural bio-industry" Grant Number JP22680575 to R.W., T.I. and S.T. and JSPS KAKENHI Grant Numbers 21H03831 to K.Y. and H.S.. The funders had no role in study design, data collection and analysis, decision to publish, or preparation of the manuscript.

**Competing interests**

The authors declare no competing interests.

**References**

1. Cao J, Chen W, Zhang Y, Zhang Y, Zhao X. Content of Selected Flavonoids in 100 Edible Vegetables and Fruits. FSTR. 2010;16: 395–402. doi:10.3136/fstr.16.395

2. Bernays EA, Howard JJ, Champagne D, Estesen BJ. Rutin: a phagostimulant for the polyphagous acridid Schistocerca americana. Entomologia Experimentalis et Applicata. 1991;60: 19–28. doi:10.1111/j.1570-7458.1991.tb01518.x

3. Hamamura Y, Hayashiya K, Naito K-I, Matsuura K, Nishida J. Food Selection By Silkworm Larvae. Nature. 1962;194: 754–755. doi:10.1038/194754a0

4. Haribal M, Renwick JAA. Oviposition stimulants for the monarch butterfly: Flavonol glycosides from Asclepias curassavica. Phytochemistry. 1996;41: 139–144. doi:10.1016/0031-9422(95)00511-0

5. Matsuda K. Feeding Stimulation of Flavonoids for Various Leaf Beetles (Coleoptera : Chrysomelidae). Applied Entomology and Zoology. 1978;13: 228–230. doi:10.1303/aez.13.228

6. Tebayashi S, Matsuyama S, Suzuki T, Kuwahara Y, Nemoto T, Fujii K. Quercimeritrin: The Third Oviposition Stimulant of the Azuki Bean Weevil from the Host Azuki Bean. J Pestic Sci. 1995;20: 299–305. doi:10.1584/jpestics.20.299

7. Burghardt F, Knüttel H, Becker M, Fiedler K. Flavonoid wing pigments increase attractiveness of female common blue (Polyommatus icarus) butterflies to mate-searching males. Naturwissenschaften. 2000;87: 304–307. doi:10.1007/s001140050726

8. Burghardt F, Proksch P, Fiedler K. Flavonoid sequestration by the common blue butterfly Polyommatus icarus: quantitative intraspecific variation in relation to larval hostplant, sex and body size. Biochemical Systematics and Ecology. 2001;29: 875–889. doi:10.1016/S0305-1978(01)00036-9

9. Schittko U, Burghardt F, Fiedler K, Wray V, Proksch P. Sequestration and distribution of flavonoids in the common blue butterfly Polyommatus icarus reared on Trifolium repens. Phytochemistry. 1999;51: 609–614. doi:10.1016/S0031-9422(98)00746-8

10. Hopkins TL, Ahmad SA. Flavonoid wing pigments in grasshoppers. Experientia. 1991;47: 1089–1091. doi:10.1007/BF01923349

11. Fujimoto N, Hayashiya K. Studies on the pigments of cocoon. (VIII) On the classification of the silkworm races and its geographical distribution from the viewpoint of the rate of cocoon pigment contents. Journal of Insect Biotechnology and Sericology. 1961;30: 83–88. doi:10.11416/kontyushigen1930.30.83

12. Hirayama C, Kosegawa E, Tamura Y, Nakamura M. Analysis of flavonoids in the cocoon layer of the silkworm regional races by LC-MS. SANSHI-KONCHU BIOTEC. 2009;78: 57–63. doi:10.11416/konchubiotec.78.1_57

13. Sakudoh T, Sezutsu H, Nakashima T, Kobayashi I, Fujimoto H, Uchino K, et al. Carotenoid silk coloration is controlled by a carotenoid-binding protein, a product of the Yellow blood gene. Proceedings of the National Academy of Sciences. 2007;104: 8941–8946. doi:10.1073/pnas.0702860104

14. Daimon T, Hirayama C, Kanai M, Ruike Y, Meng Y, Kosegawa E, et al. The silkworm Green b locus encodes a quercetin 5-O-glucosyltransferase that produces green cocoons with UV-shielding properties. Proceedings of the National Academy of Sciences. 2010;107: 11471–11476. doi:10.1073/pnas.1000479107

15. Hirayama C, Ono H, Tamura Y, Konno K, Nakamura M. Regioselective formation of quercetin 5-O-glucoside from orally administered quercetin in the silkworm, Bombyx mori. Phytochemistry. 2008;69: 1141–1149. doi:10.1016/j.phytochem.2007.11.009

16. Kurioka A, Ishizaka H, Yamazaki M, Endo M. Antibacterial activity of cocoon shells. J Silk Sci Tech Jpn. 1999;8: 57–60. doi:10.11417/silk.8.57

17. Sugiyama M, Katsube T, Koyama A, Itamura H. Varietal Differences in the Flavonol Content of Mulberry (Morus spp.) Leaves and Genetic Analysis of Quercetin 3-(6-Malonylglucoside) for Component Breeding. J Agric Food Chem. 2013;61: 9140–9147. doi:10.1021/jf403136w

18. Lu Y, Luo J, An E, Lu B, Wei Y, Chen X, et al. Deciphering the Genetic Basis of Silkworm Cocoon Colors Provides New Insights into Biological Coloration and Phenotypic Diversification. Molecular Biology and Evolution. 2023;40: msad017. doi:10.1093/molbev/msad017

19. Hirayama C, Okada E. Simple Spectrophotometric Analysis of Flavonoids from the Cocoon Shell of the Silkworm, Bombyx mori. J Silk Sci Tech Jpn. 2014;22: 65–70. doi:10.11417/silk.22.65

20. Peterson BK, Weber JN, Kay EH, Fisher HS, Hoekstra HE. Double Digest RADseq: An Inexpensive Method for De Novo SNP Discovery and Genotyping in Model and Non-Model Species. PLOS ONE. 2012;7: e37135. doi:10.1371/journal.pone.0037135

21. Fujii H, Banno Y, Doira H, Kihara H, Kawaguchi Y. Genetical Stocks and Mutations of Bombyx mori: Important Genetic Resoueces. 2nd ed. 1998.

22. Fujimoto N. On the physiological functions of the genes concerning the formation and the translocation of the pigments of green cocoon in silkworm larvae. The Journal of Sericultural Science of Japan. 1963;32: 338–342. doi:10.11416/kontyushigen1930.32.338

23. Kawamoto M, Jouraku A, Toyoda A, Yokoi K, Minakuchi Y, Katsuma S, et al. High-quality genome assembly of the silkworm, Bombyx mori. Insect Biochemistry and Molecular Biology. 2019;107: 53–62. doi:10.1016/j.ibmb.2019.02.002

24. Cantarel BL, Coutinho PM, Rancurel C, Bernard T, Lombard V, Henrissat B. The Carbohydrate-Active EnZymes database (CAZy): an expert resource for Glycogenomics. Nucleic Acids Research. 2009;37: D233–D238. doi:10.1093/nar/gkn663

25. Day AJ, Cañada FJ, Díaz JC, Kroon PA, Mclauchlan R, Faulds CB, et al. Dietary flavonoid and isoflavone glycosides are hydrolysed by the lactase site of lactase phlorizin hydrolase. FEBS Letters. 2000;468: 166–170. doi:10.1016/S0014-5793(00)01211-4

26. Mantei N, Villa M, Enzler T, Wacker H, Boll W, James P, et al. Complete primary structure of human and rabbit lactase-phlorizin hydrolase: implications for biosynthesis, membrane anchoring and evolution of the enzyme. The EMBO Journal. 1988;7: 2705–2713. doi:10.1002/j.1460-2075.1988.tb03124.x

27. Németh K, Plumb GW, Berrin J-G, Juge N, Jacob R, Naim HY, et al. Deglycosylation by small intestinal epithelial cell β-glucosidases is a critical step in the absorption and metabolism of dietary flavonoid glycosides in humans. Eur J Nutr. 2003;42: 29–42. doi:10.1007/s00394-003-0397-3

28. Wacker H, Keller P, Falchetto R, Legler G, Semenza G. Location of the two catalytic sites in intestinal lactase-phlorizin hydrolase. Comparison with sucrase-isomaltase and with other glycosidases, the membrane anchor of lactase-phlorizin hydrolase. Journal of Biological Chemistry. 1992;267: 18744–18752. doi:10.1016/S0021-9258(19)37024-3

29. Tamura Y, Nakajima K, Nagayasu K, Takabayashi C. Flavonoid 5-glucosides from the cocoon shell of the silkworm, Bombyx mori. Phytochemistry. 2002;59: 275–278. doi:10.1016/S0031-9422(01)00477-0

30. Yokoi K, Tsubota T, Jouraku A, Sezutsu H, Bono H. Reference Transcriptome Data in Silkworm Bombyx mori. Insects. 2021;12: 519. doi:10.3390/insects12060519

31. Fujimoto N, Hayashiya K. Studies on the pigments of cocoon. (IX) The precursor of the pigments of green cocoon in the silkworm, Bombyx mori. Journal of Insect Biotechnology and Sericology. 1972;41: 383–386. doi:10.11416/kontyushigen1930.41.383

32. Emms DM, Kelly S. OrthoFinder: phylogenetic orthology inference for comparative genomics. Genome Biology. 2019;20: 238. doi:10.1186/s13059-019-1832-y

33. Waizumi R, Tsubota T, Jouraku A, Kuwazaki S, Yokoi K, Iizuka T, et al. Highly accurate genome assembly of an improved high-yielding silkworm strain, Nichi01. G3 Genes|Genomes|Genetics. 2023;13. doi:10.1093/g3journal/jkad044

34. Abe H, Fujii T, Shimada T, Mita K. Novel non-autonomous transposable elements on W chromosome of the silkworm, Bombyx mori. J Genet. 2010;89: 375–387. doi:10.1007/s12041-010-0049-3

35. Ioku K, Pongpiriyadacha Y, Konishi Y, Takei Y, Nakatani N, Terao J. beta-Glucosidase activity in the rat small intestine toward quercetin monoglucosides. Biosci Biotechnol Biochem. 1998;62: 1428–1431. doi:10.1271/bbb.62.1428

36. Riva A, Kolimár D, Spittler A, Wisgrill L, Herbold CW, Abrankó L, et al. Conversion of Rutin, a Prevalent Dietary Flavonol, by the Human Gut Microbiota. Frontiers in Microbiology. 2020;11. Available: https://www.frontiersin.org/articles/10.3389/fmicb.2020.585428

37. Beekwilder J, Marcozzi D, Vecchi S, de Vos R, Janssen P, Francke C, et al. Characterization of Rhamnosidases from Lactobacillus plantarum and Lactobacillus acidophilus. Applied and Environmental Microbiology. 2009;75: 3447–3454. doi:10.1128/AEM.02675-08

38. Pachl P, Kapešová J, Brynda J, Biedermannová L, Pelantová H, Bojarová P, et al. Rutinosidase from Aspergillus niger: crystal structure and insight into the enzymatic activity. The FEBS Journal. 2020;287: 3315–3327. doi:10.1111/febs.15208

39. Sun Y, Cui X, Wang Z. Characterization of a rutin-hydrolyzing enzyme with β-glucosidase activity from tartary buckwheat (Fagopyrum tartaricum) seeds. Food Sci Technol. 2022;42: e42822. doi:10.1590/fst.42822

40. Kešnerová L, Mars RAT, Ellegaard KM, Troilo M, Sauer U, Engel P. Disentangling metabolic functions of bacteria in the honey bee gut. PLOS Biology. 2017;15: e2003467. doi:10.1371/journal.pbio.2003467

41. Shi G, Kang Z, Ren F, Zhou Y, Guo P. Effects of Quercetin on the Growth and Expression of Immune-Pathway-Related Genes in Silkworm (Lepidoptera: Bombycidae). Bendena B, editor. Journal of Insect Science. 2020;20: 23. doi:10.1093/jisesa/ieaa124

42. Xiang H, Liu X, Li M, Zhu Y, Wang L, Cui Y, et al. The evolutionary road from wild moth to domestic silkworm. Nat Ecol Evol. 2018;2: 1268–1279. doi:10.1038/s41559-018-0593-4

43. Hirayama C, Kosegawa E. Identification of fluorescence in white cocoon shells of the silkworm based on three-dimensional fluorescence spectroscopy. SANSHI-KONCHU BIOTEC. 2021;90: 189–194. doi:10.11416/konchubiotec.90.3_189

44. Perdrix-Gillot S. DNA synthesis and endomitoses in the giant nuclci of the silkgland of Bombyx mori. Biochimie. 1979;61: 171–204. doi:10.1016/S0300-9084(79)80066-8

45. Srivastava S, Somasagara RR, Hegde M, Nishana M, Tadi SK, Srivastava M, et al. Quercetin, a Natural Flavonoid Interacts with DNA, Arrests Cell Cycle and Causes Tumor Regression by Activating Mitochondrial Pathway of Apoptosis. Sci Rep. 2016;6: 24049. doi:10.1038/srep24049

46. Margarido GRA, Souza AP, Garcia A a. F. OneMap: software for genetic mapping in outcrossing species. Hereditas. 2007;144: 78–79. doi:10.1111/j.2007.0018-0661.02000.x

47. Catchen JM, Amores A, Hohenlohe P, Cresko W, Postlethwait JH. Stacks: Building and Genotyping Loci De Novo From Short-Read Sequences. G3 Genes|Genomes|Genetics. 2011;1: 171–182. doi:10.1534/g3.111.000240

48. Broman KW, Wu H, Sen Ś, Churchill GA. R/qtl: QTL mapping in experimental crosses. Bioinformatics. 2003;19: 889–890. doi:10.1093/bioinformatics/btg112

49. Zdobnov EM, Apweiler R. InterProScan – an integration platform for the signature-recognition methods in InterPro. Bioinformatics. 2001;17: 847–848. doi:10.1093/bioinformatics/17.9.847

50. Yang C, Yokoi K, Yamamoto K, Jouraku A. An update of KAIKObase, the silkworm genome database. Database. 2021;2021: baaa099. doi:10.1093/database/baaa099

51. Mei Y, Jing D, Tang S, Chen X, Chen H, Duanmu H, et al. InsectBase 2.0: a comprehensive gene resource for insects. Nucleic Acids Research. 2022;50: D1040–D1045. doi:10.1093/nar/gkab1090

52. Sievers F, Wilm A, Dineen D, Gibson TJ, Karplus K, Li W, et al. Fast, scalable generation of high-quality protein multiple sequence alignments using Clustal Omega. Molecular Systems Biology. 2011;7: 539. doi:10.1038/msb.2011.75

53. Tamura K, Stecher G, Kumar S. MEGA11: Molecular Evolutionary Genetics Analysis Version 11. Molecular Biology and Evolution. 2021;38: 3022–3027. doi:10.1093/molbev/msab120

54. Kozlov AM, Darriba D, Flouri T, Morel B, Stamatakis A. RAxML-NG: a fast, scalable and user-friendly tool for maximum likelihood phylogenetic inference. Wren J, editor. Bioinformatics. 2019;35: 4453–4455. doi:10.1093/bioinformatics/btz305

55. Darriba D, Posada D, Kozlov AM, Stamatakis A, Morel B, Flouri T. ModelTest-NG: A New and Scalable Tool for the Selection of DNA and Protein Evolutionary Models.

Crandall K, editor. Molecular Biology and Evolution. 2020;37: 291–294. doi:10.1093/molbev/msz189

56. Manni M, Berkeley MR, Seppey M, Zdobnov EM. BUSCO: Assessing Genomic Data Quality and Beyond. Current Protocols. 2021;1: e323. doi:10.1002/cpz1.323

57. Labun K, Montague TG, Krause M, Torres Cleuren YN, Tjeldnes H, Valen E. CHOPCHOP v3: expanding the CRISPR web toolbox beyond genome editing. Nucleic Acids Research. 2019;47: W171–W174. doi:10.1093/nar/gkz365

58. Kistler KE, Vosshall LB, Matthews BJ. Genome Engineering with CRISPR-Cas9 in the Mosquito Aedes aegypti. Cell Reports. 2015;11: 51–60. doi:10.1016/j.celrep.2015.03.009

59. Tamura T, Thibert C, Royer C, Kanda T, Abraham E, Kamba M, et al. Germline transformation of the silkworm Bombyx mori L. using a piggyBac transposon-derived vector. Nat Biotechnol. 2000;18: 81–84. doi:10.1038/71978

60. Chen S, Zhou Y, Chen Y, Gu J. fastp: an ultra-fast all-in-one FASTQ preprocessor. Bioinformatics. 2018;34: i884–i890. doi:10.1093/bioinformatics/bty560

61. Kim D, Paggi JM, Park C, Bennett C, Salzberg SL. Graph-based genome alignment and genotyping with HISAT2 and HISAT-genotype. Nat Biotechnol. 2019;37: 907–915. doi:10.1038/s41587-019-0201-4

62. Kovaka S, Zimin AV, Pertea GM, Razaghi R, Salzberg SL, Pertea M. Transcriptome assembly from long-read RNA-seq alignments with StringTie2. Genome Biology. 2019;20: 278. doi:10.1186/s13059-019-1910-1

63. Patro R, Duggal G, Love MI, Irizarry RA, Kingsford C. Salmon provides fast and bias-aware quantification of transcript expression. Nat Methods. 2017;14: 417–419. doi:10.1038/nmeth.4197

64. Hirayama C, Konno K, Shinbo H. The pathway of ammonia assimilation in the silkworm, Bombyx mori. Journal of Insect Physiology. 1997;43: 959–964. doi:10.1016/S0022-1910(97)00045-0

65. Seibt KM, Schmidt T, Heitkam T. FlexiDot: highly customizable, ambiguity-aware dotplots for visual sequence analyses. Hancock J, editor. Bioinformatics. 2018;34: 3575–3577. doi:10.1093/bioinformatics/bty395

66. Zimin AV, Marçais G, Puiu D, Roberts M, Salzberg SL, Yorke JA. The MaSuRCA genome assembler. Bioinformatics. 2013;29: 2669–2677. doi:10.1093/bioinformatics/btt476

67. Shen W, Le S, Li Y, Hu F. SeqKit: A Cross-Platform and Ultrafast Toolkit for FASTA/Q File Manipulation. PLOS ONE. 2016;11: e0163962. doi:10.1371/journal.pone.0163962

68. Wickham H. ggplot2: Elegant Graphics for Data Analysis. New York, NY: Springer; 2009. doi:10.1007/978-0-387-98141-3

69. Teufel F, Almagro Armenteros JJ, Johansen AR, Gíslason MH, Pihl SI, Tsirigos KD, et al. SignalP 6.0 predicts all five types of signal peptides using protein language models. Nat Biotechnol. 2022;40: 1023–1025. doi:10.1038/s41587-021-01156-3

70. Robinson JT, Thorvaldsdóttir H, Winckler W, Guttman M, Lander ES, Getz G, et al. Integrative genomics viewer. Nat Biotechnol. 2011;29: 24–26. doi:10.1038/nbt.1754

71. Letunic I, Bork P. Interactive Tree Of Life (iTOL) v5: an online tool for phylogenetic tree display and annotation. Nucleic Acids Research. 2021;49: W293–W296. doi:10.1093/nar/gkab301

72. Kawahara AY, Plotkin D, Espeland M, Meusemann K, Toussaint EFA, Donath A, et al. Phylogenomics reveals the evolutionary timing and pattern of butterflies and moths.

Proceedings of the National Academy of Sciences. 2019;116: 22657–22663. doi:10.1073/pnas.1907847116

## Supplementary materials

**S1 Fig. Photographs of representative cocoons of the wild silkworm, *Bombyx mandarina*.**

A fresh mulberry leaf and cocoons of the wild silkworm in a bright field (left) and irradiated with ultraviolet light A in a dark field (right). The cocoons under ultraviolet irradiation exhibit fluorescence characteristic of quercetin-5-*O*-glucoside and quercetin-5,4´-di-*O*-glucoside, the major quercetin metabolites in the silkworm tissues and cocoon [14,15,28]. The cocoons were collected in June 2023 at Tsukuba, Ibaraki, Japan. Bar = 10 mm.

**S2 Fig. Phylogenetic tree of GH1 proteins in *B. mori* and other Holometabola insects.**

Values on the nodes represent the bootstrap score (trials = 100). Unreliable nodes with bootstrap values under 50 are shown as multi-branching nodes. Arabidopsis thioglucoside glucohydrolase 1 (TGG1) was used as the outgroup.

**S3 Fig. Similarity of the group G glycoside hydrolase proteins in *B. mori*.**

(A) Alignment of the group G glycoside hydrolase proteins in *B. mori* using Clustal Omega. Predicted signal peptides are highlighted by red lines. The threshold for identity shading was set to 80%. (B) Sequence identity and similarity of the group G glycoside hydrolase proteins in *B. mori*. Similarity of amino acid residue property is determined according to the groups of strongly similar properties described at Clustal Omega FAQ (https://www.ebi.ac.uk/seqdb/confluence/display/THD/Help+-+Clustal+Omega+FAQ).

**S4 Fig. Expression profile of the candidate genes within the *Gd* locus in third-day final instar larva of the p50T strain.**

All organs and tissues, except for ovaries, were taken from male larvae. Data are means of independent biological replicates ± SD. SG, silk gland; TPM, transcripts per million.

**S5 Fig. Total flavonoid content in cocoons of the p50T strain and *KWMTBOMO12227*-knockout lineages reared on fresh mulberry leaves.**

Data are means of independent biological replicates ± SD.

**S6 Fig. RNA-seq read alignment to the genomic sequence of *GH1G5*.**

(A) Regions around the translation start site (up) and stop site (down) of the cloned transcript sequence of *GH1G5*. The annealing sites of primers used for cloning are highlighted by black lines. (B) Mapping results of the midgut-derived RNA-seq reads and *GH1G5* transcript isoforms visualized with Integrative Genomics Viewer. Coverage on each exon and splice junction of three biological replicates is illustrated on tracks R1–3. (C) Coverage mean of each base of exon 10 and the 15-bp extension region. Data are means of independent biological replicates ± SD.

**S7 Fig. Weight of pupae and cocoons of p50T and *GH1G5* knockout mutants.**

(A) Weight of the pupae. (B) Weight of the cocoons. The values above the graph are *p*-values calculated by Student's *t*-test. Crosses inside the boxes indicate means of independent biological replicates.

**S8 Fig. Comparison of *GH1G5* sequences between the p50T and J01 strains.**

(A) Dot-plot analysis of the genomic region of exons 5, 6 and the intronic region between them (intron 5) of *GH1G5* from the p50T and J01 strains. Green dots indicate inverted regions. (B) Alignment of predicted protein sequences of GH1G5 from the p50T and J01 strains using Clustal Omega. (C) Deletion in the J01 *GH1G5* transcript confirmed using Sanger sequencing.

**S9 Fig. Alignment of GH1G5-like proteins in Macroheterocera species.**

(A) Whole alignment of 101 GH1G5-like proteins in Macroheterocera species. Amino acid residues identical to GH1G5 at positions with >99% conservation are highlighted by blue. The 100 homologous proteins were collected by a BLASTp search using the amino acid sequence of GH1G5 from sequence data of each protein from *Bombyx mori* (KWMT), *Antheraea yamamai* (Ayam), *Deilephila porcellus* (Dpor), *Dendrolimus punctatus* (Dpun) and *Pheosia gnoma* (Pgno) with a cutoff criterion e-value of <1E-100. The sequence data of *B. mori* were obtained from KAIKObase (https://kaikobase.dna.affrc.go.jp/index.html) and those of *A. yamamai*, *D. porcellus* and *P. gnoma* were obtained from InsectBase 2.0 (http://v2.insect-genome.com/). The sequences were aligned using Clustal Omega. (B) Magnified view of the J01-type deletion region of the alignment. Descriptions of the consensus symbols indicating the conservation level of amino acid residues are available at the Clustal Omega FAQ (https://www.ebi.ac.uk/seqdb/confluence/display/THD/Help+-+Clustal+Omega+FAQ). In brief, ':' indicates residues harboring a strongly similar physicochemical property to GH1G5, and '.' indicates residues harboring a weakly similar physicochemical property to GH1G5.

**S10 Fig. Genotyping of the *Gd* locus of Japanese or Chinese local white-cocoon strains.**

Haplotypes of the strains were determined by PCR genotyping. P/P, only the p50T genotype was detected; P/J/K, all p50T, J01, and Kosetsu genotypes were detected; J/J, only the J01 genotype was detected; J/K, both the J01 and Kosetsu genotypes were detected; K/K, only the Kosetsu genotype was detected; U, unknown, nothing detected. The primer set amplifying the genomic region from exons 1 to 2 of *KWMTBOMO14639* (*rp49*) was used as a control (predicted fragment length = 1548 bp). The straight grey dotted lines connect band markers of known size (M) which were applied at both ends of the samples. These lines represent sizes of 4000, 200, 500, and 1500 bp, from top to bottom. "JL" and "CL" stand for "Japanese local" and "Chinese local". The colors indicate the color of the cocoons.

**S1 Table. Information of the F2 individuals, parents and phenotypic score used for the QTL analysis.**

**S2 Table. QTLs associated with flavonoid content in cocoons.**

**S3 Table. Composition of the semi-synthetic diets supplemented with quercetin or rutin.**

**S4 Table. Orthogroups of glycoside hydrolases in the 12 species of Lepidoptera and numbers of detected gene duplication events.**

**S5 Table. Domestic silkworm glycoside hydrolases.**

**S6 Table. Summary of the Japanese and Chinese local silkworm strains examined.**

**S7 Table. Primers used in the experiments.**

**S1 Data. Data underlying the reported results.**

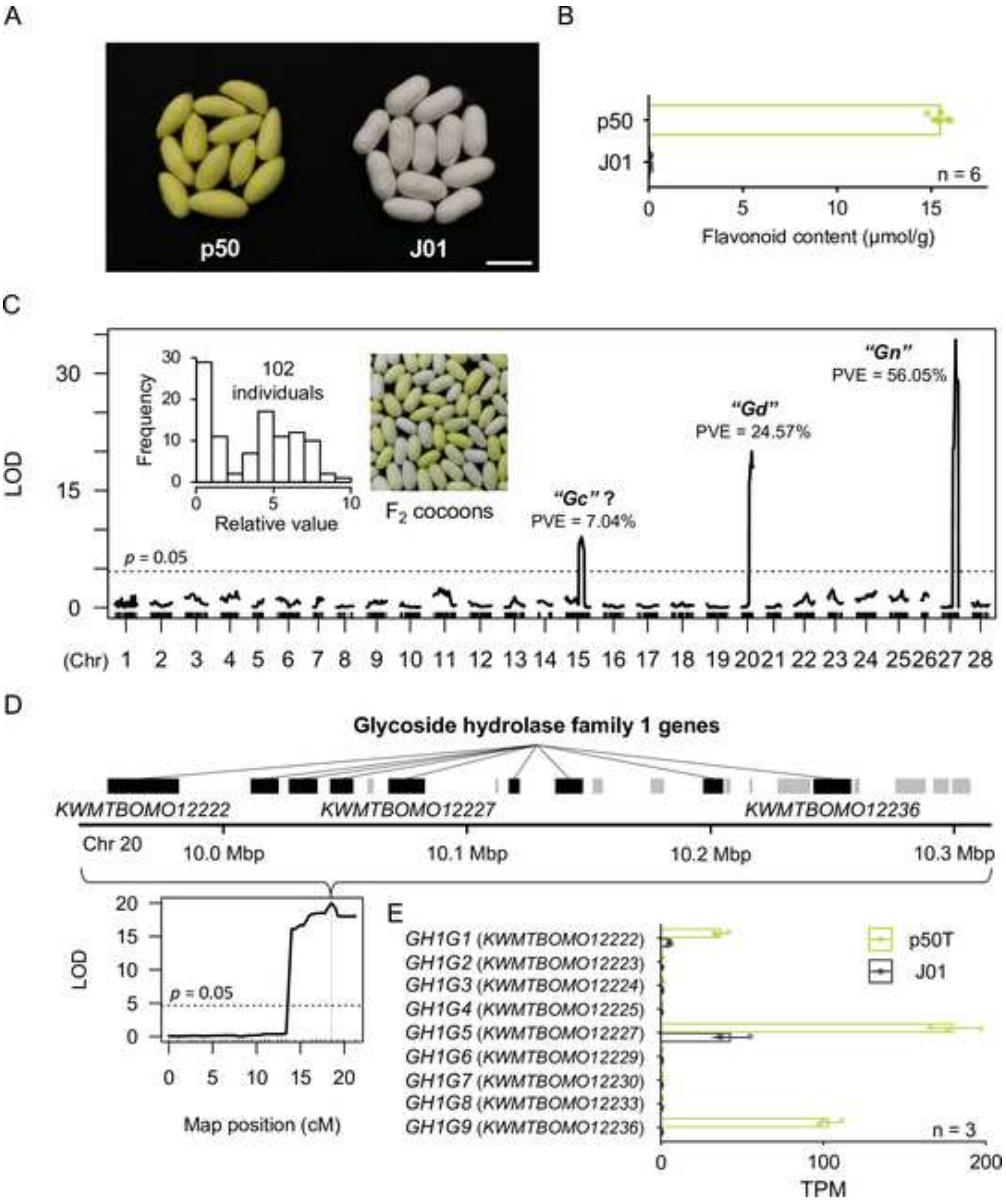
A
p50
J01
B
p50
J01
n = 6
0 5 10 15
Flavonoid content (μmol/g)
C
LOD
30
15
0
Frequency
102 individuals
Relative value
F2 cocoons
"Gc" ?
PVE = 7.04%
"Gd"
PVE = 24.57%
"Gn"
PVE = 56.05%
p = 0.05
(Chr) 1 2 3 4 5 6 7 8 9 10 11 12 13 14 15 16 17 18 19 20 21 22 23 24 25 26 27 28
D
Glycoside hydrolase family 1 genes
KWMTBOMO12222
KWMTBOMO12227
KWMTBOMO12236
Chr 20
10.0 Mbp
10.1 Mbp
10.2 Mbp
10.3 Mbp
LOD
p = 0.05
Map position (cM)
E
GH1G1 (KWMTBOMO12222)
GH1G2 (KWMTBOMO12223)
GH1G3 (KWMTBOMO12224)
GH1G4 (KWMTBOMO12225)
GH1G5 (KWMTBOMO12227)
GH1G6 (KWMTBOMO12229)
GH1G7 (KWMTBOMO12230)
GH1G8 (KWMTBOMO12233)
GH1G9 (KWMTBOMO12236)
p50T
J01
n = 3
0 100 200
TPM

Main figure 2

Click here to access/download;Figure;Fig2.tif

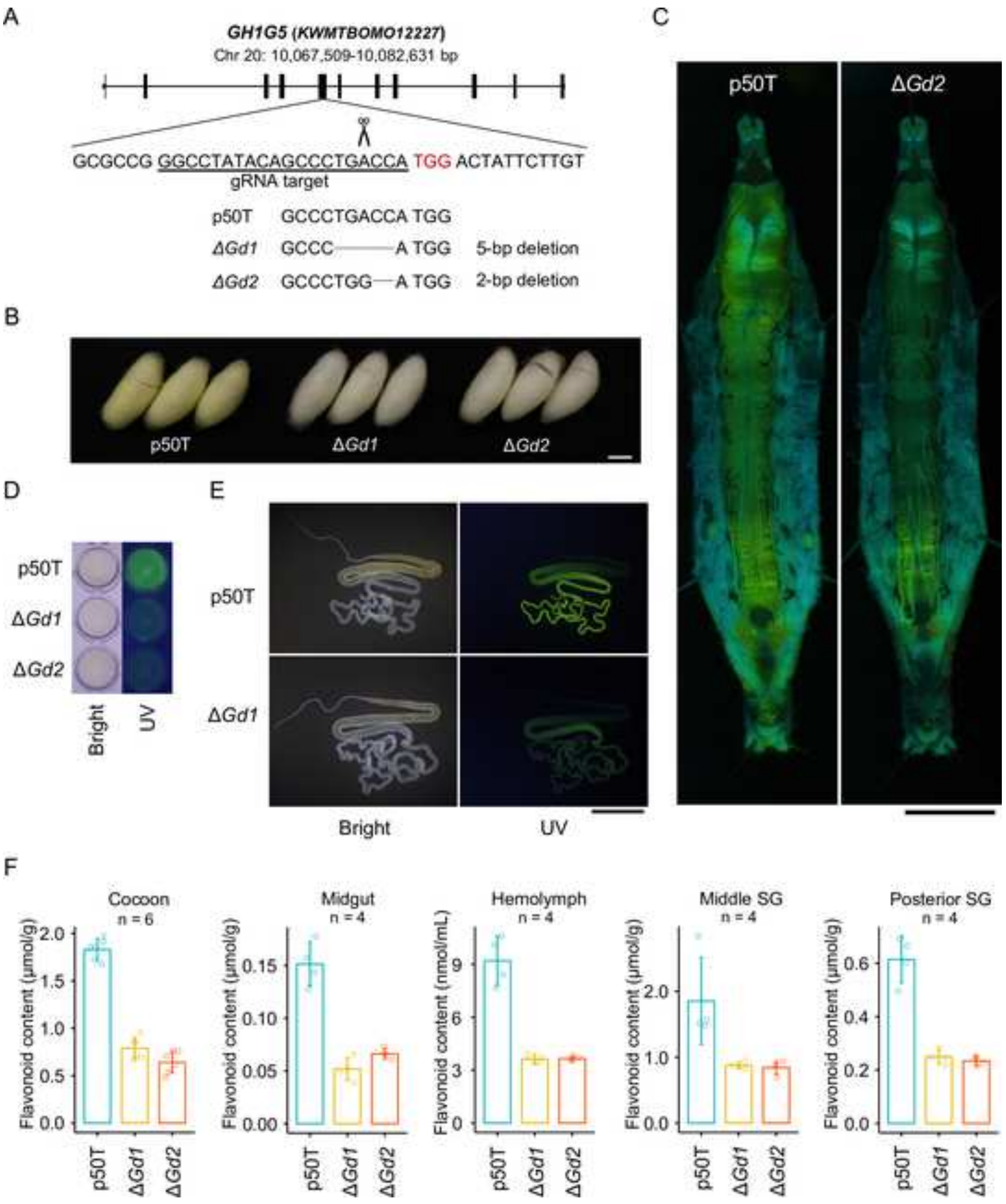

Main figure 3

Click here to access/download;Figure;Fig3.tif

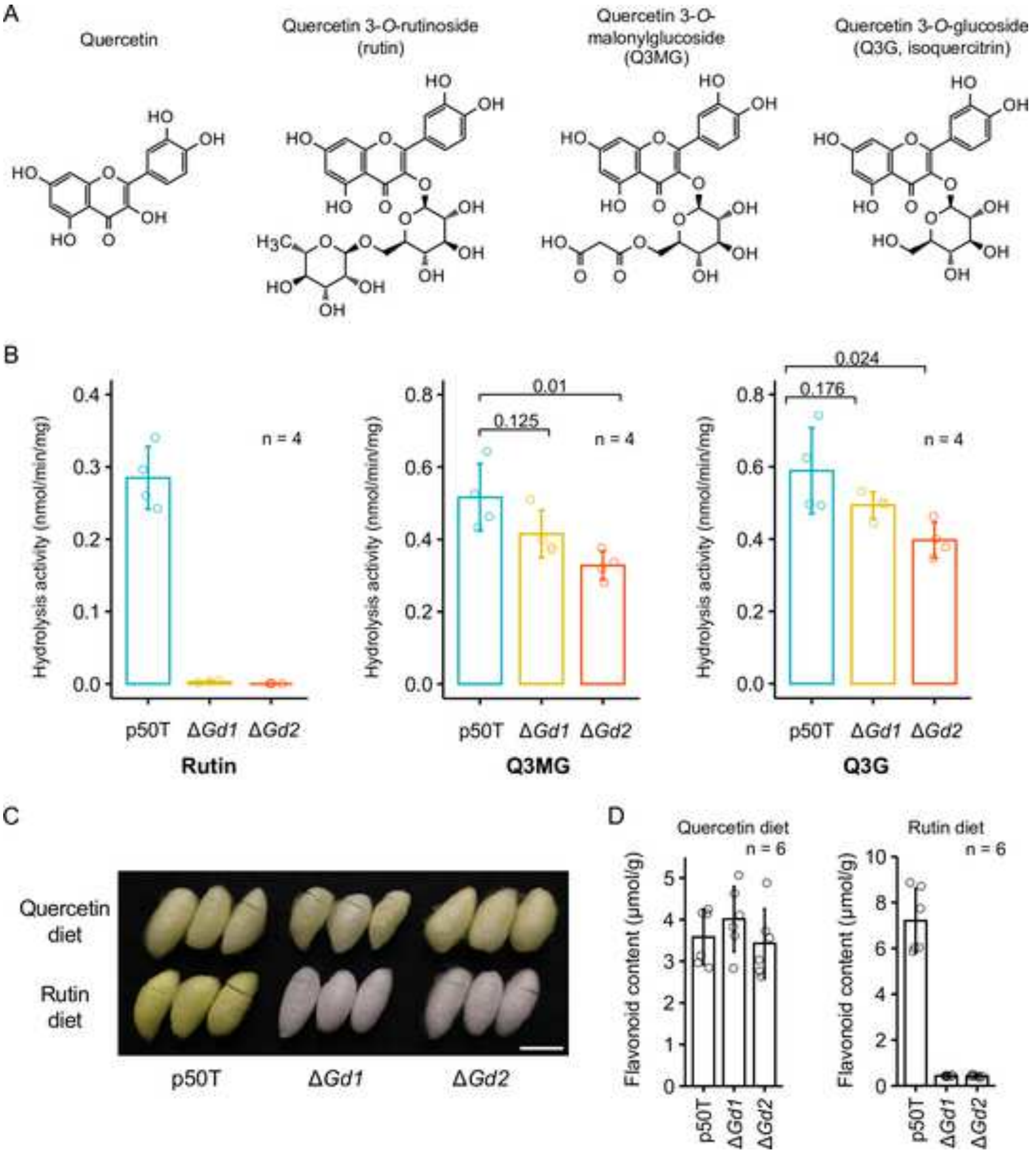

Main figure 4

Click here to access/download;Figure;Fig4.tif

A

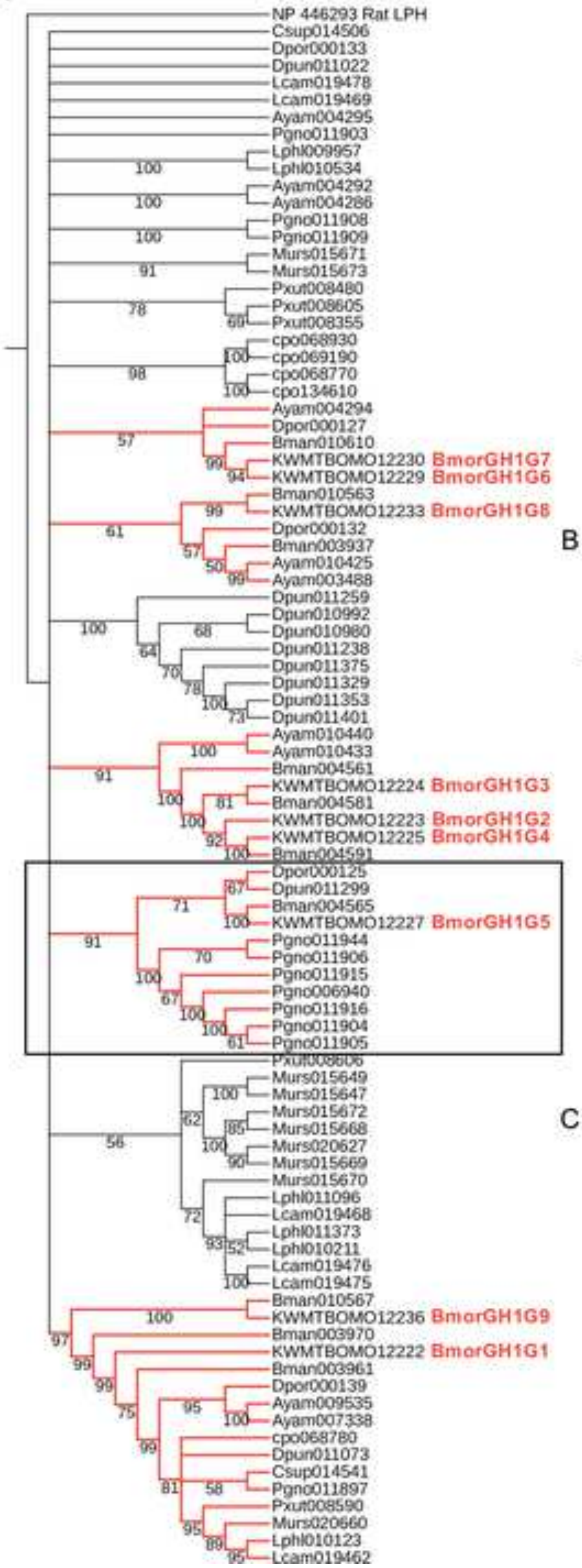


B

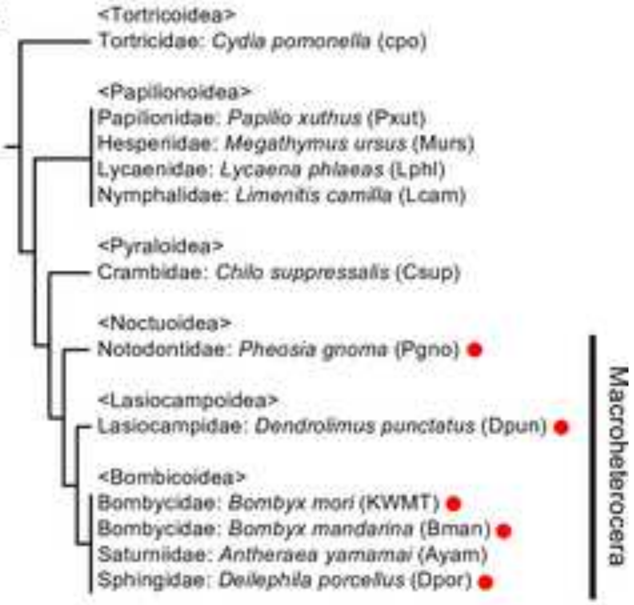


C

| | Identity | Similarity |
|---|---|---|
| Bman004565 | 98.58 | 99.59 |
| Dpor000125 | 55.23 | 89.15 |
| Dpun011299 | 51.20 | 82.80 |
| Pgno006940 | 51.29 | 86.93 |
| Pgno011904 | 48.15 | 80.00 |
| Pgno011905 | 50.49 | 86.39 |
| Pgno011906 | 55.07 | 87.87 |
| Pgno011915 | 53.63 | 87.03 |
| Pgno011916 | 52.09 | 85.88 |
| Pgno011944 | 57.92 | 89.98 |

Main figure 5

Click here to access/download;Figure;Fig5.tif

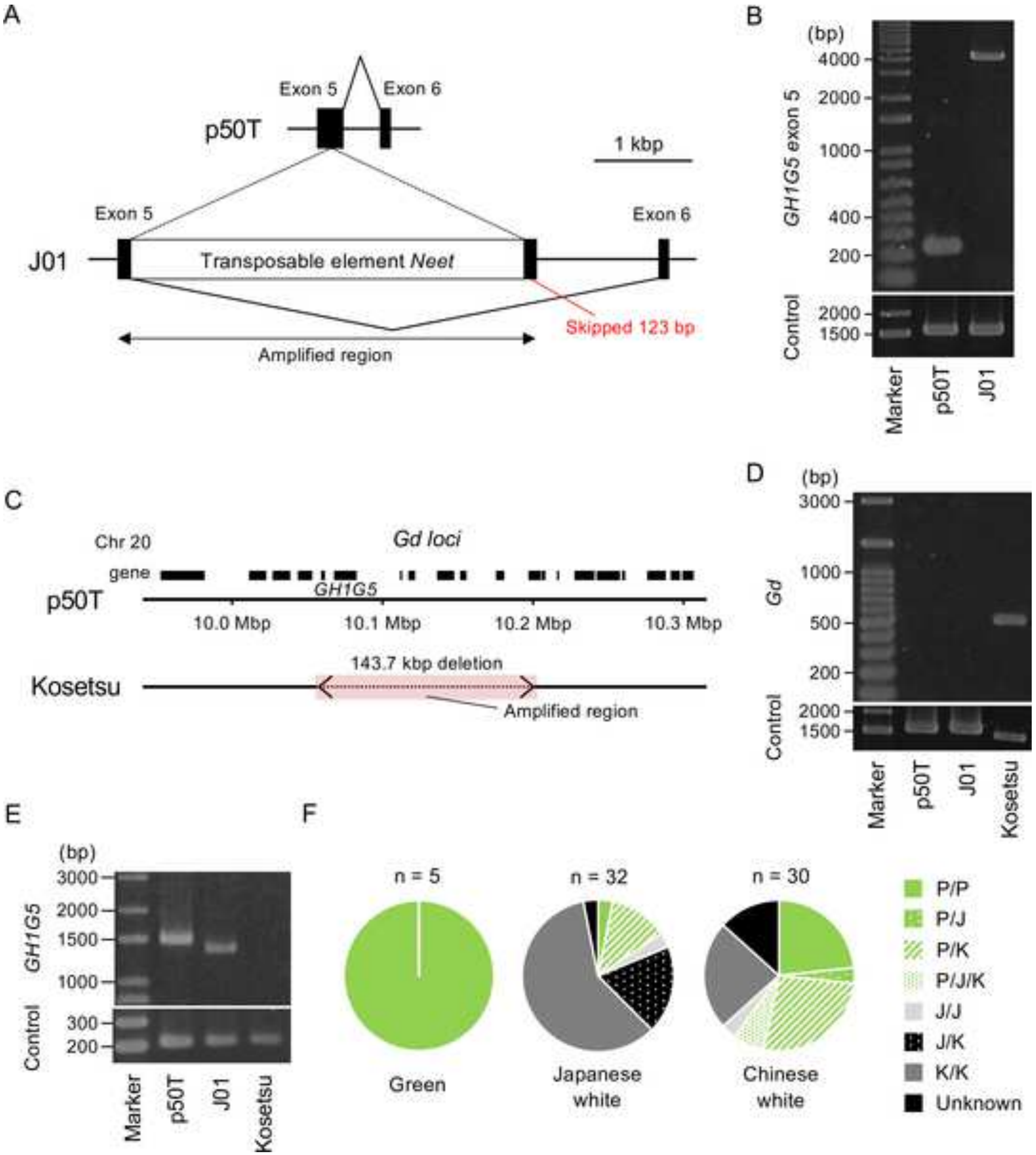

Click here to access/download;Figure;Fig6.tif

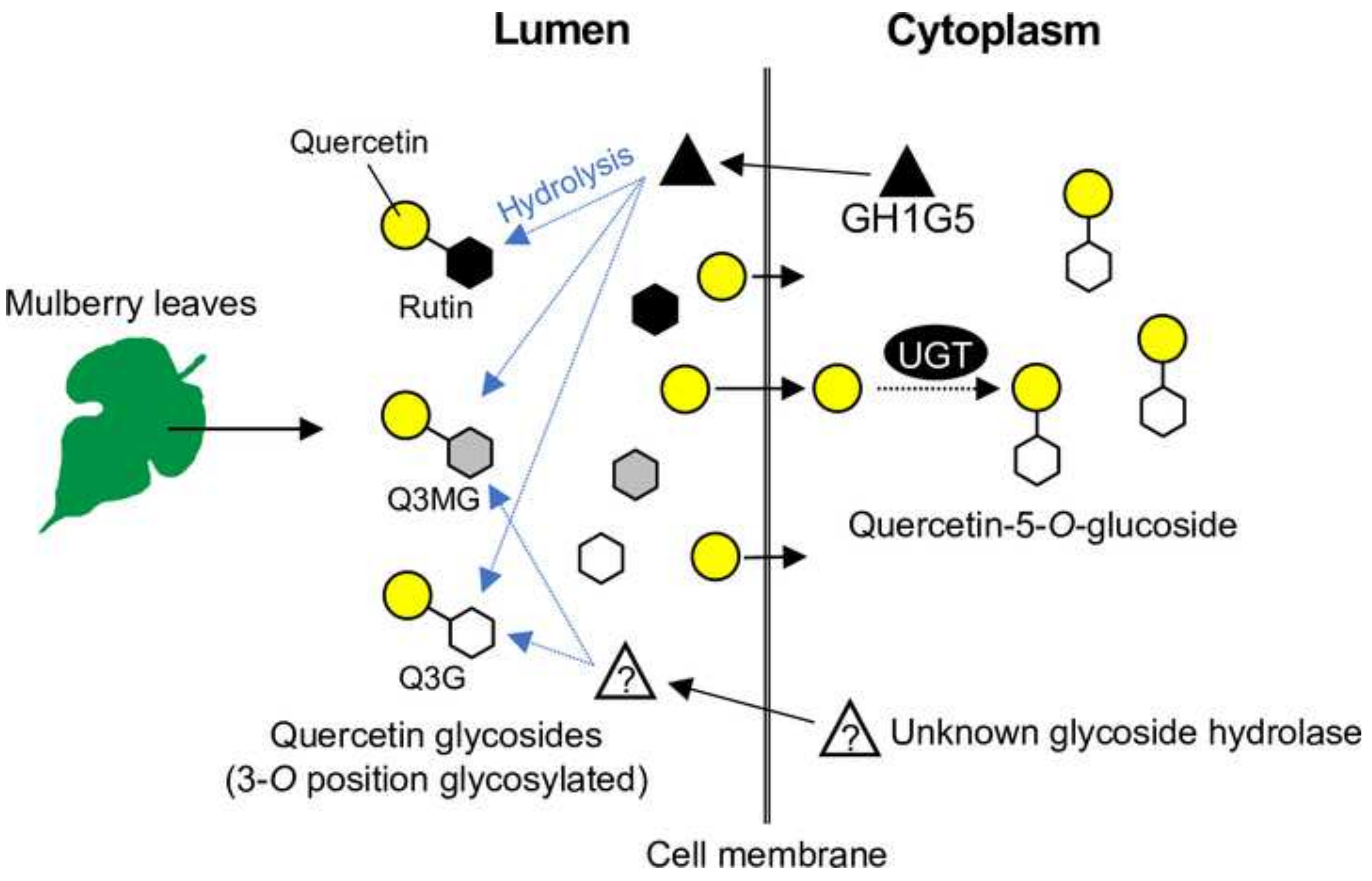

Supplementary figure 1

Click here to access/download
**Supporting Information**
S1Fig.tif

Supplementary figure 2

Click here to access/download
**Supporting Information**
S2Fig.tif

Supplementary figure 3

Click here to access/download
**Supporting Information**
S3Fig.tif

Supplementary figure 4

Click here to access/download
**Supporting Information**
S4Fig.tif

Supplementary figure 5

Click here to access/download
**Supporting Information**
S5Fig.tif

Supplementary figure 6

Click here to access/download
**Supporting Information**
S6Fig.tif

Supplementary figure 7

Click here to access/download
**Supporting Information**
S7Fig.tif

Supplementary figure 8

Click here to access/download

**Supporting Information**

S8Fig.tif

Supplementary figure 9

Click here to access/download
**Supporting Information**
S9Fig.tif

Supplementary figure 10

Click here to access/download
**Supporting Information**
S10Fig.tif

Supplementary tables S1–S7

Click here to access/download
**Supporting Information**
S1_S7_Table.xlsx

S1 Data: data underlying the reported results

Click here to access/download
**Supporting Information**
S1_Data.xlsx

# A major endogenous glycoside hydrolase mediating quercetin uptake in *Bombyx mori*

Deleted: glycosidase

Short title: GH1G5 mediates quercetin uptake in *Bombyx mori*

Deleted: LQGH1

Ryusei Waizumi*, Chikara Hirayama, Shuichiro Tomita, Tetsuya Iizuka, Seigou Kuwazaki, Akiya Jouraku, Takuya Tsubota, Kakeru Yokoi, Kimiko Yamamoto, Hideki Sezutsu

Institute of Agrobiological Sciences, National Agriculture and Food Research Organization (NARO), 1-2 Owashi, Tsukuba, Ibaraki 305-8634, Japan

*Correspondence:

Ryusei Waizumi

waizumir957@affrc.go.jp

**Abstract**

Quercetin is a common plant flavonoid which is involved in herbivore–plant interactions. Mulberry silkworms (domestic silkworm, *Bombyx mori*, and wild silkworm, *Bombyx mandarina*) take up quercetin from mulberry leaves and accumulate the metabolites in the cocoon, thereby improving its protective properties. Here we identified a glycoside hydrolase, named glycoside hydrolase family 1 group G 5 (GH1G5), which is expressed in the midgut and is involved in quercetin metabolism in the domestic silkworm. Our results suggest that this enzyme mediates quercetin uptake by deglycosylating the three primary quercetin glycosides present in mulberry leaf: rutin, quercetin-3-*O*-malonyl-glucoside, quercetin-3-*O*-glucoside. Despite being located in an instable genomic region that has undergone frequent structural changes in the evolution of Lepidoptera, GH1G5 has retained its hydrolytic activity, suggesting quercetin uptake has adaptive significance for the mulberry silkworms. *GH1G5* is also important in breeding: defective mutations which result in discoloration of the cocoon and increased silk yield are homozygously conserved in 27 of the 32 Japanese white-cocoon domestic silkworm strains and 12 of the 30 Chinese ones we investigated.

**Deleted:** that

**Deleted:** uptake

**Deleted:** and characterized

**Deleted:** glycosidase

**Deleted:** LPH-like quercetin glycoside hydrolase 1 (LQGH1)…

**Deleted:** that

**Deleted:** initiates quercetin metabolism in the domestic silkworm. LQGH1

**Deleted:** where

**Deleted:** it

**Deleted:** most commo

**Deleted:** n

**Deleted:** an unequal crossing-over hotspot, *LQGH1* is conserved in some species in the clade Macroheterocera

**Deleted:** which including includes the wild silkworm, and

**Deleted:** indicating

**Deleted:** indica

**Deleted:** es

**Deleted:** the

**Deleted:** of quercetin uptake

**Deleted:** *LQGH1*

**Deleted:** also

**Deleted:** of *LQGH1*,

**Deleted:** ,

## Author summary

Quercetin is one of the most abundant flavonoids present in plants. This flavonoid is involved in herbivorous insect–plant interactions. Insects utilize it for host–plant recognition, coloration, and protection from ultraviolet and oxidative stress. However, the molecular mechanism of quercetin metabolism in insects remains unclear. Mulberry silkworms (domestic silkworm, *Bombyx mori*, and wild silkworm, *Bombyx mandarina*) take up quercetin from mulberry leaves and sequester it into their cocoon to improve its protective properties. In this study, we identified an endogenous glycoside hydrolase in the domestic silkworm, named glycoside hydrolase family 1 group G 5 (GH1G5). This enzyme mediates quercetin uptake into the midgut cells by deglycosylating mulberry leaf-derived quercetin glycosides. This is the first discovery of a rutin glycoside hydrolase in an animal. Furthermore, we found that defective mutations of *GH1G5* have been broadly disseminated within the domestic silkworm population due to the improved cocoon color (i.e., discoloration to white) and increased silk yield. This study illuminates the unique mechanism of quercetin uptake in the domestic silkworm and uncovers an important event in the history of silkworm breeding.

**Deleted:** uptake

**Deleted:** of the domestic silkworm

**Deleted:** glycosidase

**Deleted:** LPH-like quercetin glycoside hydrolase 1 (LQGH1…

**Deleted:** )

**Deleted:** ,

**Deleted:** that

**Deleted:** which

**Deleted:** glycosidase

**Deleted:** *LQGH1*

**Deleted:** they produce

## Introduction

Quercetin (3,3',4',5,7-pentahydroxyflavone) is a flavonoid abundantly found in a wide variety of plants [1]. Previous studies have found quercetin glycosides possess oviposition and feeding stimulant activity in lepidopteran, orthopteran and coleopteran insects [2,3,4,5,6]. These observations suggest that quercetin is widely ingested by insects. Quercetin ingestion by insects is likely not merely a consequence of the identification and feeding on host plants; it may have adaptive significance. In the common blue butterfly (*Polyommatus icarus*), which sequesters quercetin-3-*O*-galactoside in its wings, flavonoid content is higher in the female than in the male and positively correlated with female sex attraction [7,8,9]. The yellow pigment in the wings of a grasshopper (*Dissosteira carolina*), which may contribute to their camouflage in plants, results from sequestration of quercetin-3-*O*-glucoside (isoquercitrin, Q3G) [10]. Although these studies strongly emphasize the broad significance of quercetin in herbivore–plant interactions, the underlying molecular mechanisms of quercetin metabolism in insects are poorly understood.

**Deleted:** oviposition and feeding stimulant activity of
**Deleted:** on
**Deleted:** examples/
**Deleted:** /findings
**Deleted:** insects
**Deleted:** s
**Deleted:** possibly
**Deleted:** s
**Deleted:** is the result of
**Deleted:** ?

Mulberry silkworms (domestic silkworm, *Bombyx mori*, and wild silkworm, *Bombyx mandarina*) accumulate various compounds derived from plants, including quercetin glucosides, kaempferol glucosides, and carotenoids, in their silk glands and colored cocoons (S1 Fig) [11,12,13]. Quercetin glucosides stored in the body and in the cocoon are reported to have antioxidant, ultraviolet-protective, and antibacterial properties [14,15,16]. Quercetin is found in the leaves of the mulberry tree (*Morus alba*), the sole food source of the mulberry silkworm, as a series of glycosides formed by glycosylation at the 3-*O* position. The three most common quercetin glycosides in mulberry are

**Deleted:** ?
**Deleted:** ?

quercetin-3-*O*-rutinoside (rutin), quercetin-3-*O*-malonyl-glucoside (Q3MG) and Q3G, which account for 71%–80% of the total flavonol content in the leaves [17].

The color of the cocoon of the domestic silkworm has been diversified through breeding, which indicates that the kinds and amounts of flavonoids that accumulate in the cocoons differ between strains [11,12]. Particularly, cocoons containing high flavonoid concentrations express a yellow-green color and are known as "green cocoons". Because accumulation in the cocoon is the end step of flavonoid metabolism, forward-genetic analysis focused on cocoon flavonoid content can reveal the genes involved in individual steps of flavonoid metabolism. Indeed, several loci associated with flavonoid metabolism have already been identified through this approach: the *Green b* locus, which encodes a uridine 5'-diphospho-glucosyltransferase (UGT) with a rare enzymatic activity glycosylating the 5-*O* position of quercetin [14], and the *New Green Cocoon* (*Gn*) locus, which encodes clustered sugar transporters presumed to import quercetin glucosides from the hemolymph to the silk gland [18]. However, these findings explain only a part of the process from quercetin uptake to the final accumulation of its metabolites in the cocoon. Elucidating the steps of flavonoid metabolism in the silkworm can provide valuable insights into the understanding of herbivore–plant interactions.

**Deleted:** in each

Here, we performed a quantitative trait locus (QTL) analysis focused on cocoon flavonoid content in the domestic silkworm and identified a novel locus, *Green d* (*Gd*), which is associated with this trait. Within the locus, we identified a glycoside hydrolase gene, glycoside hydrolase family 1 group G 5 (*GH1G5*), which mediates quercetin uptake into the midgut cells by deglycosylating mulberry leaf-derived quercetin glycosides. Genetic

**Deleted:** . We

**Deleted:** that

**Deleted:** cocoon flavonoid content

**Deleted:** /property

**Deleted:** glycosidase

**Deleted:** *LPH-like quercetin glycoside hydrolase 1* (*LQGH1*...

**Deleted:** )

**Deleted:** that

dissection of the novel gene revealed the contribution of the gene to improvement of the cocoon in a commercial context through breeding.

## Results

### QTL analysis identified a novel locus associated with flavonoid content in cocoon

To identify genes involved in quercetin metabolism by means of a forward-genetics approach, we prepared a green-cocoon strain (p50; alias: Daizo) and a white-cocoon strain (J01; alias: Nichi01). The two strains exhibited a distinct difference in cocoon color and flavonoid content (Fig 1A and 1B). A gradated range of cocoon colors in their F2 intercross offspring implied that the genetic differences between the two strains associated with flavonoid content were composite (Fig 1C). Therefore, we conducted a QTL analysis, which allows for simultaneous identification of multiple genetic loci involved in a phenotype of interest. The flavonoid content of the cocoons of the F2 population were scored by an absorbance-based method according to a previous report [19]. The QTL analysis was performed by using the phenotypic data of 102 individuals and 1038 genetic markers obtained from double-digest restriction-associated DNA sequencing data [20] (S1 Table). From the composite interval mapping, we identified three significant QTLs for cocoon flavonoid content on chromosomes 15, 20, and 27 (Fig 1C). Previous linkage studies have suggested a locus, named *Green c* (*Gc*) and associated with the yellow-green color of cocoons, at an unknown position on chromosome 15 [21,22]. The QTL on chromosome 27 was presumed to correspond to *Gn* [18]. Since no green cocoon-associated locus has yet been reported on chromosome 20, we named that locus *Green d* (*Gd*). The contribution of the *Gd* locus to cocoon flavonoid content was the second largest of the three QTLs, with a percentage of phenotypic variation explained

**Deleted:** s

**Deleted:** the

**Deleted:** gradated

**Deleted:** ing

**Deleted:** on chromosome 20

by each QTL (PVE) value of 24.57% (Fig 1C, S2 Table). The 95% Bayes credible interval of the *Gd* locus was 7,980,189–10,504,065 bp. The nearest marker to *Gd* was located at 10,265,033 bp, with a logarithm of odds (LOD) score of 19.99. The PVE values of the QTLs on chromosomes 15 and 27 were 7.04% and 56.05%. Significant additive effects were detected between the QTLs on chromosomes 15 and 27 and between those on chromosomes 20 and 27, with corresponding PVEs of 1.93% and 5.47% (S2 Table).

**Deleted:** , respectively

**Deleted:** ,

**Deleted:** and the

**Deleted:** were

**Deleted:** , respectively

**Fig 1. Quantitive trait loci (QTLs) associated with cocoon flavonoid content.**

(A) Photographs of representative cocoons of the p50 and J01 silkworm strains. Bar = 30 mm. (B) Flavonoid content of p50 and J01 cocoons. Data are means of independent biological replicates ± SD. (C) QTL analysis for cocoon flavonoid content. The horizontal dotted line indicates the threshold of the permutation test (trials = 1000). Phenotype scores on the frequency distribution are relative to a maximum measurement of 10. LOD, logarithm of odds; PVE, percentage of phenotypic variation explained by each QTL. According to a previous study [21], the *Gc* locus is located on chromosome 15, but its detailed genetic or physical position is unknown. Therefore, it cannot be concluded that the QTL peak on chromosome 15 identified here corresponds to the *Gc* locus. (D) Gene models present within the *Gd* locus of the p50T genome assembly. The genes annotated as encoding glycoside hydrolase family 1 proteins are highlighted in black; their IDs are *KWMTBOMO12222*, *KWMTBOMO12223*, *KWMTBOMO12224*, *KWMTBOMO12225*, *KWMTBOMO12227*, *KWMTBOMO12229*, *KWMTBOMO12230*, *KWMTBOMO12233*, and *KWMTBOMO12236* from the upstream side. (E) Expression of the candidate *Gd* genes in the midgut of third-day final instar male larvae. Data are means of independent biological replicates ± SD. TPM, transcripts per million.

**_Gd_ locus contains a glycoside hydrolase family 1 gene cluster**

Using the genome assembly and gene models of p50T [23], we found a cluster of nine genes encoding glycoside hydrolases (*KWMTBOMO12222–25*, *KWMTBOMO12227*, *KWMTBOMO12229*, *KWMTBOMO12230*, *KWMTBOMO12233*, and *KWMTBOMO12236*) on the *Gd* locus, which were annotated as glycoside hydrolase family 1 (GH1), according to the nomenclature of carbohydrate-active enzymes provided by CAZy [24] (Fig 1D). In mammals, a critical step in quercetin metabolism is deglycosylation of quercetin glucosides by lactase/phlorizin hydrolase (LPH), a member of GH1. LPH is expressed in intestinal epithelial cells and is anchored on the brush border membrane where it hydrolyzes flavonoid glycosides [25,26,27,28]. The resulting free quercetin aglycon is then passively absorbed into the intestinal cells due to its increased lipophilicity. We hypothesized that the *GH1* genes clustered within the *Gd* locus are involved in quercetin metabolism in the midgut lumen of the domestic silkworm, playing a role similar to that of LPH in mammals. In an inferred phylogenetic tree of all 21 GH1 proteins in *B. mori* and their homologous proteins in Holometabola insects (the fruit fly, *Drosophila melanogaster*; the honeybee, *Apis mellifera*; the red flour beetle, *Tribolium castaneum*), the glycoside hydrolases encoded in the *Gd* locus formed a distinct clade (group G), which was supported by a high bootstrap value (100%), suggesting that divergence of the group G-glycoside hydrolases had occurred after insect order divergence (S2 Fig). According to the phylogeny and their genomic positions, we named them as glycoside hydrolase family 1 group G 1–9 (GH1G1–9). The sequence identity among the group G glycoside hydrolase proteins was highest between GH1G2 and GH1G4 at 79.13%, and the sequence similarity was highest between GH1G1 and GH1G9 at 95.56% (S3A and S3B Fig). In addition, signal peptides were predicted in the N-

**Deleted:** **glycosidase**

**Deleted:** on the *Gd* locus

**Deleted:** deglycosylation of quercetin glucosides by lactase/phlorizin hydrolase (LPH), a member of beta-glucosidase family 1, is a

**Deleted:** beta-glucosidase family 1

**Formatted:** Not Highlight

**Deleted:** 4

**Deleted:** 5

**Deleted:** 6

**Deleted:** 7

**Deleted:** *glycosidase*

**Deleted:** s

terminal region of GH1G1, GH1G2, GH1G3, GH1G5, GH1G7 and GH1G9, suggesting they are secretory enzymes (S3A Fig). To identify which of them act in the midgut, we performed RNA-seq-based expression analysis. Three of the candidate genes, *GH1G1*, *GH1G5*, and *GH1G9*, were found to be strongly expressed in the midgut of p50T final instar larvae; the expression levels of these genes were significantly lower in the midgut of strain J01 (Fig 1E). Further examination of the expression profiles of the three genes with high expression in the midgut revealed that *GH1G5* and *GH1G9* were expressed specifically in the midgut, whereas *GH1G1* was expressed most in Malpighian tubules (S4 Fig). Taken together, we identified *GH1G1*, *GH1G5*, and *GH1G9* as candidate *Gd* genes which are involved in quercetin metabolism in the domestic silkworm.

**Deleted:** Gd glycosidases

**Deleted:** RNA-seq-based expression analysis

**Formatted:** Font: Not Italic

**Deleted:** was

**Deleted:** *KWMTBOMO12222*

**Deleted:** *KWMTBOMO12227*

**Deleted:** *KWMTBOMO12236*

**Deleted:** *KWMTBOMO12227*

**Deleted:** *KWMTBOMO12236*

**Deleted:** *KWMTBOMO12222*

**Deleted:** the

**Deleted:** m

**Deleted:** 2

**Deleted:** Thus

**Deleted:** *KWMTBOMO12222*

**Deleted:** *KWMTBOMO12227*

**Deleted:** *KWMTBOMO12236*

**Deleted:**

## Functional analysis of the candidate *Gd* genes by CRISPR-Cas9

To investigate the involvement of these candidate genes in quercetin metabolism, we attempted to use a microinjection-mediated CRISPR-Cas9 system to establish p50T lineages in which they had been knocked out. Although we failed to establish knockout lineages for *GH1G1* and *GH1G9* due to the lethality of homozygous frame-shift mutations, we did manage to obtain two knockout lineages of *GH1G5* with different types of frameshift mutations in exon 5. We designated the one with a 5-bp deletion as Δ*Gd1* and the other with a 2-bp deletion as Δ*Gd2* (Fig 2A). The *GH1G5* mutations resulted in a premature stop codon at exon 5 along with shortened amino acid sequence lengths from 492 to 215 in Δ*Gd1*, and from 492 to 216 in Δ*Gd2*. The mutants produced discolored cocoons compared to p50T (Fig 2B). In addition, we observed a reduction of fluorescence under ultraviolet irradiation the midgut, hemolymph, and silk glands of the mutants (Fig 2C–2E). Such fluorescence is characteristic of the accumulation of the two major quercetin metabolites in silkworm, quercetin-5-*O*-glucoside and quercetin-5,4′-di-*O*-glucoside [14,15,29]. Although knockout of *GH1G5* reduced the total flavonoid content in the cocoon by less than half that in the original p50T strain, it was still much larger than the effect predicted for the *Gd* locus in the QTL analysis (Fig 2F and S2 Table). We found similar reductions in the midgut, hemolymph, and middle and posterior silk gland. Because the flavonoid content in the cocoon differed largely between the insects reared with an artificial diet and those reared with fresh mulberry leaves (Fig 1B and Fig 2F), we confirmed the flavonoid content reductions in the mutants in an experiment using fresh mulberry leaves (S5 Fig). Together, these results indicated that knockout of *GH1G5* resulted in malfunction of quercetin uptake into the midgut.

**Deleted:** ¶
**Fig 1. Quantitive trait loci (QTLs) associated with cocoon flavonoid content.**¶
(A) Photographs of representative cocoons of the p50 and J01 silkworm strains. Bar = 30 mm. (B) Flavonoid content of p50 and J01the cocoons of p50 and J01. Data are means ± SD. (C) QTL analysis for cocoon flavonoid content. The horizontal dotted line indicates the threshold of the permutation test (trials = 1000). Phenotype scores on the frequency distribution are relative to a maximum measurement of 10. LOD, logarithm of odds; PVE, percentage of phenotypic variation explained by each QTL. According to a previous study [21], the *Gc* locus is located on chromosome 15, but the its detailed genetic or physical position is unknown. Therefore, it cannot be concluded that the QTL peak on chromosome 15 identified here corresponds to the *Gc* locus. (D) Modelled genes present within the *Gd* locus of the p50T genome assembly. The genes annotated as encoding a glycosyl hydrolase are highlighted in black; their IDs are *KWMTBOMO12222*, *KWMTBOMO12223*, *KWMTBOMO12224*, *KWMTBOMO12225*, *KWMTBOMO12227*, *KWMTBOMO12229*, *KWMTBOMO12230*, *KWMTBOMO12233*, and ... [1]

**Deleted:** the

**Deleted:** candidate genes

**Deleted:** ,

**Deleted:** *KWMTBOMO12222*

**Deleted:** *KWMTBOMO12236*

**Deleted:** *KWMTBOMO12227*

**Deleted:** se

**Deleted:** KWMTBOMO12227

**Deleted:** and

**Deleted:** a

**Deleted:** of KWMTBOMO12227

**Deleted:** Further characterization revealed that compared to p50T t

**Deleted:** , which is characteristic of the accumulation of the two major quercetin metabolites in silkworm (i.e., que ... [2]

**Deleted:** was observed in

**Deleted:** , indicating reductions of flavonoid content in the mutant tissues

**Deleted:** )

**Deleted:** 8

**Deleted:** *KWMTBOMO12227*

**Deleted:** S

**Deleted:** were found

**Deleted:** were confirmed

**Deleted:** 3

**Deleted:** *KWMTBOMO12227*

**Deleted:** s

**Fig 2. Reduction of flavonoid content in *GH1G5*-knockout mutants.**

(A) Disrupted target sequences in *GH1G5*. (B)–(E) Cocoons (B), midguts (C), hemolymph (D), and silk glands (E) of the p50T strain and the *GH1G5* mutants. Organs and tissues were taken from sixth-day final instar female larvae. Bars = 10 mm. (F) Total flavonoid content of cocoons, organs, and tissues of the p50T strain and the *KWMTBOMO12227* mutant lineages. Data are means of independent biological replicates ± SD. All insects were reared on a commercial artificial diet. SG, silk gland.

**Deleted:** ***KWMTBOMO12227***

**Deleted:** *KWMTBOMO12227*

**Deleted:** *KWMTBOMO12227*

## Sequence and isoform determination for *GH1G5*

The predicted gene model for *GH1G5*, *KWMTBOMO12227*, consists of 11 exons, encoding a total of 492 amino acids. The accuracy of the modeled sequence and its dominance among isoforms were confirmed using Sanger sequencing and the RNA-seq data obtained from final instar larvae of strain p50T. We cloned the putative longest open reading frame in the transcripts of *GH1G5*, and sequenced it by Sanger sequencing (S6A Fig). The determined sequence was absolutely identical to the modeled sequence of *GH1G5*. Previously, our research group reported RNA-seq data for final instar larva tissues of the p50T strain [30]. By mapping the reads of the data derived from the midgut to the genomic sequence of the p50T strain reported by Kawamoto et al. [23], we determined the transcript isoforms of *GH1G5*. The gene model accurately represented the dominant isoform, and another isoform extending 15-bp downstream of the 10th exon was also detected (S6B Fig). The termination codon in the extended region shortened the predicted protein sequence length of this isoform to 456 amino acids. Since the mean coverage of each base of the 15-bp extended region was only about 15% of that of the original 10th exon of *GH1G5* (S6C Fig), we concluded this as a minor isoform.

## GH1G5 mediates quercetin uptake by hydrolysis of quercetin glycosides

Together, our knockout analysis and sequence characterization suggested that the GH1G5 is secreted into the midgut lumen and mediates the uptake of mulberry-derived quercetin by deglycosylation of quercetin glycosides. However, the knockout mutants still accumulated some flavonoids in the midgut cells (Fig 2F). This might be due to the diversity of quercetin glycosides in mulberry leaves and the substrate specificity of GH1G5. To confirm whether GH1G5 is involved in deglycosylation of quercetin

**Deleted: Characterization of the protein sequence of the glycosidase encoded by *KWMTBOMO12227***

**Deleted:** The CRISPR-Cas9-mediated knockout analysis strongly suggested that *KWMTBOMO12227* encodes a glycosidase which plays a crucial role in quercetin uptake in the domestic silkworm.

**Deleted:** as

**Deleted:** (S1 Text)

**Deleted:** KWMTBOMO12227 was found to have an amino acid sequence similar to that of LPH, but phylogenetic inference suggested that KWMTBOMO12227 is not orthologous to LPH; it has evolved through gene duplication after the divergence of Pyraloidea and Macroheterocera (including Noctuoidea, Lasiocampoidea, and Bombycoidea) (S1 Text). Mature LPH harbors a transmembrane domain in the C-terminus region with which it is anchored to the brush border membrane [25]. A hydrophobic transmembrane domain was also predicted in the first N-terminal 20 amino acid residues of KWMTBOMO12227 (S4 Fig), suggesting that the protein functions as a membrane-anchored glycosidase.…

**Deleted: LQGH1**

**Deleted:** glycosidase

**Deleted:** encoded by *KWMTBOMO12227*

**Deleted:** anchored on the apical surface of epithelial cells

**Deleted:** in the midgut of the silkworm

**Deleted:** some

**Deleted:** ion

**Deleted:** of

**Deleted:** was still observed in the knockout mutants

**Deleted:** ay

**Deleted:** KWMTBOMO12227

**Deleted:** *KWMTBOMO12227*

glycosides, we investigated the hydrolytic activity of midgut tissue of the knockout mutants on the three major quercetin glycosides in mulberry leaf: rutin, Q3MG, and Q3G (Fig 3A).

**Deleted:** investigated the effect of knocking out the gene on the hydrolytic activity of midgut tissue

**Deleted:** To also examine the localization of KWMTBOMO12227, we homogenized the midgut followed by centrifugation, and used the soluble homogenate homogenate and the its insoluble material fraction of that, including cell membranes, , isolated from the homogenate by centrifugation, were used separately for thefor separate measurements.

**Fig 3. Hydrolytic activity of GH1G5 on the three major quercetin glycosides in mulberry leaf.**

(A) Structural formulae of quercetin and the three major quercetin glycosides present in mulberry leaf. (B) Hydrolytic activity of the midgut on the quercetin glycosides. Data are means of independent biological replicates ± SD. The values above the graph are *p*-values calculated by a two-tailed Student's *t*-test. All samples were from fifth-day final instar female larvae reared on a commercial artificial diet. (C) Photograph of representative cocoons of the p50T strain and the mutants reared on semi-synthetic diets containing quercetin or rutin. Bar = 20 mm. (D) Total flavonoid content in cocoons of the p50T strain and the mutants reared on semi-synthetic diets containing quercetin or rutin. Data are means of independent biological replicates ± SD.

The homogenate of the p50T midgut exhibited hydrolytic activity against all three types of quercetin glycoside (Fig 3B). However, the activities were decreased in the knockout mutants, indicating that GH1G5 is involved in the hydrolysis of all three quercetin glycosides. Notably, *GH1G5* knockout completely abolished the hydrolytic activity against rutin, indicating it is the only glycoside hydrolase with hydrolytic activity against this quercetin glycoside. Furthermore, partial reductions were observed in the hydrolytic activity of the other two glycosides, Q3MG and Q3G, was only partial, suggesting that the silkworm has other additional glycosidses with hydrolytic activities against those molecules (Fig 3B). Knocking out *GH1G5* reduced the hydrolytic activity of midgut homogenates against Q3MG and Q3G by 0.6 to 0.8-fold and 0.7 to 0.8-fold, respectively. These results suggested that GH1G5 is an important protein for the hydrolysis of quercetin glycosides in the silkworm lumen.

In 1972, Fujimoto and Hayashiya reported that the domestic silkworm accumulates flavonoids in its cocoon when reared on artificial diets containing isolated quercetin or rutin [31]. To investigate whether the uptake of rutin-derived quercetin is dependent on deglycosylation by GH1G5, we reared insects on semi-synthetic diets supplemented with rutin or quercetin but without mulberry leaf powder and measured the flavonoid content in the cocoons. The p50T strain accumulated flavonoids in its cocoon irrespective of diet (Fig 3C, 3D, and S3 Table). Although the *GH1G5*-knockout mutants accumulated the same amount of flavonoids as did p50T when reared on the quercetin diet, they accumulated only 6% of that accumulated by p50T when reared on the rutin diet (Fig 3C and 3D). These results were consistent with the report by Fujimoto and Hayashiya, and

**Deleted:** KWMTBOMO12227

**Deleted:** *KWMTBOMO12227*

**Deleted:** that KWMTBOMO12227

**Deleted:** glycosidase

**Deleted:** rutin

**Deleted:** T

**Deleted:** Further, the hydrolytic activity of rutin was stronger in the insoluble fraction than in the homogenate, supporting our assertion that KWMTBOMO12227 is a membrane-anchored glycosidase. The

**Deleted:** a

**Deleted:** *KWMTBOMO12227*

**Deleted:** The reduction in

**Deleted:** knocking out *KWMTBOMO12227* was

**Deleted:** The

**Deleted:** Additionally, the reduction in the hydrolytic activity of the insoluble fraction against the two quercetin glycosides was 0.2 and 0.5-fold, respectively, which were was greater than those that of the homogenate.

**Deleted:** KWMTBOMO12227

**Deleted:** According to the characteristics of the protein sequence and enzymatic activity of KWMTBOMO12227, we named the identified gene *LQGH1*.

**Deleted:** but not when reared on mulberry leaves

**Deleted:** 29

**Deleted:** LQGH1

**Deleted:** *LQGH1*

**Deleted:** a

suggested that the uptake of quercetin from rutin is strongly dependent on deglycosylation by *GH1G5*.

Deleted: indicate

Deleted: *LQGH1*

## Evolution of GH1G5

The phylogeny of the Holometabola GH1 proteins suggested that the divergence of the group G  glycoside hydrolases had occurred after insect order divergence (S2 Fig). To estimate when *GH1G5* arose during the evolution of Lepidoptera, we constructed a maximum likelihood-inferred phylogenetic tree of lepidopteran-wide orthologous proteins of the group G glycoside hydrolases in *B. mori* (BmorGH1G1–9) (Fig 4A). The tree classified BmorGH1G1–9 into five clades: one clade including BmorGH1G5, one clade including BmorGH1G1 and BmorGH1G9, one clade including BmorGH1G2, BmorGH1G3 and BmorGH1G4, one clade including BmorGH1G6 and BmorGH1G7, and one clade including BmorGH1G8. The clade including BmorGH1G5 consisted of only proteins from species belonging to Macroheterocera (including Noctuoidea, Lasiocampoidea, and Bombycoidea), suggesting that *GH1G5* had evolved through gene duplication after the divergence of Pyraloidea and Macroheterocera (Fig 4A and 4B). Sequence identities and similarities of the GH1G5-class proteins, excluding the one from *B. mandarina*, to BmorGH1G5 were at a maximum of 57.92% and 89.98% (Fig 4C). OrthoFinder [32], the tool we used for the collection of orthologous proteins of BmorGH1G1–9, detected gene duplication events that had occurred within each orthologous group while simultaneously estimating orthologous relationships. Interestingly, it suggested that the group G glycoside hydrolases had undergone notably frequent duplication events; the estimated number of duplications which occurred within the ortholog group including GH1G5 (also including GH1G2, GH1G4, and GH1G6–9) was 46, which ranked 197th out of 19436 total groups and 1st out of 54 groups including *B. mori* proteins annotated as glycoside hydrolases (InterPro entry: IPR001360 or GO annotation: 0016798) (S4 and S5 Table).

**Deleted:** ¶
**Fig 3. Hydrolytic activity of KWMTBOMO12227 on the three major quercetin glycosides in mulberry leaf.**¶
(A) Structural formulae of quercetin and the three major quercetin glycosides present in mulberry leaf. (B) Hydrolytic activity of the midgut on the quercetin glycosides. Data are means ± SD. The values above the graph are *p*-values calculated by a two-tailed Student's *t*-test. The All samples used were all fifth-day final instar female larvae reared on a commercial artificial diet. (C) Photograph of representative cocoons of the p50T strain and the mutants reared on semi-synthetic diets containing quercetin or rutin. Bar = 20 mm. (D) Total flavonoid content in the cocoons of the p50T strain and the mutants reared on semi-synthetic diets containing quercetin or rutin. Data are means ± SD.¶
Page Break
¶

**Fig 4. Phylogenetic tree of lepidopteran-wide orthologous proteins of the group G glycoside hydrolases in *B. mori*.**

(A) The phylogenetic tree was constructed by the maximum likelihood method. Values on the nodes represent the bootstrap scores (trials = 100). Unreliable nodes with bootstrap values under 50 are shown as multi-branching nodes. The tree was rooted using Rat LPH as an outgroup. Red branches represent clades including the group G glycoside hydrolases in *B. mori*. The clade containing BmorGH1G5 is highlighted with a box. (B) Species tree of Lepidoptera drawn/constructed with reference to the report by Kawahara et al. [72]. Red circles indicate species harboring an GH1G5 ortholog. (C) Sequence identities and similarities of the GH1G5-class proteins to BmorGH1G5. Similarity of amino acid residue property is determined according to the groups of strongly similar properties described at Clustal Omega FAQ (https://www.ebi.ac.uk/seqdb/confluence/display/THD/Help+-+Clustal+Omega+FAQ).

**Defective structural mutations of *GH1G5* were broadly disseminated in white-cocoon domestic silkworm strains**

Although accumulation of carotenoids or flavonoids in the cocoon produces a variety of cocoon colors, it is white-cocoon strains that are the most popular in commercial use. Earlier in the present study, we found that defective mutations of *GH1G5* resulted in impaired quercetin absorption, which made cocoon colors closer to white (Fig 2B). Interestingly, the defective mutation of *GH1G5* did not impair the growth of the silkworm, but rather improved silk yield (S7 Fig). The implication here was that this beneficial mutation was selected for in the establishment of white-cocoon domestic silkworm strains.

To examine our hypothesis, we determined conserved *GH1G5* genotypes in a collection of *B. mori* strains, and investigated the frequency and distribution. We first determined the details of the mutation introduced in J01, and then compared them to p50T. Previously, we reported a genome assembly and gene model for J01 [33]. A BLASTp search of our J01 gene model identified predicted gene *BMN13127* as corresponding to *GH1G5* in this strain. Comparing the genomic regions of *GH1G5* in p50T and J01, an insertion of 3997 bp was found in exon 5 of J01 *GH1G5* (Fig 5A). The inserted sequence was identified as the reported non-autonomous transposable element in *B. mori*, *Neet* [34]. The *Neet* sequence and the intronic region between exon 5 and 6 in J01 included the inverted sequence of the intronic region between exon 5 and 6 of *GH1G5* of p50T (Fig S8A), suggesting that it had experienced additional structural changes. A product amplified from the genomic DNA region of exon 5 of J01 including the insertion was approximately 4000-bp longer than that from the p50T genomic DNA (Fig 5B). Probably due to the insertion, the predicted splice site of the exon 5 end shifted 123 bp forward in J01 *GH1G5*,

- **Deleted:** *LQGH1*
- **Deleted:** *LQGH1*
- **Deleted:** the color of the cocoons
- **Deleted:** *LQGH1*
- **Deleted:** 5
- **Deleted:** i
- **Formatted:** Font: Italic
- **Deleted:** looked at
- **Deleted:** of the defective mutations of *LQGH1* in a collection of *B. mori* strains
- **Deleted:** 0
- **Deleted:** the
- **Deleted:** gene of
- **Deleted:** *LQGH1*
- **Deleted:** J01
- **Deleted:** ?well-studied wild type?
- **Deleted:** *LQGH1*
- **Deleted:** in
- **Deleted:** the two strains
- **Deleted:** *LQGH1*
- **Formatted:** Font: Italic
- **Formatted:** Font: Italic
- **Formatted:** Font: Italic
- **Deleted:** Syntenic dot-plot analysis of the genomic sequences of exons 5, 6 and
- **Deleted:** them (intron 5)
- **Deleted:** *LQGH1*
- **Deleted:** the two strains revealed that the inserted region containeds the inverted sequence of intron 5 of
- **Deleted:** 4
- **Deleted:** .
- **Deleted:** The inverted intron sequence was present at two sites within intron 5 of J01 *LQGH1*.
- **Deleted:** In the genomic PCR amplifying the region on exon 5, including the insertion, an
- **Deleted:** amplified
- **Deleted:** J01
- **Deleted:** DNA
- **Deleted:** 4
- **Deleted:** Due to the insertion, the predicted *LQGH1* protein sequence of J01 LQGH1 lacked 31 amino acid residues in the middle compared to that of p50T LQGH1
- **Formatted:** Font: Italic

resulting in 41 amino acids deletion in the predicted protein sequence (Fig 5A and S8B Fig). By cloning and sequencing the J01 transcript using the primer sets that were used to determine the open reading frame sequence of p50T *GH1G5*, the accuracy of the modeled sequence was confirmed (S8C Fig). The deletion included two of nine residues with >99% conservation across 101 homologous proteins from five Macroheterocera species, and all of seven residues which harbored strongly similar physicochemical properties across all of these proteins (S9 Fig). These observations strongly suggested that the deletion was mainly responsible for the observed difference in flavonoid content in the cocoon.

**Deleted:** S

**Deleted:** 6

**Formatted:** Font: Italic

**Deleted:**

**Deleted:** that

**Deleted:** ?

**Deleted:** ?

**Deleted:** 7

**Deleted:** is

**Deleted:** ?

**Fig 5. Conserved structural mutations of *GH1G5* in the domestic silkworm population.**

(A) Schematic illustration comparing exon 5, 6 and the intronic region between them (intron 5) of *GH1G5* from the p50T and J01 strains. (B) PCR identifying the J01-type insertion. The amplified region is indicated by the colored area in (A). The primer set amplifying the genomic region from exons 1 and 2 of *KWMTBOMO14639* (*rp49*) was used as the control (predicted fragment length = 1548 bp). (C) Schematic illustration comparing the *Gd* loci of the p50T and Kosetsu strains. Black boxes indicate the modeled genes. (D) PCR identifying the Kosetsu-type large deletion. The amplified region is indicated by the colored area in (C). (E) RT-PCR of the *GH1G5* full-length open reading frame using cDNA libraries constructed from the midguts of sixth-day final instar larvae. The primer set targeting exons 1 and 2 of *KWMTBOMO14639* (*rp49*) was used as the control (predicted fragment length = 213 bp). (F) Distribution of the haplotypes of *GH1G5* in Japanese and Chinese local silkworm strains. P/P, only the p50T genotype was detected; P/J/K, p50T, J01, and Kosetsu genotypes were detected; J/J, only the J01 genotype was detected; J/K, both J01 and Kosetsu genotypes were detected; K/K, only the Kosetsu genotype was detected; U, unknown, nothing detected. Grayscale colors indicate pairs of loss-of-function haplotypes.

**Deleted:** Dot-plot analysis of the genomic region of

**Deleted:** s

**Deleted:** .

Next, we examined the presence of the J01-type 4-kbp insertion in 67 Japanese and Chinese local strains by PCR using tested primers (S10 Fig and S6 Table). All five green-cocoon strains lacked the insertion. Of the 32 Japanese local white-cocoon strains, the insertion was absent in five and present in seven, whereas the remaining 20 lacked any amplification product. Of the 30 Chinese local white-cocoon strains, the insertion was absent in 15, present in four (p50T-type products were also observed in three strains of them), and 11 again lacked an amplification product. The lack of an amplification product suggested these strains contained another dysfunctional mutation. To determine what it might be, we obtained Illumina whole-genome sequencing data of Kosetsu, one of the strains lacking an observed amplification product, and assembled it. By comparison against the genomic sequence of p50T, a 143.7-kbp deletion including *GH1G5* was found in the Kosetsu *Gd* locus (Fig 5C). When the region including the deletion was amplified by PCR using p50T, J01, and Kosetsu genomic DNA, an observable amplification product was synthesized only from the Kosetsu sample (Fig 5D). In addition, no observable amplification product was synthesized from the Kosetsu sample by using reverse-transcription PCR to amplify the full-length open reading frame of *GH1G5* using cDNA from the midgut of sixth-day final instar larvae of the three strains (Fig 5E). Moreover, the J01-derived product was shorter in size and the band appeared fainter than that from p50T. These results were consistent with the genetic differences of *Gd* among the three strains predicted from the assembled genomic sequences. Using the primer sets for detecting the Kosetsu-type large deletion, we examined the presence of the mutation in all of the prepared 67 silkworm strains. It revealed that 46 of the 67 strains possessed the Kosetsu-type deletion. Genotypes of 26 of the 20 Japanese and 11 Chinese local white-cocoon strains which we failed genotyping were determined as the Kosetsu-type,

**Deleted:** 8
**Deleted:** 4
**Deleted:** ,
**Deleted:** and
**Deleted:** no
**Deleted:** was observed in 20
**Deleted:** se strains
**Deleted:** no
**Deleted:** was observed in 11
**Deleted:** with
**Deleted:** the mutation
**Deleted:** assembled
**Deleted:** the genomic sequence
**Deleted:** without
**Deleted:** Kosetsu,
**Deleted:** using the available? Illumina whole-genome sequencing data [ref]
**Deleted:** *LQGH1*
**Deleted:** 4
**Formatted:** Font: Not Bold
**Deleted:** 4
**Deleted:** ing
**Deleted:** *LQGH1*
**Deleted:** 4
**Deleted:** The
**Deleted:** had a weaker signal compared to
**Deleted:** a

whereas the remaining five still lacked any amplification product (S10 Fig). The five strains which we failed genotyping using any of the used primer sets might have undergone further genomic structural changes after acquiring the Kosetsu-type large deletion.

To summarize the genomic PCR analysis, we found three haplotypes of *GH1G5* in the population: p50T-type (P, functional), J01-type (J, harboring a 4-kbp insertion in exon 5), and Kosetsu-type (K, harboring a large deletion eliminating *GH1G5*). The proportions of strains homozygous for the functional haplotype (P), heterozygous for the functional and a dysfunctional (J or K) haplotype, and homozygous for a dysfunctional haplotype were 3%, 13%, and 84% in the Japanese local white-cocoon strains, and 23%, 37%, and 40% in the Chinese local white-cocoon strains (Fig 5F).

**Deleted:** Integrating the genotyping results using primer sets to identify this large deletion, we To identify this putative? large deletion we successfully genotyped the 62 strains using primer sets which integrated these genotyping results.

**Deleted:** The f

**Deleted:** F

**Deleted:** still had with no observable PCR amplification product products

**Deleted:** new integrated

**Deleted:** ay

**Deleted:** in the PCR

**Deleted:** possibly

**Deleted:** e sequence

**Deleted:** by unequal crossing-over

**Deleted:** (S8 Fig)

**Deleted:** *LQGH1*

**Deleted:** were found

**Deleted:** *LQGH1*

**Deleted:** ,

**Deleted:** respectively,

**Deleted:** ,

**Deleted:** respectively,

**Deleted:** 4

## Discussion

### GH1G5 mediates quercetin uptake in *Bombyx mori*

Here, we identified GH1G5 as a glycoside hydrolase that initiates quercetin metabolism in the domestic silkworm (Fig 6). GH1G5, a putative secretory enzyme belonging to GH1, is expressed specifically in the midgut (S4 Fig). Knockout of *GH1G5* reduced total flavonoid content in the midgut, hemolymph, and silk glands, indicating that the gene is involved in quercetin absorption in the midgut, the initial place of entry into the insect (Fig 2C–2F). The hydrolytic activity of the midgut tissue on rutin, Q3MG, and Q3G was reduced in *GH1G5*-knockout mutants (Fig 3B), suggesting that GH1G5 hydrolyses all three major quercetin glycosides present in mulberry leaf. The amount of flavonoids contained in the cocoon of insects reared with a diet supplemented with rutin was greatly reduced in the absence of functional GH1G5 (Fig 3C and 3D), suggesting that deglycosylation of quercetin glycosides by GH1G5 is a critical step in quercetin uptake in the silkworm. The absorbed quercetin may be retainend in midgut cells by decreasing its lipophilicity through 5-*O*-position glucosylation by UGT [14]. The present analyses were limited to studying functional changes in deletion mutants produced by genome editing and spontaneous mutations found by screening diverse domestic silkworm strains. Further biochemical and/or immunological studies will be needed to clarify detailed enzymatic characteristics and biological roles of GH1G5.

**Deleted:** ¶
**Fig 4. Conserved structural mutations of *LQGH1* in the domestic silkworm population.**¶
(A) Dot-plot analysis of the genomic region of exons 5, 6 and the intronic region between them (intron 5) of *LQGH1* from the p50T and J01 strains. (B) PCR identifying the J01-type insertion. The amplified region is indicated by the colored area in (A). The primer set amplifying the genomic region from exons 1 and 2 of *KWMTBOMO14639* (*rp49*) was used as the control (predicted fragment length = 1548 bp). (C) Schematic illustration comparing the *Gd* loci of the p50T and Kosetsu strains. Black boxes indicate the modeled genes. (D) PCR identifying the Kosetsu-type large deletion. The amplified region is indicated by the colored area in (C). (E) RT-PCR of the *LQGH1* full-length open reading frame by using cDNA libraries constructed using from the midguts of sixth-day final instar larvae. The primer set targeting exons 1 and 2 of *KWMTBOMO14639* (*rp49*) was used as the control (predicted fragment length = 213 bp). (F) Distribution of the haplotypes of *LQGH1* in Japanese and Chinese local silkworm strains. P/P, only the p50T genotype was detected; P/J/K, p50T, J01, and Kosetsu genotypes were detected; J/J, only the J01 genotype was detected; J/K, both J01 and ... [3]

**Deleted: LQGH1**

**Deleted:** LQGH1

**Deleted:** glycosidase

**Deleted:** LQGH1

**Deleted:** and is indicated to was found to be localize ... [4]

**Deleted:** S2 and

**Deleted:** *LQGH1*

**Deleted:** uptake into

**Deleted:** *LQGH1*

**Deleted:** indicating

**Deleted:** LQGH1

**Deleted:** The greater rutin hydrolytic activity in the r ... [5]

**Deleted:** LQGH1

**Deleted:** LQGH1

**Deleted:** However, t

**Deleted:** functional

**Formatted:** Font: Italic

**Deleted:** genetic analyses

**Deleted:** using

**Deleted:** natural/

**Deleted:** deletion

**Deleted:** commercial

**Deleted:** the

**Deleted:** (

**Deleted:** )

**Deleted:** LQGH1

**Fig 6. Proposed model of quercetin uptake in the silkworm midgut.**

While revealing a major role for GH1G5 in quercetin uptake in the silkworm, this study also suggests the presence of GH1G5-independent quercetin uptake pathways. The accumulation of flavonoids in the cocoon and tissues was not completely lost in the *GH1G5*-knockout mutants (Fig 2F). In addition, the residual hydrolytic activity of the midgut homogenate on Q3MG and Q3G implies the presence of other glycoside hydrolases mediating quercetin uptake (Fig 3B). Although we were unsuccessful in obtaining knockout lineages for *GH1G1* or *GH1G9*, the proteins they encode may correspond to these other glycoside hydrolases.

**Adaptive significance of quercetin uptake in the mulberry silkworms is supported by the fact that they have retained *GH1G5***

Our results indicate that deglycosylation-dependent flavonoid uptake, which has been well-studied in mammals, is also found in insects. One feature distinguishing the mechanism in the domestic silkworm from that in other animals is the suggested rutin hydrolytic activity of GH1G5 (Fig 3B–3D). Rutin hydrolysis in mammals is dependent on the intestinal microbiota [35, 36]. Rutin glycoside hydrolases have previously been identified in bacteria, fungi, and plants [37,38,39], but to our knowledge, not in animals. Rutin hydrolysis in the honey bee (*Apis mellifera*) also depends on the gut microbiota [40], indicating that endogenous rutin glycoside hydrolases are not common in insects. Rutin is a common plant flavonoid, accounting for 14%–46% of flavonol glycosides in mulberry leaf [17]. The accumulation of flavonoids was severely restricted in the cocoons of *GH1G5*-knockout mutants reared on a rutin-containing diet, suggesting the absence of rutin digestion pathways other than deglycosylation by GH1G5 (Fig 3C and 3D). Therefore, the acquisition of rutin hydrolytic activity should result in a considerable

**Deleted:**
**Deleted:** LQGH1
**Deleted:** LQGH1
**Deleted:** glycoside digestion
**Deleted:** *LQGH1*
**Deleted:** In addition, the residual hydrolytic activity of the insoluble material fraction of the midgut on Q3MG an… [6]
**Deleted:** unable
**Deleted:** to
**Deleted:** ly
**Deleted:** in which
**Deleted:** *KWMTBOMO12222*
**Deleted:** and
**Deleted:** *KWMTBOMO12236*
**Deleted:** were knocked out
**Deleted:** ir
**Deleted:** are possibly the identities of
**Deleted:** glycosidase
**Deleted:** . In addition, the hydrolytic activity of Q3M… [7]
**Deleted: LQGH1**
**Deleted: has**
**Deleted: acquired**
**Deleted: and conserved rutin hydrolytic activity**
**Formatted:** Font: Italic
**Deleted:** show
**Deleted:** LQGH1
**Deleted:** mediates quercetin
**Deleted:** in the domestic silkworm in a deglycosylati… [8]
**Deleted:** However, our data indicate that *LQGH1* is … [9]
**Deleted:** , S10 and S11 Figs, S1 Text
**Deleted:** *In vitro*, LPH hydrolyses hydrolyzes Q3M… [10]
**Deleted:** 2
**Deleted:** It also depends on the gut microbiota in th… [11]
**Deleted:** glycosidase
**Deleted:** 4
**Deleted:** 5
**Deleted:** 6
**Deleted:** *LQGH1*
**Deleted:** was severely restricted
**Deleted:** indicating
**Deleted:** LQGH1

change in quercetin intake. In addition, the genomic instability of the *Gd* locus emphasizes the significance of quercetin bioavailability brought about by the enzyme. According to the inference of gene duplication events by OrthoFinder [32], the group G glycoside hydrolases are the group that has undergone the most frequent gene duplications among the 54 *B. mori* glycoside hydrolase ortholog groups (S4 and S5 Table). The fact that the large deletion was introduced into the *Gd* locus within a short period of the breeding history of the domestic silkworm also suggests the genomic instability of the region. While the genomic instability of the *Gd* locus had possibly provided genetic material to evolve *GH1G5* by gene duplication events, it can be inferred to have repeatedly exposed *GH1G5* to genomic structural changes which introduce defective mutations into the gene. However, the mulberry silkworms have retained *GH1G5* and its rutin hydrolytic activity until defective mutations were selected in breeding for cocoon improvement. These results support that the suggested positive properties of quercetin glycosides, notably, their antioxidant [15], ultraviolet light-protective [14], and antibacterial functions [16], improve the fitness of the wild silkworm in the natural environment. Although, it should be noted that excessive quercetin uptake is still toxic even for the domestic silkworm [41], meaning that the mulberry silkworms should have a mechanism to maintain appropriate levels of quercetin. The fluorescence in the posterior region of the midgut observed in the present study implies such mechanisms may involve accumulation of quercetin glucosides with glycosylation at the 5-*O* position and reloading of excessive quercetin glucosides from the hemolymph for excretion into the midgut lumen (Fig 2C).

**Deleted:** In addition, the phylogenetic tree of the lepidopteran proteins indicated that frequent gene duplication events have occurred in the Gd glycosidase family have occurred (S11 Fig)
**Deleted:** In
**Deleted:** [or “based on”]
**Deleted:** among 55 ortholog groups
**Deleted:** f
**Deleted:** 7
**Deleted:** the the number of events in the LQGH1-included group was contained the most frequent events, among 55 ortholog groups including 78 silkworm proteins annotated as glycosidases
**Deleted:** 5
**Deleted:** 6
**Formatted:** Font: Italic
**Formatted:** Font: Not Italic
**Formatted:** Font: Not Italic
**Deleted:** s
**Deleted:** *LQGH1*
**Deleted:** *LQGH1*
**Deleted:** a crisis of loss by unequal crossing-over
**Deleted:** *LQGH1*
**Deleted:** remained conserved
**Deleted:** its
**Deleted:** was
**Deleted:** [perhaps indirectly]
**Deleted:** [
**Deleted:** color]
**Deleted:** is emphasizes the significance of quercetin bioavailability brought about by *LQGH1*; our study
**Deleted:** s
**Deleted:** 38
**Deleted:** implied
**Deleted:** a
**Deleted:** mechanism [or “
**Deleted:** ”]
**Deleted:** d
**Deleted:** the
**Deleted:** to
**Deleted:** e them

The phylogeny for the lepidopteran orthologous proteins of the group G glycoside hydrolases suggested that *GH1G5* has evolved after the divergence of Pyraloidea and Macroheterocera (Fig 4A and 4B). This indicates that quercetin uptake mediation by GH1G5 is not common in lepidopteran insects. Given the low sequence identities (<58%) between BmorGH1G5 and the GH1G5-class proteins in other Macroheteroceran moths excluding *B. mandarina* (Fig 4C), the rutin hydrolytic activity might be conserved only in the mulberry silkworms. Although we did not investigate rutin hydrolytic activities of the GH1G5-class proteins other than BmorGH1G5, such an investigation could highlight the distinctiveness of the enzymatic property of BmorGH1G5. If so, it would allow for a discussion on the association between the evolution of the rutin hydrolytic activity and the monophagy of the silkworms on mulberry leaves, which contain high levels of rutin [17]. Quercetin uptake is observed in other herbivorous insects [9, 10]; their GH1 proteins may have evolved to adapt to flavonoid compositions in their respective host plants. A species-wide characterization of GH1 proteins in flavonoid glycoside hydrolytic activities would potentially provide insights into dietary habits of herbivorous insects.

**_GH1G5_ loss contributed to cocoon improvement**

Through breeding, many strains of the domestic silkworm have been established which exhibit a wide variety of cocoon colors depending on their flavonoid and carotenoid content. Nevertheless, white cocoons tend to be preferred commercially in regions such as Japan and China, especially since the 20th century. In a previous study that conducted a worldwide genetic analysis of silkworm strains, 91 of the 121 strains examined were white-cocoon strains [42]. The defective mutation in *GH1G5*, which manifests as reduced cocoon color (Fig 2B), may have contributed to the establishment of these white-cocoon

**Deleted:** ***LQGH1***

**Deleted:** that

**Deleted:** , but

**Deleted:** n

**Deleted:** commercially

**Deleted:** 39

**Deleted:** *LQGH1*

silkworm strains. Of the white-cocoon strains examined in the present study, only 62% carried homozygous defective haplotypes of *GH1G5* (haplotype J or K; Fig 5F, S10 Fig, and S6 Table). In 24% of the strains, a mixture of defective and functional haplotypes (haplotype P) was present. These are not surprising results, considering that not all white-cocoon strains harbor loss-of-function mutations of the sugar transporters encoded in the *Gn* locus, which is more effective than *Gd* with respect to cocoon discoloration [18]. Even if visually determined as white, the actual color tone and therefore the flavonoid content of the cocoon varies from strain to strain [43]. In addition to *Gd*, *Gb*, and *Gn*, within each of which a gene involved in the quercetin metabolism has been identified, several loci associated with cocoon greenness have been reported, including *Gc* [21,22], *Green cocoon* (*Grc*), *Green egg shell* (*Gre*), and *Yellow fluorescent* (*Yf*) [21]. "White cocoon" is presumed to be expressed by additive cocoon color discoloration by a combination of loss-of-function haplotypes of these loci.

**Deleted:** the proportion with
**Deleted:** *LQGH1*
**Deleted:** ) was only 62%
**Deleted:** (
**Deleted:** 4
**Deleted:** 8
**Deleted:** 4
**Deleted:** o
**Deleted:** 0

Although loss of *GH1G5* is not necessarily essential for cocoon whiteness, given the high conservation rate of loss-of-function haplotypes and the fact that knockout of the gene reduces the cocoon total flavonoid content by less than half (Fig 2F, Fig 5F), the gene still makes an important contribution to cocoon discoloration in the domestic silkworm. Interestingly, the knockout of *GH1G5* increased the cocoon weight (S7 Fig). Because endoreduplication is an important step in silkworm silk gland development [44], interference with the replication step by quercetin interaction with DNA may explain the relationship between *GH1G5* and silk yield [45]. Although it will be difficult to confirm this hypothesis because cocoon weight is a complex quantitative trait dependent on

**Deleted:** *LQGH1*
**Deleted:** reduced
**Deleted:** 4
**Deleted:** *LQGH1*
**Deleted:** 5
**Deleted:** 1
**Deleted:** *LQGH1*
**Deleted:** 2

silkworm physiology and behavior, elucidation of the relationship between quercetin and silk yield may provide clues to further improve silkworm protein productivity.

## Materials and Methods

Deleted: ¶

### Insect materials

All domestic silkworm strains used in the present study were maintained at the National Agriculture and Food Research Organization (NARO, Japan). p50T, the strain used for the functional analysis of *GH1G5*, is almost genetically identical to p50, because it is a descendant of p50 which had undergone repeated passage using a single pair to improve homozygosity for referential use. The silkworms were reared on a commercial artificial diet (SilkMate PM; Nosan, Kanagawa, Japan) or fresh mulberry leaves under a controlled environment (12-h light/dark photoperiod, 25°C). Insects reared on fresh mulberry leaves were used for measurement of the flavonoid contents of the cocoons of p50 and J01 (Fig 1A and 1B), the scoring for QTL analysis (Fig 1C), and measurement of the flavonoid contents of the cocoons of p50T and *GH1G5*-knockout mutants (S5 Fig). All individuals were female except those from which genomic DNA and RNA-seq data were derived.

Deleted: *LQGH1*
Deleted: the strain
Deleted: strain
Deleted: that
Deleted: *LQGH1*
Deleted: 3
Deleted: ,
Deleted: , were female

### Flavonoid content measurement

After shredding the cocoons into 2–3-mm squares, flavonoids were extracted from the pieces with MeOH–$H_2O$ (7:3, V/V) at 60ºC for 2 h. The extraction was repeated twice for thorough extraction. The flavonoid content in the solution was derived from the absorption at 365 nm, which is highly correlated with flavonoid content ($r$ = 0.95) [19]. To use flavonoid content as an input for QTL analysis, the raw absorbance values were used as relative phenotype scores. The tissues and organs for measuring flavonoid content were sampled from sixth-day final instar larvae reared on a commercial artificial diet, rinsed well with PBS buffer (pH 7.4) (Takara Bio, Shiga, Japan), and stored at −80ºC. To inhibit melanization in hemolymph, immediately after collection 0.5 M sodium

Deleted: F
Deleted: in cocoons
Deleted: , after shredding the cocoons into 2–3-mm squares
Deleted: In the case of
Deleted: of the absorbance
Deleted: the
Deleted: I
Deleted: ,
Deleted: to inhibit melanization,

dimethyldithiocarbamate (Fujifilm, Tokyo, Japan) was added to reach 0.5% by volume. Quantification of flavonoid content in the samples was performed according to a previous report [15] with some modifications. In brief, 30 μL of sample was injected into an LC-10ADVP HPLC system equipped with an SPD-10AV photodiode array detector (both Shimadzu Co., Kyoto, Japan) and separated with a Sunfire C18 column (150 × 3.0 mm i.d.; Waters, Massachusetts, USA) at a flow rate of 1.0 mL/min. Spectrum analysis and flavonoid peak assignment were performed with the CLASS-VP HPLC software (Shimadzu Co.). Flavonoids were quantified using quercetin 5, 4′-di-*O*-glucoside as the reference compound.

**QTL analysis**

The intercross F2 population of p50T and J01 used in the present study were the same samples as those sequenced by double-digest restriction-associated DNA sequencing and used to generate linkage maps in our previous study [33]. The sequencing data of 102 female F2 individuals are available in the Sequence Read Archive under the BioProject accession number PRJDB13956. The raw values of absorbance indicating the relative cocoon flavonoid content for each individual are summarized in S1 Table. The method for genotyping, marker selection, and construction of linkage maps is described in detail in our previous report [33]. Briefly, OneMap v2.8.2 [46] was used to construct linkage maps with an LOD score of 3, with the genotype of the F2 population output from the script “ref_map.pl” in STACKS v1.48 [47] as the input. The p50T genome assembly was used as the reference for read mapping [23]. The linkage map consisted of 1038 markers covering a total genetic length of 945.36 cM, with an average marker density of 0.936 cM. QTL analysis was performed using the R/qtl v1.46.2 package [48], with the relative

**Deleted:** to the collected hemolymph
**Deleted:** 0
**Deleted:** ,
**Deleted:** ,
**Deleted:** 0
**Deleted:** 3
**Deleted:** 4
**Deleted:** used
**Deleted:** ,
**Deleted:** covered
**Deleted:** and had
**Deleted:** 5

flavonoid content scores in the cocoons and the genetic position of markers linked to the physical position on the p50T genomic sequence as inputs. Using the "calc.genoprob" function in R/qtl, the probabilities of the true underlying genotypes were calculated with a step size of 1 cM and an assumed genotyping error rate of 0.05. QTL detection was performed by composite interval mapping using the function "cim" with the following parameters: method = "hk", n.marcovar = 3, window = 10. The LOD significance threshold for detecting QTL was calculated by a permutation test of 1000 trials. Approximate Bayesian 95% credible intervals were calculated using the function "bayesint". The positions of the nearest markers outside of the boundaries of a confidence interval were defined as the ends of the interval. PVEs and additive effects of the three significant QTLs were estimated using the function "fitqtl" with the parameter: formula = y ~ Q1 + Q2 + Q3 + Q1 * Q2 * Q3.

**Phylogenetic analysis**

GH1 proteins in *B. mori* were identified using InterPro annotation (InterPro entry: IPR001360, Glycoside hydrolase family 1) [49]. An InterPro annotation result for the p50T predicted protein sequences is available in KAIKObase [50] (https://kaikobase.dna.affrc.go.jp/index.html). The related proteins in Holometabola insects were collected by BLASTp search using the 21 *B. mori* GH1 proteins as the query. Genes with e-values less than 1E-50 were used for the analysis. InsectBase 2.0 (http://v2.insect-genome.com/) [51] was used for picking related proteins in *Drosophila melanogaster*, *Tribolium castaneum*, and *Apis mellifera*. Arabidopsis thioglucoside glucohydrolase 1 (TGG1) was used as an outgroup. The query and these sequences were aligned using Clustal Omega v1.2.4 [52]. Tree construction was performed by the

**Formatted:** Font: Italic
**Formatted:** Font: Italic
**Field Code Changed**
**Formatted:** Font: Italic
**Field Code Changed**

neighbor-joining method using MEGA 11 [53] with the number of bootstrap trials set to 100. The phylogenetic tree of lepidopteran orthologs of the *Gd* glycoside hydrolases was constructed by the maximum likelihood method using RAxML-NG v1.1 [54] with the following parameters: --bs-trees 100, --model LG+I+G4 (S11 Fig A). The best-fit phylogenetic analysis model was suggested using ModelTest-NG v0.1.7 [55]. Gene models of 12 lepidopteran insects available in InsectBase 2.0 were used (*Papilio Xuthus*, *Megathymus ursus*, *Lycaena phlaeas*, *Limenitis camilla*, *Chilo suppressalis*, *Biston betularia*, *Pheosia gnoma*, *Dendrolimus punctatus*, *Bombyx mori*, *Bombyx mandarina*, *Antheraea yamamai*, *Deilephila porcellus*). Each of the models was predicted based on high-quality genome sequences using long-read sequencing, and their BUSCO [56] scores were all above 94%. The tree included a total of 90 lepidopteran protein sequences, which were inferred belong to the same ortholog groups as the nine group-G glycoside hydrolases by OrthoFinder [32] v2.5.2, and Rat LPH (NCBI Refseq accession: NP_446293) as an outgroup.

## Gene editing

CHOPCHOP v3 was used to identify target sites [57]. Each 20-nucleotide guide sequence was unique on the p50T genomic sequence [23]. The construction of DNA templates for the synthesis of single-guide RNA (sgRNA) was performed according to a previous report [58]. Briefly, the DNA template, which included the T7 promoter, sgRNA target with PAM motif, and tracrRNA-complemental sequence, was synthesized by template-free PCR. A MEGAshortscript T7 Transcription Kit (Thermo Fisher Scientific, Waltham, Massachusetts, U.S.A.) was used for *in vitro* sgRNA transcription and template DNA digestion. A Guide-it IVT RNA Clean-Up Kit (Takara Bio) was used to clean up the

**Formatted:** Not Highlight

**Deleted:** 4

**Deleted:** 6

**Deleted:** 4

**Deleted:** 7

sgRNA solution. Alt-RS.p. HiFi Cas9 Nuclease V3 solution (Integrated DNA Technologies, Coralville, Iowa, U.S.A.) and the sgRNA were mixed with distilled water to final concentrations of 500 ng/μL and 50 ng/μL. The Cas9 nuclease and sgRNA mixture was incubated for 1 h at room temperature to allow the ribonucleoprotein to form. The ribonucleoprotein solution was microinjected into non-diapause eggs of the p50T strain within 8 h after oviposition, in accordance with a previous report [59]. Non-diapause treatment was performed by incubating eggs under 17°C short-day conditions (12-h light/dark photoperiod) until hatching. Adult moths of G0 individuals (injected generation) were crossed with wild-type moths. Individuals harboring identical heterozygous mutant haplotypes were identified by high-resolution melting (HRM) analysis using genomic DNA extracted from G1 generation molt shells harvested from cocoons. PCR for HRM analysis was performed using a KAPA HRM Fast PCR Kit (Roche, Basel, Switzerland) on a LightCycler 96 System (Roche) according to the manufacturer's instruction. Genomic DNA extraction from molt shells was performed by homogenizing the shells in a typical SDS-based lysis buffer followed by isopropanol precipitation. G2 individuals with a homozygous mutant haplotype were identified and crossed by the same method to establish the knockout lines. To confirm the presence of a mutation PCR products which included the target site were sequenced using a BigDye Terminator v3.1 Cycle Sequencing Kit and an Applied Biosystems 3130xl Genetic Analyzer (Thermo Fisher Scientific). The primer sequences used are listed in S7 Table.

**Photographing of dissected samples**

Sixth-day fifth instar larvae reared on the artificial diet were used. Photographs were collected with a DP74 camera (Olympus, Tokyo, Japan) attached to an SZX16

**Deleted:** , respectively
**Deleted:** e
**Deleted:** d solution
**Deleted:** 4
**Deleted:** 8
**Deleted:** the
**Deleted:** High
**Deleted:** (exuvia)
**Deleted:** of the G1 generations in
**Deleted:** identified individuals harboring identical heterozygous mutant haplotypes
**Deleted:** by
**Deleted:** By the same method,
**Deleted:** ,
**Deleted:** ing
**Deleted:** ing
**Deleted:** to confirm the mutation

microscope (Olympus). To inhibit melanization of collected hemolymph, 0.5 M sodium dimethyldithiocarbamate (Fujifilm) was added immediately after collection to reach 0.5% by volume. To remove fluorescent hemolymph before imaging, silk glands and whole-body samples from which silk glands had been removed were rinsed well with PBS buffer (pH 7.4) (Takara Bio). A fluorescence light source U-HGLGPS (Olympus) and ultraviolet light filter set (excitation: 330–385 nm, emission: 420 nm longpass) (Olympus) were used for imaging under ultraviolet light irradiation. Because of the loss of resolution and optical noise when the field of view was made large enough to fit whole bodies, these samples were photographed in segments and then the pictures were combined. In the photographs shown in Fig 2, no post-editing of any kind was performed other than cropping.

**Determination of *GH1G5* open reading frame sequence**

Total RNA was extracted from the midgut of sixth-day final instar larvae using ISOGEN (Nippon Gene, Tokyo, Japan) according to the manufacturer's instructions. Contaminating DNA in the RNA solution was digested using RNase-Free DNase Set (Qiagen, Hilden, Germany). The isolated RNA was purified using an RNeasy Kit (Qiagen). Equal volumes of RNA solutions from five individuals adjusted to the same concentration were mixed to obtain a single bulk sample. Reverse transcription was performed using ReverTra Ace qPCR RT Master Mix (Toyobo, Osaka, Japan) according to the manufacturer's instruction, and the final concentration of the RNA was adjusted to 10 ng/μL. KOD One PCR Master Mix -Blue- (Toyobo) was used for PCR according to the manufacturer's instructions. The conditions of preincubation, denaturation, annealing, and extension were set to 30 s at 95°C, 10 s at 98°C, 10 s at 55°C, and 5 s at 68°C, for 42

**Deleted:** Immediately after collection, to inhibit melanization, …

**Deleted:**

**Deleted:** to the collected hemolymph

**Deleted:** Silk

**Deleted:** before imaging to remove fluorescent hemolymph

**Deleted:** the

**Deleted:** body

**Deleted:** the whole-body

**Deleted:** ,

**Deleted:** , was performed

**Formatted:** Font: Bold

**Formatted:** Font: Bold, Italic

**Formatted:** Font: Bold

**Formatted:** Font: Bold

cycles. Amplification products were purified using QIAquick PCR Purification Kit (Qiagen) and cloned into a vector using Zero Blunt TOPO PCR Cloning Kit (Thermo Fisher Scientific). Sequencing was performed as described in the subsection above, gene editing. The primer sequences are listed in S7 Table.

**Isoform identification and expressional analysis**

RNA-seq data from the midgut and other organs of third-day fifth instar larvae of p50T and J01 were previously published by our research group [30,33]. The sequencing reads were trimmed by fastp v0.20.0 [60] with the following parameters: -q 20 -n 5 -l 100. Read mapping to the p50T genome assembly and transcript isoform identification were conducted using HISAT2 v2.1.0 [61] and SrtingTie2 v2.2.0 [62] with default parameters. Salmon v1.5.2 [63] was used to calculate transcripts per kilobase million scores. The modeled transcript sequence of p50T was used as the reference [23].

**Assay for hydrolytic activity of the midgut**

Each midgut of four independent fifth-day final instar larvae reared on the commercial artificial diet was collected, rinsed well with PBS buffer (pH 7.4) (Takara Bio), and immediately stored at −80ºC. Three times the weight of 10 mM phosphate/1 mM PMSF buffer (pH 7.5) as the sample was added prior to homogenization, which was throughly performed on ice, using a metal homozenizer Physcotron with a microshaft NS-4 (Microtec, Chiba, Japan). Three times the weight of a 20 mM phosphoric acid (pH 5.5) solution as the homogenate was add prior to hydrolysis, which was performed independently with each of the three substrates, rutin, Q3MG and Q3G, at a concentration of 2 mM for 4 h at 37ºC. Precedently, the enzyme-independent degradation of the three

**Deleted:** 49

**Deleted:** 50

**Deleted:** The

**Deleted:** of the reads

**Deleted:** 51

**Deleted:** 52

**Deleted:** the

**Deleted:** 53

**Deleted:** ¶

**Phylogenetic analysis**¶

To collect protein sequences for constructing the phylogenetic tree of glycosidases of mammals and insects (S10 Fig), the related proteins were collected by BLASTp search using the predicted protein sequence of *KWMTBOMO12227* of the p50T strain gene model as the query sequence [23]. Genes with e-values less than 1E-50 were used for the analysis. The KEGG [54] BLAST search service (https://www.genome.jp/tools/blast/) was used for picking related proteins in *Rattus norvegicus*, *Drosophila melanogaster*, *Tribolium castaneum*, and *Apis mellifera*. The query and these sequences were aligned using Clustal Omega v1.2.4 [55]. Tree construction was performed by the neighbor-joining method was performed using MEGA 11 [56] with. The the number of bootstrap trials was set to 100. The phylogenetic tree of lepidopteran orthologs of the *Gd* glycosidases was constructed by the maximum likelihood method using RAxML-NG v1.1 [57] with the following parameters: --bs-trees 100, --model LG+I+G4 (S11 Fig A). The best-fit phylogenetic analysis model was suggested using ModelTest-NG v0.1.7 [58]. Gene models of 12 lepidopteran insects available in InsectBase 2.0 [59] (http://v2.insect-genome.com/) were used (*Papilio Xuthus*, *Megathymus ursus*, *Lycaena phlaeas*, *Limenitis camilla*, *Chilo suppressalis*, *Biston betularia*, *Pheosia gnoma*, *Dendrolimus punctatus*, *Bombyx mori*, *Bombyx mandarina*, *Antheraea yamamai*, *Deilephila porcellus*).; each Each of the models were was predicted based on high-quality genome sequences using long-read sequencing, and their BUSCO [60] scores were all above 94%. Rat LPH (NCBI RefSeq accession: NP_446293) and the a total of 90 lepidopteran protein sequences of the lepidopteran species determined by Orthofinder [37] ... [12]

**Deleted:** M

**Deleted:** s

**Deleted:** were

**Deleted:** s

**Deleted:** and they were

**Deleted:** homogenized

**Deleted:** Centrifugation at 20,000*g* for 20 min, collection of the precipitate fraction, and washing with the buffer ... [13]

**Deleted:** Hydrolysis was performed in

**Deleted:** quercetin glycoside

**Deleted:** s

quercetin glycosides and quercetin in the reaction was examined using the homogenate deactivated by methanol (three volumes (V/V) of methanol were added to the reaction liquid). We measured the change in amounts of quercetin from the substrates at the 0 h and 4 h after the reaction. It confirmed that they were stable in the condition. Control incubations in which the substrates were omitted were also run for calibration taking into account a flavonoid content in the tissue. The reaction was stopped by adding three volumes (V/V) of methanol. After centrifugation at 20,000*g* for 10 min, the quercetin content of the supernatant was quantified with the HPLC system as described in the section "Flavonoid content measurement." Quercetin, rutin, and Q3G were obtained from Extrasynthese (Lyon, France). Q3MG was obtained from Merck (Darmstadt, Germany). Hydrolytic activity for the quercetin glycosides is expressed as nanomoles of quercetin produced per min per mg protein. The protein concentration in the enzyme preparations was measured with a commercial assay kit (Coomassie Plus, Thermo Fisher Scientific).

**Dietary administration of quercetin or rutin**

Silkworm larvae were reared on the commercial diet from hatching to the third ecdysis, then the fourth instar larvae were fed with a diet containing 25% mulberry leaf powder [64]. The newly molted fifth instar larvae were reared on semi-synthetic diets supplemented with quercetin or rutin, which did not contain mulberry leaf powder (S3 Table). Diets were supplemented with flavonoids in approximately equimolar (0.1 mmol/100-g dry diet) amounts, and chlorogenic acid was added as a feeding stimulant. Soybean meal (Soya flour FT; Nisshin OilliO Group,. Ltd., Tokyo, Japan) was washed twice with five volumes of 90% (V/V) ethanol to remove deterrent substances and allowed to dry naturally before use in the diets.

**Moved down [1]:** The reaction was stopped by adding three volumes (V/V) of methanol.

**Moved (insertion) [1]**

**Deleted:** toward

**Deleted:** 1

**Deleted:** Flavonoids

**Deleted:** added to the diets were approximately

**Deleted:** . C

**Deleted:** to the diets

### Genomic comparison of the *Gd* locus

Dot-plot analysis was performed using FlexiDot v1.06 [65] with the following parameters: -p 1 -f 1 -A 2 -E 100. The predicted protein sequence of J01 *GH1G5* (*BMN13127*) presented here (S8B Fig) was modified from the original model, notably, the incorrect prediction of exon 1 was modified according to *KWMTBOMO12227*, and the J01 transcript sequence was obtained by using the original RNA-seq data [33]. DNA extraction from the whole body of a male larva of the domestic silkworm strain Kosetsu was performed by a standard phenol-chloroform-based method. Whole genomic sequencing was performed by an outsourcing service (Macrogen Japan Corp., Kyoto, Japan) using an Illumina NovaSeq 6000 Sequencing System (Illumina Inc., San Diego, USA). The sequencing generated 50.4 Gb of paired-end reads with a length of 151 bp. Genome assembly of the Kosetsu strain was performed using MaSuRCA v4.0.9 [66] with pseudomolecule sequences of chromosomes 15, 20, and 27 of p50T [23], which, contain the QTLs, as the assembly reference. The parameter defining 20 times genomic size "JF_SIZE" was set to 9,000,000,000. The input genomic sequencing data of the Kosetsu strain was trimmed by fastp v0.20.0 [60] before assembly with the following parameters: -q 20 -n 5 -l 100.

### PCR-based genotyping

The genomic DNA of each strain was extracted from 10 pairs of silk glands of final instar larvae by a standard phenol-chloroform-based method. The final concentration of genomic DNA was adjusted to 0.5 ng/μL. KOD One PCR Master Mix -Blue- (Toyobo) was used for PCR according to the manufacturer's instructions. The conditions of

**Deleted:** 2
**Deleted:** illustrated
**Deleted:** exon-intron structure
**Deleted:** *LQGH1*
**Deleted:** 4A
**Deleted:** (Fig 4A);
**Deleted:** 0
**Deleted:** from the whole body of a male larva
**Deleted:** typical
**Deleted:** via an outsourcing service (Macrogen Japan Corp., Kyoto, Japan)
**Deleted:** 3
**Deleted:** on which
**Deleted:** are present
**Deleted:** 46
**Deleted:** before assembly
**Deleted:** typical
**Deleted:** conditioned
**Deleted:** , Osaka, Japan
**Deleted:** [?
**Deleted:** ?]
**Deleted:** times and temperatures

preincubation, denaturation, annealing, and extension were set to 30 s at 95°C, 10 s at 98°C, 10 s at 60°C, and 45 s at 68°C, for 32 cycles. The primer sequences are listed in S7 Table.

**Reverse-transcription PCR**

Reverse transcription was performed as described in the subsection above, determination of *GH1G5* open reading frame sequence. RT-PCR (Fig 5E) was performed using KAPA HiFi HotStart ReadyMix (Roche) under conditions that resulted in 1% of the concentration of the original cDNA solution. The conditions of preincubation, denaturation, annealing, and extension were set to 180 s at 95°C, 5 s at 98°C, 10 s at 60°C, and 60 s at 72°C, for 28 cycles. The primer sequences are listed in S7 Table.

**Detecting gene duplication events**

Detecting gene duplication events in the lepidopteran glycoside hydrolases was performed using OrthoFinder v2.5.2 [32] together with the ortholog-group inference for the phylogenetic analysis. Proteins annotated as "Glycoside hydrolase superfamily (IPR017853)" or with the functional annotation "hydrolase activity, acting on glycosyl bonds (GO:0016798)," were considered to be glycoside hydrolases.

**Other informatic tools**

Handling sequences: Seqkit v2.2.0 [67]; statistical calculations: R v4.0.3 (R Foundation for Statistical Computing, Vienna, Austria); illustrating graphs: ggplot2 v3.4.1 [68] and ggpubr v0.6.0 (https://CRAN.R-project.org/package=ggpubr); alignment visualization: SnapGene Viewer (https://www.snapgene.com/); prediction of signal peptide domains:

**Deleted:** , respectively

**Deleted:** used

**Formatted:** Font: Not Bold

**Deleted:** Total RNA was extracted from the midgut of sixth-day final instar larvae using ISOGEN (Nippon Gene, Tokyo, Japan) according to the manufacturer's instructions. Contaminating DNA in the RNA solution was digested using RNase-Free DNase Set (Qiagen, Hilden, Germany). The isolated RNA was purified using an RNeasy Kit (Qiagen). Equal volumes of RNA solutions from five individuals, adjusted to the same concentration, were mixed to obtain a single bulk sample. Reverse transcription was performed using ReverTra Ace qPCR RT Master Mix (Toyobo) according to the manufacturer's instruction, and the final concentration of the RNA was conditioned adjusted to 10 ng/μL. …

**Formatted:** Font: Not Bold

**Deleted:** times and temperatures

**Deleted:** respectively,

**Deleted:** used

**Deleted:** *Gd*

**Deleted:** sosid

**Deleted:** ases

**Deleted:** f

**Deleted:** 7

**Deleted:** The glycosidase gene was identified in the domestic silkworm by referencing the annotation file of silkworm protein using InterProScan [64] within KAIKObase [65] (https://kaikobase.dna.affrc.go.jp/index.html).

**Deleted:** proteins annotated as

**Deleted:** glycosidase

**Deleted:** 6

**Deleted:** 7

**Deleted:** hydrophobic transmembrane

SignalP v6.0 [69]; illustrating structural formulae: Ketcher v2.8.0 (https://github.com/epam/ketcher); genomic browsing: Integrative Genomics Viewer v2.12.3 [70]; phylogenetic tree editing: Interactive Tree Of Life v5 [71].

## Supplementary information

Data underlying the reported results is summarized in S1 Data. Legends of supplementary figures (S1–S10 Fig) and tables (S1–S7 Table) are available at the end of the manuscript.

## Data availability

The raw genomic sequencing data of the Kosetsu strain are available in the Sequence Read Archive under the BioProject accession number PRJDB15366.


## Acknowledgements

We thank Ms. Mayuko Sakatoh for her technical assistance; Sae Furuhashi, Toshihiko Misawa, Kaoru Nakamura, Keita Hiruma, Koji Hashimoto, and Eiji Okada for their support in the silkworm rearing; Hiroki Sakai for giving instruction on the CRISPR-Cas9 system construction; Nobuto Yamada for giving instruction on the microinjection; anonymous reviewers for constructive suggestions; Dr. Goldsmith for improving English expressions. The computation was partly performed using the supercomputer of the Agriculture, Forestry and Fisheries Research Information Technology Center of the Japanese Ministry of Agriculture, Forestry and Fisheries.


## Author contributions



**Deleted:** SOSUI
**Deleted:** 1
**Deleted:** 11
**Deleted:** 8
**Deleted:** 69
**Deleted:** 0
**Deleted:** Supplementary materials are available at Figshare (doi: 10.6084/m9.figshare.23553015
**Deleted:** )
**Deleted:** S
**Deleted:** 1
**Deleted:** and text (S1 Text)
**Deleted:** also
**Deleted:** and
**Deleted:** .

RW: Conceptualization, Data Curation, Formal Analysis, Investigation, Visualization, Writing – original draft preparation, Writing – review & editing; CH: Conceptualization, Investigation, Writing – review & editing; ST: Resources, Writing – review & editing, Supervision; TI: Conceptualization, Funding Acquisition, Investigation, Project Administration, Resources; SK: Investigation; AJ: Investigation, Writing – review & editing; TT: Resources, Writing – review & editing; KYo: Resources; KYa: Supervision; HS: Funding Acquisition, Supervision

**Funding**

This work was supported by MAFF Commissioned project study on "Research project for sericultural bio-industry" Grant Number JP22680575 to R.W., T.I. and S.T. and JSPS KAKENHI Grant Numbers 21H03831 to K.Y. and H.S.. The funders had no role in study design, data collection and analysis, decision to publish, or preparation of the manuscript.

**Competing interests**

The authors declare no competing interests.

**References**

1. Cao J, Chen W, Zhang Y, Zhang Y, Zhao X. Content of Selected Flavonoids in 100 Edible Vegetables and Fruits. FSTR. 2010;16: 395–402. doi:10.3136/fstr.16.395

2. Bernays EA, Howard JJ, Champagne D, Estesen BJ. Rutin: a phagostimulant for the polyphagous acridid Schistocerca americana. Entomologia Experimentalis et Applicata. 1991;60: 19–28. doi:10.1111/j.1570-7458.1991.tb01518.x

3. Hamamura Y, Hayashiya K, Naito K-I, Matsuura K, Nishida J. Food Selection By Silkworm Larvae. Nature. 1962;194: 754–755. doi:10.1038/194754a0

4. Haribal M, Renwick JAA. Oviposition stimulants for the monarch butterfly: Flavonol glycosides from Asclepias curassavica. Phytochemistry. 1996;41: 139–144. doi:10.1016/0031-9422(95)00511-0

5. Matsuda K. Feeding Stimulation of Flavonoids for Various Leaf Beetles (Coleoptera : Chrysomelidae). Applied Entomology and Zoology. 1978;13: 228–230. doi:10.1303/aez.13.228

6. Tebayashi S, Matsuyama S, Suzuki T, Kuwahara Y, Nemoto T, Fujii K. Quercimeritrin: The Third Oviposition Stimulant of the Azuki Bean Weevil from the Host Azuki Bean. J Pestic Sci. 1995;20: 299–305. doi:10.1584/jpestics.20.299

7. Burghardt F, Knüttel H, Becker M, Fiedler K. Flavonoid wing pigments increase attractiveness of female common blue (Polyommatus icarus) butterflies to mate-searching males. Naturwissenschaften. 2000;87: 304–307. doi:10.1007/s001140050726

8. Burghardt F, Proksch P, Fiedler K. Flavonoid sequestration by the common blue butterfly Polyommatus icarus: quantitative intraspecific variation in relation to larval hostplant, sex and body size. Biochemical Systematics and Ecology. 2001;29: 875–889. doi:10.1016/S0305-1978(01)00036-9

9. Schittko U, Burghardt F, Fiedler K, Wray V, Proksch P. Sequestration and distribution of flavonoids in the common blue butterfly Polyommatus icarus reared on Trifolium repens. Phytochemistry. 1999;51: 609–614. doi:10.1016/S0031-9422(98)00746-8

10. Hopkins TL, Ahmad SA. Flavonoid wing pigments in grasshoppers. Experientia. 1991;47: 1089–1091. doi:10.1007/BF01923349

11. Fujimoto N, Hayashiya K. Studies on the pigments of cocoon. (VIII) On the classification of the silkworm races and its geographical distribution from the viewpoint of the rate of cocoon pigment contents. Journal of Insect Biotechnology and Sericology. 1961;30: 83–88. doi:10.11416/kontyushigen1930.30.83

12. Hirayama C, Kosegawa E, Tamura Y, Nakamura M. Analysis of flavonoids in the cocoon layer of the silkworm regional races by LC-MS. SANSHI-KONCHU BIOTEC. 2009;78: 57–63. doi:10.11416/konchubiotec.78.1_57

13. Sakudoh T, Sezutsu H, Nakashima T, Kobayashi I, Fujimoto H, Uchino K, et al. Carotenoid silk coloration is controlled by a carotenoid-binding protein, a product of the Yellow blood gene. Proceedings of the National Academy of Sciences. 2007;104: 8941–8946. doi:10.1073/pnas.0702860104

14. Daimon T, Hirayama C, Kanai M, Ruike Y, Meng Y, Kosegawa E, et al. The silkworm Green b locus encodes a quercetin 5-O-glucosyltransferase that produces green cocoons with UV-shielding properties. Proceedings of the National Academy of Sciences. 2010;107: 11471–11476. doi:10.1073/pnas.1000479107

15. Hirayama C, Ono H, Tamura Y, Konno K, Nakamura M. Regioselective formation of quercetin 5-O-glucoside from orally administered quercetin in the silkworm, Bombyx mori. Phytochemistry. 2008;69: 1141–1149. doi:10.1016/j.phytochem.2007.11.009

16. Kurioka A, Ishizaka H, Yamazaki M, Endo M. Antibacterial activity of cocoon shells. J Silk Sci Tech Jpn. 1999;8: 57–60. doi:10.11417/silk.8.57

17. Sugiyama M, Katsube T, Koyama A, Itamura H. Varietal Differences in the Flavonol Content of Mulberry (Morus spp.) Leaves and Genetic Analysis of Quercetin 3-(6-Malonylglucoside) for Component Breeding. J Agric Food Chem. 2013;61: 9140–9147. doi:10.1021/jf403136w

18. Lu Y, Luo J, An E, Lu B, Wei Y, Chen X, et al. Deciphering the Genetic Basis of Silkworm Cocoon Colors Provides New Insights into Biological Coloration and Phenotypic Diversification. Molecular Biology and Evolution. 2023;40: msad017. doi:10.1093/molbev/msad017

19. Hirayama C, Okada E. Simple Spectrophotometric Analysis of Flavonoids from the Cocoon Shell of the Silkworm, Bombyx mori. J Silk Sci Tech Jpn. 2014;22: 65–70. doi:10.11417/silk.22.65

20. Peterson BK, Weber JN, Kay EH, Fisher HS, Hoekstra HE. Double Digest RADseq: An Inexpensive Method for De Novo SNP Discovery and Genotyping in Model and Non-Model Species. PLOS ONE. 2012;7: e37135. doi:10.1371/journal.pone.0037135

21. Fujii H, Banno Y, Doira H, Kihara H, Kawaguchi Y. Genetical Stocks and Mutations of Bombyx mori: Important Genetic Resoueces. 2nd ed. 1998.

22. Fujimoto N. On the physiological functions of the genes concerning the formation and the translocation of the pigments of green cocoon in silkworm larvae. The Journal of Sericultural Science of Japan. 1963;32: 338–342. doi:10.11416/kontyushigen1930.32.338

23. Kawamoto M, Jouraku A, Toyoda A, Yokoi K, Minakuchi Y, Katsuma S, et al. High-quality genome assembly of the silkworm, Bombyx mori. Insect Biochemistry and Molecular Biology. 2019;107: 53–62. doi:10.1016/j.ibmb.2019.02.002

24. Cantarel BL, Coutinho PM, Rancurel C, Bernard T, Lombard V, Henrissat B. The Carbohydrate-Active EnZymes database (CAZy): an expert resource for Glycogenomics. Nucleic Acids Research. 2009;37: D233–D238. doi:10.1093/nar/gkn663

25. Day AJ, Cañada FJ, Díaz JC, Kroon PA, Mclauchlan R, Faulds CB, et al. Dietary flavonoid and isoflavone glycosides are hydrolysed by the lactase site of lactase phlorizin hydrolase. FEBS Letters. 2000;468: 166–170. doi:10.1016/S0014-5793(00)01211-4

26. Mantei N, Villa M, Enzler T, Wacker H, Boll W, James P, et al. Complete primary structure of human and rabbit lactase-phlorizin hydrolase: implications for biosynthesis, membrane anchoring and evolution of the enzyme. The EMBO Journal. 1988;7: 2705–2713. doi:10.1002/j.1460-2075.1988.tb03124.x

27. Németh K, Plumb GW, Berrin J-G, Juge N, Jacob R, Naim HY, et al. Deglycosylation by small intestinal epithelial cell β-glucosidases is a critical step in the absorption and metabolism of dietary flavonoid glycosides in humans. Eur J Nutr. 2003;42: 29–42. doi:10.1007/s00394-003-0397-3

28. Wacker H, Keller P, Falchetto R, Legler G, Semenza G. Location of the two catalytic sites in intestinal lactase-phlorizin hydrolase. Comparison with sucrase-isomaltase and with other glycosidases, the membrane anchor of lactase-phlorizin hydrolase. Journal of Biological Chemistry. 1992;267: 18744–18752. doi:10.1016/S0021-9258(19)37024-3

29. Tamura Y, Nakajima K, Nagayasu K, Takabayashi C. Flavonoid 5-glucosides from the cocoon shell of the silkworm, Bombyx mori. Phytochemistry. 2002;59: 275–278. doi:10.1016/S0031-9422(01)00477-0

30. Yokoi K, Tsubota T, Jouraku A, Sezutsu H, Bono H. Reference Transcriptome Data in Silkworm Bombyx mori. Insects. 2021;12: 519. doi:10.3390/insects12060519

31. Fujimoto N, Hayashiya K. Studies on the pigments of cocoon. (IX) The precursor of the pigments of green cocoon in the silkworm, Bombyx mori. Journal of Insect Biotechnology and Sericology. 1972;41: 383–386. doi:10.11416/kontyushigen1930.41.383

32. Emms DM, Kelly S. OrthoFinder: phylogenetic orthology inference for comparative genomics. Genome Biology. 2019;20: 238. doi:10.1186/s13059-019-1832-y

33. Waizumi R, Tsubota T, Jouraku A, Kuwazaki S, Yokoi K, Iizuka T, et al. Highly accurate genome assembly of an improved high-yielding silkworm strain, Nichi01. G3 Genes|Genomes|Genetics. 2023;13. doi:10.1093/g3journal/jkad044

34. Abe H, Fujii T, Shimada T, Mita K. Novel non-autonomous transposable elements on W chromosome of the silkworm, Bombyx mori. J Genet. 2010;89: 375–387. doi:10.1007/s12041-010-0049-3

35. Ioku K, Pongpiriyadacha Y, Konishi Y, Takei Y, Nakatani N, Terao J. beta-Glucosidase activity in the rat small intestine toward quercetin monoglucosides. Biosci Biotechnol Biochem. 1998;62: 1428–1431. doi:10.1271/bbb.62.1428

36. Riva A, Kolimár D, Spittler A, Wisgrill L, Herbold CW, Abrankó L, et al. Conversion of Rutin, a Prevalent Dietary Flavonol, by the Human Gut Microbiota. Frontiers in Microbiology. 2020;11. Available: https://www.frontiersin.org/articles/10.3389/fmicb.2020.585428

37. Beekwilder J, Marcozzi D, Vecchi S, de Vos R, Janssen P, Francke C, et al. Characterization of Rhamnosidases from Lactobacillus plantarum and Lactobacillus acidophilus. Applied and Environmental Microbiology. 2009;75: 3447–3454. doi:10.1128/AEM.02675-08

38. Pachl P, Kapešová J, Brynda J, Biedermannová L, Pelantová H, Bojarová P, et al. Rutinosidase from Aspergillus niger: crystal structure and insight into the enzymatic activity. The FEBS Journal. 2020;287: 3315–3327. doi:10.1111/febs.15208

39. Sun Y, Cui X, Wang Z. Characterization of a rutin-hydrolyzing enzyme with β-glucosidase activity from tartary buckwheat (Fagopyrum tartaricum) seeds. Food Sci Technol. 2022;42: e42822. doi:10.1590/fst.42822

40. Kešnerová L, Mars RAT, Ellegaard KM, Troilo M, Sauer U, Engel P. Disentangling metabolic functions of bacteria in the honey bee gut. PLOS Biology. 2017;15: e2003467. doi:10.1371/journal.pbio.2003467

41. Shi G, Kang Z, Ren F, Zhou Y, Guo P. Effects of Quercetin on the Growth and Expression of Immune-Pathway-Related Genes in Silkworm (Lepidoptera: Bombycidae). Bendena B, editor. Journal of Insect Science. 2020;20: 23. doi:10.1093/jisesa/ieaa124

42. Xiang H, Liu X, Li M, Zhu Y, Wang L, Cui Y, et al. The evolutionary road from wild moth to domestic silkworm. Nat Ecol Evol. 2018;2: 1268–1279. doi:10.1038/s41559-018-0593-4

43. Hirayama C, Kosegawa E. Identification of fluorescence in white cocoon shells of the silkworm based on three-dimensional fluorescence spectroscopy. SANSHI-KONCHU BIOTEC. 2021;90: 189–194. doi:10.11416/konchubiotec.90.3_189

44. Perdrix-Gillot S. DNA synthesis and endomitoses in the giant nuclci of the silkgland of Bombyx mori. Biochimie. 1979;61: 171–204. doi:10.1016/S0300-9084(79)80066-8

45. Srivastava S, Somasagara RR, Hegde M, Nishana M, Tadi SK, Srivastava M, et al. Quercetin, a Natural Flavonoid Interacts with DNA, Arrests Cell Cycle and Causes Tumor Regression by Activating Mitochondrial Pathway of Apoptosis. Sci Rep. 2016;6: 24049. doi:10.1038/srep24049

46. Margarido GRA, Souza AP, Garcia A a. F. OneMap: software for genetic mapping in outcrossing species. Hereditas. 2007;144: 78–79. doi:10.1111/j.2007.0018-0661.02000.x

47. Catchen JM, Amores A, Hohenlohe P, Cresko W, Postlethwait JH. Stacks: Building and Genotyping Loci De Novo From Short-Read Sequences. G3 Genes|Genomes|Genetics. 2011;1: 171–182. doi:10.1534/g3.111.000240

48. Broman KW, Wu H, Sen Ś, Churchill GA. R/qtl: QTL mapping in experimental crosses. Bioinformatics. 2003;19: 889–890. doi:10.1093/bioinformatics/btg112

49. Zdobnov EM, Apweiler R. InterProScan – an integration platform for the signature-recognition methods in InterPro. Bioinformatics. 2001;17: 847–848. doi:10.1093/bioinformatics/17.9.847

50. Yang C, Yokoi K, Yamamoto K, Jouraku A. An update of KAIKObase, the silkworm genome database. Database. 2021;2021: baaa099. doi:10.1093/database/baaa099

51. Mei Y, Jing D, Tang S, Chen X, Chen H, Duanmu H, et al. InsectBase 2.0: a comprehensive gene resource for insects. Nucleic Acids Research. 2022;50: D1040–D1045. doi:10.1093/nar/gkab1090

52. Sievers F, Wilm A, Dineen D, Gibson TJ, Karplus K, Li W, et al. Fast, scalable generation of high-quality protein multiple sequence alignments using Clustal Omega. Molecular Systems Biology. 2011;7: 539. doi:10.1038/msb.2011.75

53. Tamura K, Stecher G, Kumar S. MEGA11: Molecular Evolutionary Genetics Analysis Version 11. Molecular Biology and Evolution. 2021;38: 3022–3027. doi:10.1093/molbev/msab120

54. Kozlov AM, Darriba D, Flouri T, Morel B, Stamatakis A. RAxML-NG: a fast, scalable and user-friendly tool for maximum likelihood phylogenetic inference. Wren J, editor. Bioinformatics. 2019;35: 4453–4455. doi:10.1093/bioinformatics/btz305

55. Darriba D, Posada D, Kozlov AM, Stamatakis A, Morel B, Flouri T. ModelTest-NG: A New and Scalable Tool for the Selection of DNA and Protein Evolutionary Models.

Crandall K, editor. Molecular Biology and Evolution. 2020;37: 291–294. doi:10.1093/molbev/msz189

56. Manni M, Berkeley MR, Seppey M, Zdobnov EM. BUSCO: Assessing Genomic Data Quality and Beyond. Current Protocols. 2021;1: e323. doi:10.1002/cpz1.323

57. Labun K, Montague TG, Krause M, Torres Cleuren YN, Tjeldnes H, Valen E. CHOPCHOP v3: expanding the CRISPR web toolbox beyond genome editing. Nucleic Acids Research. 2019;47: W171–W174. doi:10.1093/nar/gkz365

58. Kistler KE, Vosshall LB, Matthews BJ. Genome Engineering with CRISPR-Cas9 in the Mosquito Aedes aegypti. Cell Reports. 2015;11: 51–60. doi:10.1016/j.celrep.2015.03.009

59. Tamura T, Thibert C, Royer C, Kanda T, Abraham E, Kamba M, et al. Germline transformation of the silkworm Bombyx mori L. using a piggyBac transposon-derived vector. Nat Biotechnol. 2000;18: 81–84. doi:10.1038/71978

60. Chen S, Zhou Y, Chen Y, Gu J. fastp: an ultra-fast all-in-one FASTQ preprocessor. Bioinformatics. 2018;34: i884–i890. doi:10.1093/bioinformatics/bty560

61. Kim D, Paggi JM, Park C, Bennett C, Salzberg SL. Graph-based genome alignment and genotyping with HISAT2 and HISAT-genotype. Nat Biotechnol. 2019;37: 907–915. doi:10.1038/s41587-019-0201-4

62. Kovaka S, Zimin AV, Pertea GM, Razaghi R, Salzberg SL, Pertea M. Transcriptome assembly from long-read RNA-seq alignments with StringTie2. Genome Biology. 2019;20: 278. doi:10.1186/s13059-019-1910-1

63. Patro R, Duggal G, Love MI, Irizarry RA, Kingsford C. Salmon provides fast and bias-aware quantification of transcript expression. Nat Methods. 2017;14: 417–419. doi:10.1038/nmeth.4197

64. Hirayama C, Konno K, Shinbo H. The pathway of ammonia assimilation in the silkworm, Bombyx mori. Journal of Insect Physiology. 1997;43: 959–964. doi:10.1016/S0022-1910(97)00045-0

65. Seibt KM, Schmidt T, Heitkam T. FlexiDot: highly customizable, ambiguity-aware dotplots for visual sequence analyses. Hancock J, editor. Bioinformatics. 2018;34: 3575–3577. doi:10.1093/bioinformatics/bty395

66. Zimin AV, Marçais G, Puiu D, Roberts M, Salzberg SL, Yorke JA. The MaSuRCA genome assembler. Bioinformatics. 2013;29: 2669–2677. doi:10.1093/bioinformatics/btt476

67. Shen W, Le S, Li Y, Hu F. SeqKit: A Cross-Platform and Ultrafast Toolkit for FASTA/Q File Manipulation. PLOS ONE. 2016;11: e0163962. doi:10.1371/journal.pone.0163962

68. Wickham H. ggplot2: Elegant Graphics for Data Analysis. New York, NY: Springer; 2009. doi:10.1007/978-0-387-98141-3

69. Teufel F, Almagro Armenteros JJ, Johansen AR, Gíslason MH, Pihl SI, Tsirigos KD, et al. SignalP 6.0 predicts all five types of signal peptides using protein language models. Nat Biotechnol. 2022;40: 1023–1025. doi:10.1038/s41587-021-01156-3

70. Robinson JT, Thorvaldsdóttir H, Winckler W, Guttman M, Lander ES, Getz G, et al. Integrative genomics viewer. Nat Biotechnol. 2011;29: 24–26. doi:10.1038/nbt.1754

71. Letunic I, Bork P. Interactive Tree Of Life (iTOL) v5: an online tool for phylogenetic tree display and annotation. Nucleic Acids Research. 2021;49: W293–W296. doi:10.1093/nar/gkab301

72. Kawahara AY, Plotkin D, Espeland M, Meusemann K, Toussaint EFA, Donath A, et al. Phylogenomics reveals the evolutionary timing and pattern of butterflies and moths.



**Deleted:** ¶
24. Day AJ, Cañada FJ, Díaz JC, Kroon PA, Mclauchlan R, Faulds CB, et al. Dietary flavonoid and isoflavone glycosides are hydrolysed by the lactase site of lactase phlorizin hydrolase. FEBS Letters. 2000;468: 166–170. doi:10.1016/S0014-5793(00)01211-4¶
25. Mantei N, Villa M, Enzler T, Wacker H, Boll W, James P, et al. Complete primary structure of human and rabbit lactase-phlorizin hydrolase: implications for biosynthesis, membrane anchoring and evolution of the enzyme. The EMBO Journal. 1988;7: 2705–2713. doi:10.1002/j.1460-2075.1988.tb03124.x¶
26. Németh K, Plumb GW, Berrin J-G, Juge N, Jacob R, Naim HY, et al. Deglycosylation by small intestinal epithelial cell β-glucosidases is a critical step in the absorption and metabolism of dietary flavonoid glycosides in humans. Eur J Nutr. 2003;42: 29–42. doi:10.1007/s00394-003-0397-3¶
27. Wacker H, Keller P, Falchetto R, Legler G, Semenza G. Location of the two catalytic sites in intestinal lactase-phlorizin hydrolase. Comparison with sucrase-isomaltase and with other glycosidases, the membrane anchor of lactase-phlorizin hydrolase. Journal of Biological Chemistry. 1992;267: 18744–18752. doi:10.1016/S0021-9258(19)37024-3¶
28. Tamura Y, Nakajima K, Nagayasu K, Takabayashi C. Flavonoid 5-glucosides from the cocoon shell of the silkworm, Bombyx mori. Phytochemistry. 2002;59: 275–278. doi:10.1016/S0031-9422(01)00477-0¶
29. Fujimoto N, Hayashiya K. Studies on the pigments of cocoon. (IX) The precursor of the pigments of green cocoon in the silkworm, Bombyx mori. Journal of Insect Biotechnology and Sericology. 1972;41: 383–386. doi:10.11416/kontyushigen1930.41.383¶
30. Waizumi R, Tsubota T, Jouraku A, Kuwazaki S, Yokoi K, Iizuka T, et al. Highly accurate genome assembly of an improved high-yielding silkworm strain, Nichi01. G3 Genes|Genomes|Genetics. 2023;13. doi:10.1093/g3journal/jkad044¶
31. Ioku K, Pongpiriyadacha Y, Konishi Y, Takei Y, Nakatani N, Terao J. beta-Glucosidase activity in the rat small intestine toward quercetin monoglucosides. Biosci Biotechnol Biochem. 1998;62: 1428–1431. doi:10.1271/bbb.62.1428¶
32. Riva A, Kolimár D, Spittler A, Wisgrill L, Herbold CW, Abrankó L, et al. Conversion of Rutin, a Prevalent Dietary Flavonol, by the Human Gut Microbiota. Frontiers in Microbiology. 2020;11. Available: https://www.frontiersin.org/articles/10.3389/fmicb.2020.585428¶
33. Kešnerová L, Mars RAT, Ellegaard KM, Troilo M, Sauer U, Engel P. Disentangling metabolic functions of bac ... [14]

**Deleted:** 6

**Deleted:** 7

**Deleted:** 68. Hirokawa T, Boon-Chieng S, Mitaku S. SOSUI: classification and secondary structure prediction system for membrane proteins. Bioinformatics. 1998;14: 378–379. doi:10.1093/bioinformatics/14.4.378

**Deleted:** 69

**Deleted:** 0

**Deleted:** 1

Proceedings of the National Academy of Sciences. 2019;116: 22657–22663. doi:10.1073/pnas.1907847116

## Supplementary materials

**S1 Fig. Photographs of representative cocoons of the wild silkworm, *Bombyx mandarina*.**
A fresh mulberry leaf and cocoons of the wild silkworm in a bright field (left) and irradiated with ultraviolet light A in a dark field (right). The cocoons under ultraviolet irradiation exhibit fluorescence characteristic of quercetin-5-*O*-glucoside and quercetin-5,4′-di-*O*-glucoside, the major quercetin metabolites in the silkworm tissues and cocoon [14,15,28]. The cocoons were collected in June 2023 at Tsukuba, Ibaraki, Japan. Bar = 10 mm.

**S2 Fig. Phylogenetic tree of GH1 proteins in *B. mori* and other Holometabola insects.**
Values on the nodes represent the bootstrap score (trials = 100). Unreliable nodes with bootstrap values under 50 are shown as multi-branching nodes. Arabidopsis thioglucoside glucohydrolase 1 (TGG1) was used as the outgroup.

**S3 Fig. Similarity of the group G glycoside hydrolase proteins in *B. mori*.**
(A) Alignment of the group G glycoside hydrolase proteins in *B. mori* using Clustal Omega. Predicted signal peptides are highlighted by red lines. The threshold for identity shading was set to 80%. (B) Sequence identity and similarity of the group G glycoside hydrolase proteins in *B. mori*. Similarity of amino acid residue property is determined according to the groups of strongly similar properties described at Clustal Omega FAQ (https://www.ebi.ac.uk/seqdb/confluence/display/THD/Help+-+Clustal+Omega+FAQ).

**Deleted:** those
**Deleted:** that is
**Formatted:** Font: Italic
**Formatted:** Font: Bold
**Formatted:** Font: Italic
**Formatted:** Font: Italic
**Field Code Changed**

**S4 Fig. Expression profile of the candidate genes within the *Gd* locus in third-day final instar larva of the p50T strain.**

All organs and tissues, except for ovaries, were taken from male larvae. Data are means of independent biological replicates ± SD. SG, silk gland; TPM, transcripts per million.

**S5 Fig. Total flavonoid content in cocoons of the p50T strain and *KWMTBOMO12227*-knockout lineages reared on fresh mulberry leaves.**

Data are means of independent biological replicates ± SD.

**S6 Fig. RNA-seq read alignment to the genomic sequence of *GH1G5*.**

(A) Regions around the translation start site (up) and stop site (down) of the cloned transcript sequence of *GH1G5*. The annealing sites of primers used for cloning are highlighted by black lines. (B) Mapping results of the midgut-derived RNA-seq reads and *GH1G5* transcript isoforms visualized with Integrative Genomics Viewer. Coverage on each exon and splice junction of three biological replicates is illustrated on tracks R1–3. (C) Coverage mean of each base of exon 10 and the 15-bp extension region. Data are means of independent biological replicates ± SD.

**S7 Fig. Weight of pupae and cocoons of p50T and *GH1G5* knockout mutants.**

(A) Weight of the pupae. (B) Weight of the cocoons. The values above the graph are *p*-values calculated by Student's *t*-test. Crosses inside the boxes indicate means of independent biological replicates.

**S8 Fig. Comparison of *GH1G5* sequences between the p50T and J01 strains.**

Deleted: 2
Deleted: 3
Deleted: the
Deleted: the
Deleted: ¶
Formatted: Font: Italic
Deleted: ¶
Deleted: 5
Deleted: *LQGH1*
Deleted: the
Deleted: of *LQGH1*
Deleted: The c
Deleted: the
Deleted: ¶
Deleted: 6
Deleted: Alignment of the amino acid sequences
Deleted: *LQGH1*
Deleted: from

(A) Dot-plot analysis of the genomic region of exons 5, 6 and the intronic region between them (intron 5) of *GH1G5* from the p50T and J01 strains. Green dots indicate inverted regions. (B) Alignment of predicted protein sequences of GH1G5 from the p50T and J01 strains using Clustal Omega. (C) Deletion in the J01 *GH1G5* transcript confirmed using Sanger sequencing.

**S9 Fig. Alignment of GH1G5-like proteins in Macroheterocera species.**
(A) Whole alignment of 101 GH1G5-like proteins in Macroheterocera species. Amino acid residues identical to GH1G5 at positions with >99% conservation are highlighted by blue. The 100 homologous proteins were collected by a BLASTp search using the amino acid sequence of GH1G5 from sequence data of each protein from *Bombyx mori* (KWMT), *Antheraea yamamai* (Ayam), *Deilephila porcellus* (Dpor), *Dendrolimus punctatus* (Dpun) and *Pheosia gnoma* (Pgno) with a cutoff criterion e-value of <1E-100. The sequence data of *B. mori* were obtained from KAIKObase (https://kaikobase.dna.affrc.go.jp/index.html) and those of *A. yamamai*, *D. porcellus* and *P. gnoma* were obtained from InsectBase 2.0 (http://v2.insect-genome.com/). The sequences were aligned using Clustal Omega. (B) Magnified view of the J01-type deletion region of the alignment. Descriptions of the consensus symbols indicating the conservation level of amino acid residues are available at the Clustal Omega FAQ (https://www.ebi.ac.uk/seqdb/confluence/display/THD/Help+-+Clustal+Omega+FAQ). In brief, ':' indicates residues harboring a strongly similar physicochemical property to GH1G5, and '.' indicates residues harboring a weakly similar physicochemical property to GH1G5.

**Deleted:** Amino acid sequences were aligned
**Formatted:** Font: Italic
**Deleted:** ¶
**Deleted:** 7
**Deleted:** **LQGH1**
**Deleted:** LQGH1
**Deleted:** LQGH1
**Deleted:** LQGH1
**Deleted:** each protein
**Deleted:** of *Bombyx mori*
**Deleted:** that
**Deleted:** that in
**Deleted:** LQGH1
**Deleted:** that
**Deleted:** that in
**Deleted:** LQGH1

**S10 Fig. Genotyping of the *Gd* locus of Japanese or Chinese local white-cocoon strains.**

Haplotypes of the strains were determined by PCR genotyping. P/P, only the p50T genotype was detected; P/J/K, all p50T, J01, and Kosetsu genotypes were detected; J/J, only the J01 genotype was detected; J/K, both the J01 and Kosetsu genotypes were detected; K/K, only the Kosetsu genotype was detected; U, unknown, nothing detected. The primer set amplifying the genomic region from exons 1 to 2 of *KWMTBOMO14639* (*rp49*) was used as a control (predicted fragment length = 1548 bp). The straight grey dotted lines connect band markers of known size (M) which were applied at both ends of the samples. These lines represent sizes of 4000, 200, 500, and 1500 bp, from top to bottom. “JL” and “CL” stand for “Japanese local” and “Chinese local”. The colors indicate the color of the cocoons.

**S1 Table. Information of the F2 individuals, parents and phenotypic score used for the QTL analysis.**

**S2 Table. QTLs associated with flavonoid content in cocoons.**

**S3 Table. Composition of the semi-synthetic diets supplemented with quercetin or rutin.**

**S4 Table. Orthogroups of glycoside hydrolases in the 12 species of Lepidoptera and numbers of detected gene duplication events.**

Deleted: ¶

Deleted: 8

Deleted: the

Deleted: are straight lines

Deleted: ing

Deleted: that

Deleted: , respectively

**S5 Table. Domestic silkworm glycoside hydrolases.**

**S6 Table. Summary of the Japanese and Chinese local silkworm strains examined.**

**S7 Table. Primers used in the experiments.**

**S1 Data. Data underlying the reported results.**

Page 10: [1] Deleted 和泉 隆誠 10/25/23 2:23:00 PM

Page 10: [2] Deleted Marian Goldsmith 10/2/23 3:16:00 PM

Page 24: [3] Deleted 和泉 隆誠 10/25/23 2:21:00 PM

Page 24: [4] Deleted 和泉 隆誠 10/18/23 9:15:00 PM

Page 24: [5] Deleted 和泉 隆誠 10/18/23 9:17:00 PM

Page 26: [6] Deleted 和泉 隆誠 10/22/23 12:52:00 PM

Page 26: [7] Deleted 和泉 隆誠 10/22/23 12:53:00 PM

Page 26: [8] Deleted 和泉 隆誠 10/18/23 9:39:00 PM

Page 26: [9] Deleted 和泉 隆誠 10/18/23 9:40:00 PM

Page 26: [10] Deleted 和泉 隆誠 10/18/23 9:41:00 PM

Page 26: [11] Deleted 和泉 隆誠 10/18/23 9:41:00 PM

Page 37: [12] Deleted 和泉 隆誠 10/18/23 11:39:00 PM

Page 37: [13] Deleted 和泉 隆誠 10/25/23 12:50:00 PM

**Page 51: [14] Deleted 和泉　隆誠 10/19/23 5:19:00 PM**
