# Supplementary material for: A major endogenous glycoside hydrolase mediating quercetin uptake in Bombyx mori
Source: PLoS Genet. 2024 Jan 17;20(1):e1011118. doi: 10.1371/journal.pgen.1011118 (PMC10824415; doi:10.1371/journal.pgen.1011118)
Supplement: Attachment — Submitted filename: response_to_reviewers_rev2.pdf [file pgen.1011118.s022.pdf]

<u>Responses to comments of the three reviewers and associate editor</u>

We would like to express our deepest gratitude for reviewing our manuscript and apologize for the errors left in the previous version of our manuscript. We have carefully addressed and incorporated all the suggested revisions into the revised manuscript. In addition, according to the editor's suggestions, we have majorly revised Fig. 6, which illustrates a proposed model of quercetin metabolism in the silkworm, as well as the related parts in the manuscript. Below, we have done a point-by-point reply to the provided comments. Our responses are described in blue color.

Reviewer #1: I am satisfied by the changes made by the authors. The manuscript has been substantially improved. I see no major reason that should prevent acceptance for publication. I have a few minor comments, mostly typos, that need to be attended to.

Thank you very much for reviewing our manuscript and valuing the revision.

Minor comments:

- page 7, line 130. Title of the legend of Fig. 1, replace "Quantitive" by "Quantitative".

Thank you for pointing that out. It has been addressed (line 137).

- page 10, line 194. Add "in" before "midgut".

Thank you for pointing that out. It has been addressed (line 202).

- page 15, line 262. Replace "glycosidses" by "glycosidases".

Thank you for pointing that out. It has been addressed (line 275).

- page 24, line 423. Replace "retainend" by "retained".

Thank you for pointing that out. It has been addressed (line 465).

- page 33, line 598. Write "Arabidopsis" in italic.

Thank you for pointing that out. It has been addressed (lines 712 and 1266).

- page 37, line 678. Replace "expressional" by "expression".

Thank you for pointing that out. It has been addressed (line 807).

- page 37, line 691. Replace "throughly" by "thoroughly"

Thank you for pointing that out. It has been addressed (line 820).

- page 37, line 692. Replace "homozenizer" by "homogenizer".

Thank you for pointing that out. It has been addressed (line 821).

- page 37, line 696. Do you mean "Previously" instead of "Precedently"?

Thank you for pointing that out. We deleted "Precedently" (line 825).

- page 40, line 764. Maybe "Other bioinformatics tools" would sound better.

Thank you for your suggestion. It has been changed as such (line 922).

We apologize for leaving these typing errors. Once again, thank you for providing helpful comments which have enhanced our manuscript. We expressed our thanks to you in the "Acknowledgements" section.

Reviewer #2: I have updated details of the review indicated below as an attachment (pdf edited with Adobe Acrobat)

The authors have addressed my comments and concerns well and thoroughly. This results in giving greater impact to the phylogenetic treatment by moving the essential data and reporting and discussion of it into the main text instead of assigning it to the supplementary text, and making numerous small changes to the language better to bring it in line with common English expression. Therefore I believe the revised treatment improves the potential impact of the research on a wider audience than initially and the readability of the text.

In my re-review I recommended a number of minor edits throughout the text to clarify the information and analysis I believe the authors intend to convey, and to improve the English expression a little more. Rather than extracting these edits into a line-by-line listing here I have marked them directly on the manuscript using Acrobat Reader which can be formatted to list embedded comments in a master list. If necessary I am willing to extract this information to standard line-by-line listings for editors’ and authors’ reference. However, I believe returning a marked up text along with my comments on the reasons for the proposed changes is a more efficient and useful way to convey this information.

Otherwise my general positive comments in the initial review stand so it seems unnecessary to repeat them here.

We are sincerely grateful to you for valuing the revision and your overall proofreading of our manuscript. The suggested revisions of yours in the English expression have been appropriately presented in the revised manuscript. We expressed our thanks to you in the "Acknowledgements" section.

Reviewer #3: I am very happy with the revised manuscript and think the authors did an excellent job to address the comments. Below are some suggestions for minimal edits to the text. Line numbers refer to the version without track changes.

We sincerely thank you for reviewing our manuscript and your assistance which has greatly improved our manuscript.

Line 173: I suggest to write “secreted enzymes” instead of “secretory enzymes”. The former term is used more frequently in the literature.

Thank you for your suggestion. It has been changed as such (lines 180 and 455).

Line 221: please omit “absolutely”

Thank you for pointing that out. It has been changed to "completely" (line 229).

Line 221 (and other places in the text): I suggest to write “predicted sequence” instead of “modeled sequence”

Thank you for pointing that out. It has been addressed (lines 225, 229, 372, 388 and 814).

Line 683: correct “SrtingTie2” to “StringTie2”

Thank you for pointing that out. It has been addressed (line 812).

Once again, we deeply appreciate your assistance. We expressed our thanks to you in the "Acknowledgements" section.

Associate editor: The author has nicely revised the mauscript. In this final round of review, I have some additional comments that would strengthen the manuscript.

1. Fig. 6

I think the authors could improve Fig. 6 to make it clearer and more comprehensive. For example, the process of deglycosylation and subsequent re-glycosilation of plant-derived quercetin/flavonoids should be shown more clearly. Also, the process of uptake and excretion of flavonoids, i.e., from the gut lumen to the silk gland lumen, in the Bombyx could be described in the figure.

2. other loci

There are other loci involved in the flavonoid cocoon trait. The author may want to include such loci, e.g., Green n, Green a, and Lg/P5CR in Fig. 6.

Thank you so much for taking your time to review our manuscript and providing helpful comments. According to your suggestion, we have added a comprehensive description of quercetin metabolism in the silkworm (lines 482–526 and Fig. 6). In addition, although not mentioned by the reviewers, we have also made several minor edits as follows.

Line 6: The name of one of the authors was corrected.

Line 273: We deleted " , indicating it is the only glycoside hydrolase with hydrolytic activity against this quercetin glycoside" because our results only suggest that GH1G5 is the sole enzyme expressed in the midgut that has hydrolytic activity on rutin limited in our reaction conditions.

Lines 567, 782, 783 and 1258: For consistency in expression, we have removed the word 'light'.

Line 1339: For consistency in expression, we have changed "F2" to "F_2".

Fig. 3B: For consistency in expression, we have changed "Hydrolysis activity" to " Hydrolytic activity ".

Please also note that reviewer #2 has attached the pdf file with the possible changes to the manuscript.

The suggested revisions in the English expression have been appropriately presented in the revised manuscript.

Finally, if the authors agree, it would be nice to acknowledge the three anonymous reviewers who provided constructive and valuable suggestions in the Acknowledgments section.

Thank you for your suggestion. We are sincerely grateful to the reviewers for their invaluable support. We expressed our thanks in the "Acknowledgements" section (lines 949–951).

Again, we sincerely appreciate your assistance with the peer review of our manuscript.
