# Supplementary material for: A major endogenous glycoside hydrolase mediating quercetin uptake in Bombyx mori
Source: PLoS Genet. 2024 Jan 17;20(1):e1011118. doi: 10.1371/journal.pgen.1011118 (PMC10824415; doi:10.1371/journal.pgen.1011118)
Supplement: Attachment — Submitted filename: Review Waizumi et al, PLOSGen_MRG.docx [file pgen.1011118.s019.docx]

Overview of basic findings and their biological significance. This manuscript presents new findings about the genetic and biochemical basis of green cocoon colors produced by the domesticated silkworm, Bombyx mori. This species generally produces light beige or white colored cocoons, whereas its wild ancestor, B. mandarina, produces green cocoons. Flavonoids derived from the insects’ host plant, white mulberry (Morus alba), are responsible for the green cocoon pigment. These compounds have important effects on critical insect behaviors such as host plant recognition, feeding, mate attraction, and oviposition, and on body, wing, and cocoon coloration, which may impact other behaviors in the wild such as mating. Flavonoids are also attributed with antioxidant activity, and confer protection (as shown in silkworms) from UV exposure during metamorphosis from pupa to adult within cocoons. These and additional properties of flavonoids are found in diverse insects, including moths and butterflies, beetles, weevils, aphids, grasshoppers, and so on. In addition, antioxidant and anti-inflammatory activities of flavonoids derived from plants are considered important for human health; this confers an additional level of biological significance to this study beyond its impact on our understanding of basic genetics, biochemistry, and evolution of insects. It is also relevant that, as the authors assert, theirs is the first report of a gene encoding this class of glycoside hydrolases, known to be produced widely by bacteria and fungi, in a multicellular animal.

Outline of the study. Prior to the present research, although a few “green” loci had been defined and assigned to silkworm chromosomes using traditional “forward genetics,” the precise genomic location and structure of some remained to be determined, and knowledge of specific biochemical pathways involved in flavonoid metabolism in silkworms was incomplete. In particular, structural modification of quercetin, a key flavonoid obtained from silkworms’ main host plant, white mulberry (Morus alba), is required for its initial uptake and conversion to biologically active forms extracted from spun cocoons and details of these metabolic changes were limited.

The authors performed more comprehensive genetic mapping than before by using modern QTL techniques dependant on well-established molecular tagging to find loci which affected flavonoid content on offspring of F2 populations derived from crosses between two representative inbred silkworm strains with well-defined genome sequences which produced white (i.e., essentially no green pigment) vs green cocoons. This study uncovered 3 unlinked loci with different quantitative contributions to green cocoon color. The authors deemed two of them, based on their chromosomal locations, to have already been discovered via traditional gene mapping approaches as Gc on ch15 and Gn on ch27. The search reported here uncovered a third QTL on chromosome 20, named Gd, which had the second strongest level of phenotypic variation based on cocoon flavonoid content. The authors then examined its chromosomal region in molecular detail and identified an LPH-like quercetin glycoside hydrolase 1 (LQGH1) capable of carrying out the expected chemical modifications of quercetin in this region. The authors then used specific molecular tags designed from the enzyme sequence to characterize further the detailed DNA structure and putative function of the locus. It turned out to contain a cluster of 9 glucoside hydrolase gene models, of which three were actively transcribed in midgut, the logical primary site for action of such a gene. The authors then used CRISPR-Cas9 mutgenesis to test the function of these three candidate genes in the uptake and hydrolytic conversion of quercetin. Fortunately, mutagenesis worked well for one of the 3 gene models; however, knockout mutations of the other two were lethal as homozygotes, precluding this approach for directly confirming their putative function. Nevertheless, by comparing total flavonoid content in various larval tissues of silkworms carrying the wildtype vs the single effective CRISPR/Cas9 knockout (termed KWMTBOMO12227) the authors determined its major sites of expression (i.e., function) in hemolymph, midgut, silkgland and Malpighian tubules based on flavonoid pigment content assayed under white or UV light (which causes flavonoids to fluoresce). Further, the authors found the enzyme anchored to apical midgut cell membranes rather than in the soluble fraction of cell homogenates, which was consistent with their expectations of its localization based on the enzyme’s predicted protein structure. The authors also drilled down on the enzyme’s specific hydrolytic activity by comparing flavonoid uptake and by-products in wild type vs knockout mutants fed various quercetin glycosides in mulberry leaves or supplemented in artificial diet. Ultimately, these studies present a much more complete picture of flavonoid genetics, uptake, and utilization in silkworms than previously known.

Most of the remainder of the report is devoted to characterization and analysis of the distribution of mutations of LQGH1 in domesticated silkworm strains from Japan and China which produce white vs green-colored cocoons. Using the well established genome sequence of a green strain as a standard, the authors designed PCR primers to amplify relevant candidate target sites and uncovered insertions and deletions which disrupted the predicted functional proteins in many white-cocoon strains, but no mutations in the green ones. They also identified some novel “white” mutations which the original primers failed to amplify and characterized them in further molecular detail. Ultimately this analysis yielded 3 distinct haplotypes which were present in various homo- and heterozygous conditions among the strains surveyed: the “wild type” found in the green cocoon standard, and two mutant haplotypes, one harboring a 4 kb insertion and the other a large deletion of the entire LQGH1 gene. Unsurprisingly, the population distribution of these haplotypes differed among Japanese-local and Chinese-local silkworm strains maintained for breeding for silk production, which would have been subjected to different mating patterns and intensity of artificial selection. In this strain-distribution study the authors also made a remarkable and unexpected (to me at least) discovery that the relative silk yield was significantly greater in white vs green-cocoon strains.

An additional aspect of this research was the authors’ phylogenetic search for orthologs of their quercetin glycosidase enzyme LQGH1 across a few insect and many lepidopteran species. This survey assigned a likely evolutionary origin to the lepidopteran clade Notodontidae, which is within the largest lepidopteran superfamily, Noctuoidea, which continues to be under intense study (and some controversy) to determine its true taxonomic relationships. Relevant to further comment below, I note that the data for this information is confined to the supplement, and the authors provide only a brief description of it (lines 212-213) in the main text, with no subsequent reference to its significance in the Discussion.

Significance. The discovery and characterization of this gene, its variants, and its biochemical function make significant contributions to silkworm and insect science. The gene itself provides a new and important candidate for flavonoid metabolism in several important lepidopteran clades, although no orthologs were found in other insects. The study strongly supports the direct role of LQGH1 in contributing to the diversity of a major class of silkworm cocoon color variants, a possible indirect role in silkworm domestication and artificial selection for strain improvement, and, based on metabolic study, a strong likelihood of its essential requirement in the wild silkworm progenitor, B. mandarina.

Evaluation

Overall I found the experimental plan and detailed design of this study to be logical, comprehensive, and complete. The data are clear and presented well in support of the authors’ hypotheses. Similarly, the Materials and Methods section appears to be complete in providing essential information and access to technically important details including critical reagents.

I also found the report to be well organized and well-written. I appreciated the authors’ skill at providing brief summaries of the nature and significance of their findings throughout it, especially given its technical breadth and complexity in terms of the number of different experimental approaches they used, and the proposed significance of their findings. Regarding the writing itself, although I found a few typos (e.g., on line 512 please change “makers” to “markers”) and minor instances where I thought English expression could be improved, at the journal’s recommendation I do not list them in this review because a professional copy editor can easily find them. I would be willing to provide a marked copy if desired.

Regarding the balance of ideas presented in the report, after identifying LQGH1 the authors put considerable effort and energy into characterizing mutations of the gene (or "locus” may be a more accurate term) among domesticated regional Japanese and Chinese silkworm strains which form a foundation for sericultural work. This survey turned up some interesting features, including (as already noted) the greater size (weight) of white vs green cocoon strains, suggesting the absence of flavonoid modification pathways and/or content likely had adaptive significance during domestication and subsequent artificial breeding and selection. These data also pointed to a likelihood that other quercetin or rutin utilization pathways remain to be discovered in silkworms. Nevertheless – and this may be my bias – even though in the Discussion the authors relate these findings to questions of evolutionary conservation of flavonoid-biochemistry-related processes among insect and non-insect taxa (especially mammals), I am not sure how much interest this aspect of the report will generate outside of the silkworm or sericulture community. On the other hand, I found the phylogenetic study of glycosidase orthologs to be relevant for a potentially wide audience involved with insect (especially but not only Lepidoptera) and arthropod genomics, genetics, taxonomy, physiology, and biochemistry. Yet (again as noted) the authors placed the two figures for these data in the supplementary text, and wrote very little about it in the main text, with virtually no mention in the Discussion. Given the importance of flavonoids for insects, it seems to me the impact of this research might be enhanced by moving at least one figure for the phylogenetic study (?S11?) to the main text.

The authors may also consider adding a brief statement to the Discussion about the significance of this (and other flavonoid-pathway genes yet to be discovered?) for determining the evolution of other lepidopteran clades as they did for silkworm strains. I suggest this primarily to call their findings to the attention of phylogeneticists engaged in deciphering the Noctuidae who may find them useful models for their studies.
